# Bioinspired soft-hard combined system with mild photothermal therapeutic activity promotes diabetic bone defect healing via synergetic effects of immune activation and angiogenesis

**DOI:** 10.7150/thno.97335

**Published:** 2024-07-01

**Authors:** Minhao Wu, Huifan Liu, Yufan Zhu, Ping Wu, Yun Chen, Zhouming Deng, Xiaobin Zhu, Lin Cai

**Affiliations:** 1 Department of Spine Surgery and Musculoskeletal Tumor, Zhongnan Hospital of Wuhan University, 168 Donghu Street, Wuchang District, Wuhan 430071 Hubei, China.; 2 Department of Anesthesiology, Research Centre of Anesthesiology and Critical Care Medicine, Zhongnan Hospital of Wuhan University, Wuhan, Hubei, China.; 3 National Key laboratory of macromolecular drug development and manufacturing, School of Pharmaceutical Science, Wenzhou Medical University, Wenzhou, 325035, China.; 4 Department of Biomedical Engineering and Hubei Province Key Laboratory of Allergy and Immune Related Disease, TaiKang Medical School (School of Basic Medicine Sciences), Wuhan University, Wuhan 430071, China.

**Keywords:** 3D-printed scaffold, bioactive hydrogel, mild photothermal stimulation, immune microenvironment, angiogenesis, diabetic bone regeneration

## Abstract

**Background:** The comprehensive management of diabetic bone defects remains a substantial clinical challenge due to the hostile regenerative microenvironment characterized by aggravated inflammation, excessive reactive oxygen species (ROS), bacterial infection, impaired angiogenesis, and unbalanced bone homeostasis. Thus, an advanced multifunctional therapeutic platform capable of simultaneously achieving immune regulation, bacterial elimination, and tissue regeneration is urgently designed for augmented bone regeneration under diabetic pathological milieu.

**Methods and Results:** Herein, a photoactivated soft-hard combined scaffold system (PGCZ) was engineered by introducing polydopamine-modified zeolitic imidazolate framework-8-loaded double-network hydrogel (soft matrix component) into 3D-printed poly(ε-caprolactone) (PCL) scaffold (hard matrix component). The versatile PGCZ scaffold based on double-network hydrogel and 3D-printed PCL was thus prepared and features highly extracellular matrix-mimicking microstructure, suitable biodegradability and mechanical properties, and excellent photothermal performance, allowing long-term structural stability and mechanical support for bone regeneration. Under periodic near-infrared (NIR) irradiation, the localized photothermal effect of PGCZ triggers the on-demand release of Zn^2+^, which, together with repeated mild hyperthermia, collectively accelerates the proliferation and osteogenic differentiation of preosteoblasts and potently inhibits bacterial growth and biofilm formation. Additionally, the photoactivated PGCZ system also presents outstanding immunomodulatory and ROS scavenging capacities, which regulate M2 polarization of macrophages and drive functional cytokine secretion, thus leading to a pro-regenerative microenvironment *in situ* with enhanced vascularization. *In vivo* experiments further demonstrated that the PGCZ platform in conjunction with mild photothermal therapeutic activity remarkably attenuated the local inflammatory cascade, initiated endogenous stem cell recruitment and neovascularization, and orchestrated the osteoblast/osteoclast balance, ultimately accelerating diabetic bone regeneration.

**Conclusions:** This work highlights the potential application of a photoactivated soft-hard combined system that provides long-term biophysical (mild photothermal stimulation) and biochemical (on-demand ion delivery) cues for accelerated healing of diabetic bone defects.

## Introduction

Physiologically, bone defect healing is a closely coordinated process that involves multiple overlapping phases: initial inflammatory and immune responses, angiogenesis, osteogenic differentiation, and bone mineralization 1. However, under hyperglycemic and hypoxic conditions, the regeneration of diabetic bone defects is often stalled in the inflammation stage due to the accumulation of excessive reactive oxygen species (ROS) and proinflammatory mediators (e.g., tumor necrosis factor-α (TNF-α) and interleukin-6 (IL-6)) secreted by inflammatory cells (neutrophils and M1 phenotype macrophages) 2. In particular, macrophages, as one of the most vital innate immune cells, are more inclined to polarize toward the proinflammatory M1 phenotype (classically-activated) under these pathological milieus, thus exacerbating the inflammatory process and further causing oxidative stress and cellular damage 3. In addition, increased levels of ROS and proinflammatory cytokines in bone defect sites can trigger a ROS-inflammation cascade cycle that further exacerbates oxidative stress damage and inhibits angiogenesis and osteoblast function 4. Even worse, long-term accumulation of proinflammatory M1 macrophages and inflammatory mediators increases the likelihood of bacterial infection, which, if not addressed, might lead to serious consequences such as osteomyelitis, aggravating the vicious inflammatory cycle 5. These extreme adverse factors may largely delay the bone healing process and lead to bone loss, which has exerted a considerable burden on individuals, society, and the economy. Although new therapeutic modalities, such as local administration of bioactive agents 6, exosomes 7, hyperbaric oxygen therapy 8 or stem cell transplantation 9, have gained widespread attention to facilitate bone healing under diabetic conditions, their benefits are rather limited and controversial. Additionally, the application of these approaches in clinical practice is associated with problems, such as initial burst release, easy inactivation of biological factors, drug-associated complications, high costs and complex manufacturing processes, and low efficacy under conditions of immune imbalance and uncontrolled ROS accumulation in individuals with diabetes 10. Considering these potential limitations, it is particularly challenging and significant to develop new therapeutic solutions to minimize these adverse effects and improve the therapeutic outcomes of diabetic bone defects with prolonged inflammation and high levels of ROS.

With the development of materiobiology, increasing evidence shows that the timely termination of the proinflammatory response by optimizing the transition of M1-to-M2 macrophage polarization (i.e., immunomodulation) can modulate the occurrence and progression of bone regeneration, including accelerated angiogenesis and mesenchymal stem cell (MSC) recruitment, and subsequent osteogenic differentiation and mineralization 11, 12. However, the treatment strategies currently available for large-scale bone defects, including autologous bone and allogeneic bone grafts, fail to address the aforementioned key points through incorporating desired functionalities and are further limited by their source, donor site morbidity, risk of infection, and host immune responses 13. Such a dilemma highlights that the rational design and development of advanced biomaterials with multiple functions, including immune regulation, infection therapy, revascularization, and bone regeneration, are imperative in the diabetic bone microenvironment. In recent decades, near-infrared (NIR) light-mediated photothermal therapy (PTT) has garnered considerable interest in bone tissue engineering, as it demonstrates a series of promising features, including low invasiveness, high spatial and temporal precision, and strong tissue penetration 14-17. In particular, mild PTT (MPTT) at an appropriate temperature of < 45 °C holds significant promise as a therapeutic approach for accelerating bone regeneration by inducing osteogenesis and angiogenesis as well as regulating the immune response 18. The potential mechanism by which NIR-triggered local hyperthermia promotes bone repair involves the upregulation of heat shock proteins (HSPs) and the alteration of the cytoskeleton and integrin signaling, which collectively boost cellular functions, vascularization, and osteogenesis 19. Excitingly, the upregulation of HSPs triggered by mild hyperthermia (~45 °C) has also been identified to evoke autoimmune modulation and block the proinflammatory cascade through the activation of the phosphatidylinositol 3-kinase (PI3K)/protein kinase B (AKT) signaling pathway and cell adhesion molecules 20. Besides, Qi et al. found that NIR-derived mild thermal stimulation could protect cells from ROS-induced oxidative damage by inhibiting the activation of the nuclear transcription factor-κB (NF-κB) signaling pathway, thereby promoting tissue regeneration under chronic inflammatory conditions 21. Motivated by these investigations, we previously designed a series of photothermal effect-reinforced multifunctional scaffolds for integrated immune regulation, vascular regeneration, bacterial elimination, and bone repair 22, 23. Nevertheless, the potential impact and mechanism of photothermal biomaterials on diabetic bone regeneration are still unclear, and we cannot completely address diabetic inflammatory microenvironment-induced complications, including high levels of ROS, inflammation, weak tissue regeneration ability, and susceptibility to bacterial infection. Furthermore, because of the complexity and heterogeneity of diabetic bone defects, a single MPTT session has a limited therapeutic effect and is often insufficient to address the multiple biological requirements of diabetic bone regeneration.

Among various photothermal materials, polydopamine (PDA), which is based on the oxidative self-polymerization of dopamine, is a promising photothermal conversion platform that can be used as a shell to improve the biocompatibility of other materials while simultaneously conferring photothermal properties. Besides, PDA modification can enhance cell adhesion, proliferation, and osteogenic differentiation and upregulate the expression of osteogenesis- and angiogenesis-related genes 24. Nevertheless, PDA has limited biological activity and cannot meet the various clinical needs for the treatment of diabetic bone defects, such as antibacterial activity, immune regulation, vascularization, and tissue repair. As a representative family member of metal-organic frameworks (MOFs), zeolite imidazoline framework-8 (ZIF-8), made of zinc ions (Zn^2+^) and 2-methylimidazole (2-MIM), has emerged as a potential candidate to promote bone regeneration due to its outstanding biocompatibility, immunomodulatory activity, osteoconductivity, and antibacterial capacity 25. Zinc, an essential trace element in the human body, has been demonstrated to promote osteogenic differentiation and matrix mineralization by activating the mitogen-activated protein kinase (MAPK) signaling pathway, which plays a pivotal role in the growth and mineralization of bone tissues 26. In addition, other studies have indicated that Zn^2+^ can induce macrophage phenotype transition from the proinflammatory M1 phenotype to the pro-healing M2 phenotype via inhibition of the NF-κB signaling pathway (an M1 phenotype-related pathway) and activation of the Janus kinase-signal transducer and activator of transcription (JAK-STAT) signaling pathway (an M2 phenotype-related pathway), which induces a favorable osteoimmunomodulatory microenvironment to enhance bone defect healing 27. Significantly, as a common broad-spectrum antibacterial agent, Zn^2+^ has also been shown to have excellent antibacterial activity against both *Staphylococcus aureus* (*S. aureus*) and *Escherichia coli* (*E. coli*), demonstrating the capacity to efficiently eliminate bacteria and prevent infection during the repair of bone defects 28. Given the above issues, we envisioned that the engineering of a ZIF-8-based nanomaterial system modified with PDA would not only integrate the features of photothermal effects but also utilize exceptional bioactivities and biodegradation products (e.g., Zn^2+^) to regulate the local immune response, promote bone regeneration, and eliminate bacteria in the diabetic pathological milieu.

However, the utilization of nanomaterials alone cannot provide the adequate mechanical cues needed for bone repair, because the reconstruction and regeneration of large bone defects still require three-dimensional (3D) biomaterial scaffolds to afford structural support to maintain the physiological and cellular processes that occur during new bone formation 29. Among various bone repair scaffolds, hydrogels feature 3D network structures similar to those of the natural extracellular matrix (ECM) and high biocompatibility and have emerged as promising candidates for bone tissue repair and regeneration 30. Despite these desirable properties, the widespread application of hydrogels in bone tissue engineering still suffers from certain shortcomings, such as limited mechanical properties, vulnerability to degradation, unsatisfactory osteoconductivity and pro-angiogenic activity, and insufficient immunomodulatory function, especially for hydrogels formed from natural biopolymers (e.g., collagen/gelatin, silk fibroin, chitosan, and hyaluronic acid) 31. Current approaches primarily focus on the incorporation of nanofillers or structural modification of hydrogels (e.g., ZIF-8-loaded hydrogels) 32, 33, but they still do not meet the mechanical and biological requirements for bone repair, especially in diabetes-related inflammatory microenvironments 34. Most recently, 3D-printed poly(ε-caprolactone) (PCL) scaffolds have been extensively studied as implanted biomaterials in bone regeneration research because of their personalized design and excellent mechanical properties 35, 36. Although these materials exhibit good mechanical properties, insufficient functionalization and weak bioactivity impede their further application in the clinic 29. Hence, it is anticipated that the rational combination of 3D-printed scaffolds with bioactive hydrogels can make full use of their advantages to attain desirable bone healing outcomes through consideration of the multiple challenges described above.

**Scheme 1:**
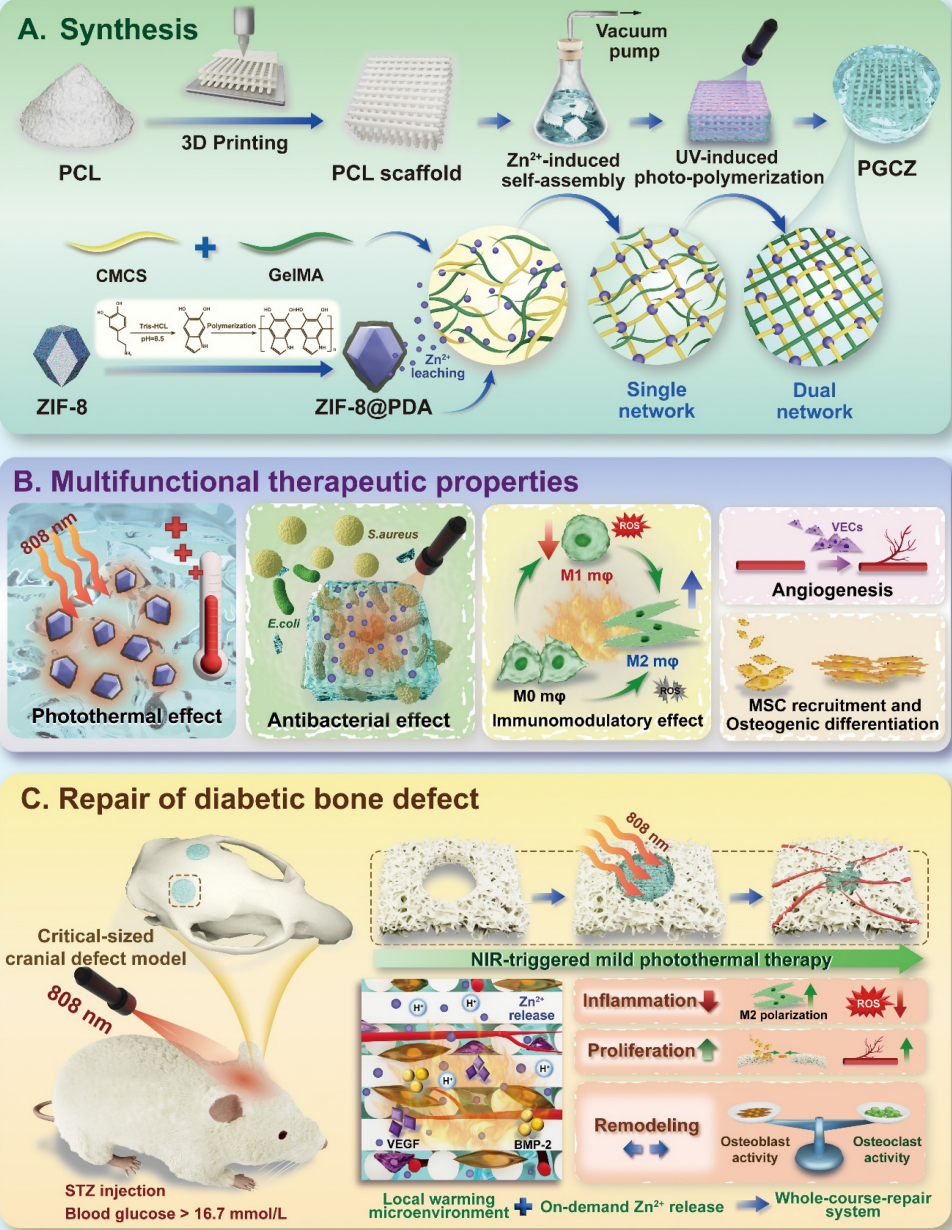
Schematic illustration of **(A)** the synthesis of the PGCZ scaffold with **(B)** multifunctional properties for **(C)** potential application in diabetic bone healing and reconstruction through programmed regulation of the regeneration process.

Herein, for the first time, a soft-hard concept to guide the design of a photoactivated scaffold therapeutic system was proposed for integrated immune regulation, ROS and bacterial elimination, vascular regeneration, and bone repair in the diabetic microenvironment. The preparation process and multifunctional therapeutic properties are shown schematically in **Scheme 1**. In this soft-hard concept system, the macroporous PCL scaffold is first fabricated by 3D printing and used as a supportive substrate (hard matrix component) for cranial defect repair. To further empower the PCL scaffold with extraordinary photothermal and therapeutic effects, the optimized double-network hydrogel (GMCS/Z) was infused into the pores of the 3D-printed PCL scaffold to form the photoactivated bone repair therapeutic system (PGCZ) for the scientific treatment of diabetic bone defects. The hydrogel network of GMCS/Z (soft matrix component) was constructed based on gelatin methacrylate (GelMA), carboxymethyl chitosan (CMCS), and PDA-modified ZIF-8 (ZIF-8@PDA) via the photo-triggered free radical polymerization of GelMA and chelation between Zn^2+^ and CMCS. In addition to acting as an efficient crosslinking and photothermal conversion agent, the ZIF-8@PDA nanoplatform could release bioactive Zn^2+^ to take effects in anti-inflammation, antibacterial, immune regulation, and bone regeneration. Benefiting from the good NIR/pH dual-responsive properties of the PGCZ system, Zn^2+^ can be released intelligently in defect lesions under the influence of the slightly acidic diabetic microenvironment and/or NIR stimulation to exert its therapeutic effect. This on-demand release behavior favors its further biological application *in vivo*. Under the combined action of NIR-triggered mild hyperthermia and sustained release of Zn^2+^, the rationally engineered multifunctional PGCZ scaffold not only demonstrated favorable biocompatibility and antibacterial activity, but also had satisfactory capacity to promote cell adhesion, proliferation, migration, angiogenesis, and osteogenic differentiation as well as induce M2 polarization of macrophages. After being applied to critical-sized cranial defects in diabetic rats, PGCZ+NIR considerably accelerated bone healing by regulating the microenvironment in terms of attenuating the inflammatory cascade, recruiting endogenous MSCs and endothelial cells, and promoting neovascularization and new bone formation. This proof-of-concept study strongly demonstrated that PGCZ+NIR possesses multifunctional properties and holds enormous potential for advancing bone tissue engineering and regenerative medicine, which could also serve as an ideal photoactivated therapeutic platform for the management of orthopedic-related complex diseases by eradicating bacterial infection, scavenging ROS, reducing inflammation via macrophage M2 polarization, and promoting angiogenesis and bone regeneration.

## Results and Discussion

### Preparation and characterization of GelMA, ZIF-8@PDA, and hybrid hydrogels

**Figure 1:**
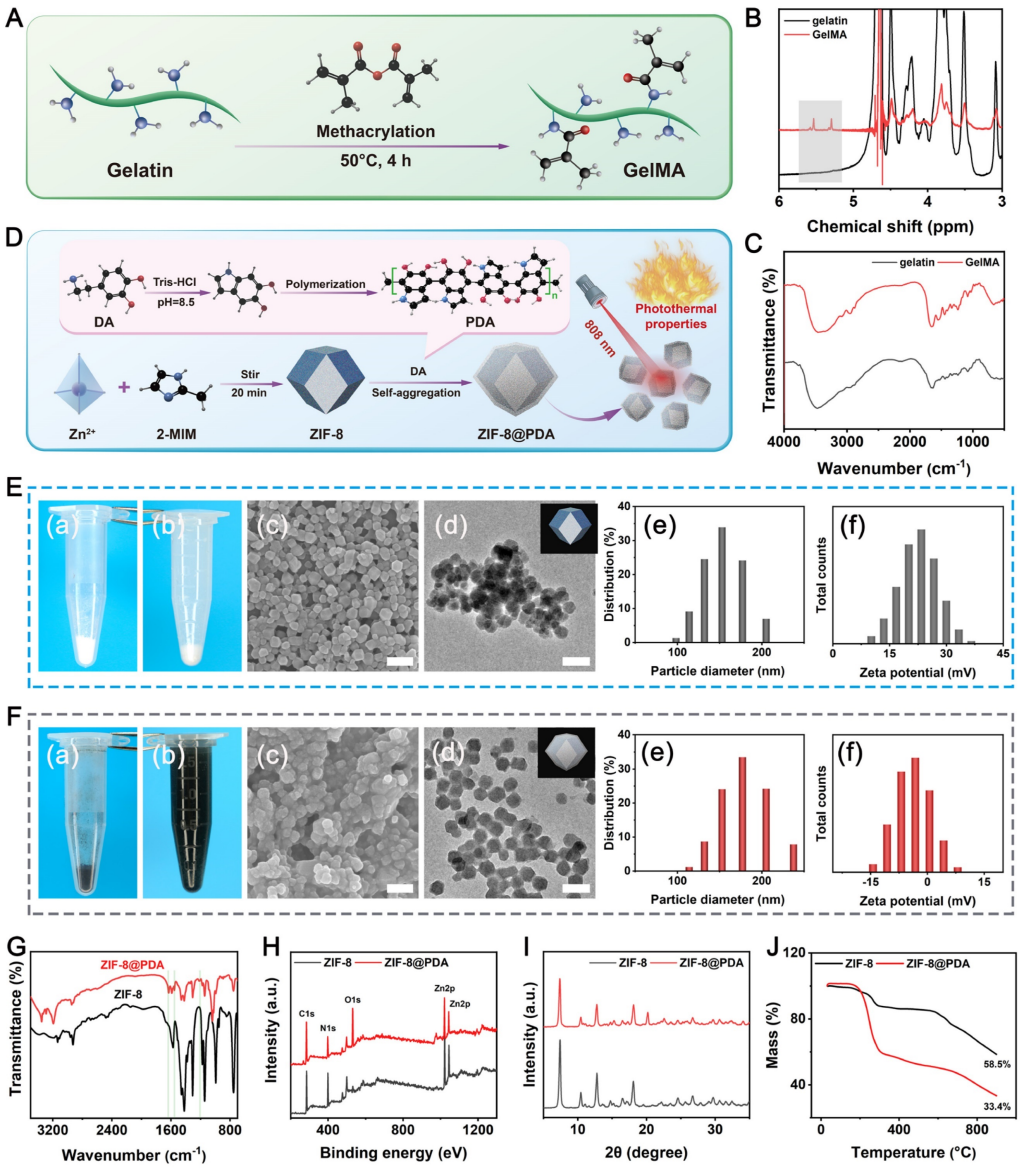
** Preparation and characterization of GelMA and ZIF-8@PDA nanoparticles. (A)** Schematic illustration of GelMA synthesis. **(B)** ^1^H NMR and **(C)** FTIR of gelatin and GelMA. **(D)** Schematic illustration of ZIF-8@PDA synthesis and its photothermal properties. **(E)** Morphological and basic characteristics of the ZIF-8 nanoparticles: Photographs of the nanoparticles (a) before and (b) after dispersion in PBS; (c) SEM image. Scale bar: 300 nm; (d) TEM image. Scale bar: 300 nm; (e) Particle size distribution; (f) Zeta potential. **(F)** Morphological and basic characteristics of the ZIF-8@PDA nanoparticles: Photographs of the nanoparticles (a) before and (b) after dispersion in PBS; (c) SEM image. Scale bar: 300 nm; (d) TEM image. Scale bar: 300 nm; (e) Particle size distribution; (f) Zeta potential. **(G)** FTIR spectra, **(H)** XPS analysis, **(I)** XRD spectra, and **(J)** TG diagrams of pure ZIF-8 and ZIF-8@PDA nanoparticles.

In this work, a versatile hydrogel-scaffold platform (PGCZ) with well-integrated functionalities and biological performance was delicately designed and acted as a mild photothermal stimulator to simultaneously assist in immunomodulation, ROS scavenging, bacterial killing, osteogenesis, and angiogenesis, which might be beneficial for bone regeneration under diabetic inflammatory conditions. The design concept of this soft-hard combined PGCZ scaffold system and its synthesis process are presented in **Scheme 1**, including the preparation of the hydrogel precursor and 3D-printed scaffold and the expected biological functions and bone healing process. This preparation process was inspired by the formation of a structure in which steel bars are wrapped with concrete. Specifically, a ZIF-8@PDA-loaded GelMA/CMCS composite hydrogel (GMCS/Z) was first fabricated as a soft substrate to mimic the chemical composition, spatial structure, and biological function of the natural ECM. Here, both GelMA and CMCS hydrogels were chosen as the primary constituents to fabricate the functional soft matrix in our scaffold system because of their high biocompatibility, chemical similarity to natural bone ECM, and presence of cell-responsive arginine-glycine-aspartic acid (RGD) peptides 37. The modification diagram of gelatin is displayed in **Figure 1A**, which suggested that the methacrylate group was grafted into the backbone of gelatin. To verify the successful synthesis of GelMA, proton-1 nuclear magnetic resonance (^1^H-NMR) and Fourier transform infrared (FTIR) spectra were obtained, as shown in **Figure 1B-C**. New resonance peaks at 5.3 and 5.5 ppm in the ^1^H-NMR spectra confirmed the presence of methyl groups (-CH=CH_2_), indicating the successful introduction of double bonds into the gelatin. The substitution degree of the methacrylate group on gelatin was calculated to be 40.98%. The FTIR spectra showed that the characteristic peaks of both amide I (C=O stretching) and amide II (N-H bending) shifted to higher wavenumbers after chemical modification, indicating that gelatin reacted with methacrylic anhydride to form double bond-modified gelatin (GelMA). Our results were also in agreement with those of previous studies 14, demonstrating the successful conjugation of reactive methacrylate groups to gelatin.

Subsequently, we prepared the functionalized photothermal nanosystem ZIF-8@PDA via bioinspired dopamine chemistry 38, in which ZIF-8 was obtained by the coordination of 2-methylimidazole (2-MIM) with zinc ions (Zn^2+^) followed by *in situ* self-polymerization of dopamine under alkaline conditions (**Figure 1D**). The resulting ZIF-8@PDA nanoparticles were used as crosslinkers to react with the CMCS polymer, by which a 3D network of the GMCS/Z hydrogel was generated based on chelation between Zn^2+^ and CMCS as well as photo-crosslinking of GelMA (**Scheme 1**).

In addition, after PDA modification, the surfaces of the ZIF-8@PDA nanoparticles possessed abundant phenolic hydroxyl groups, facilitating their incorporation into the GMCS matrix to produce GMCS/Z composite hydrogels. The gross appearance of the as-synthesized particles was evaluated by direct visualization, and it could be clearly observed that the color of the powder changed from lightly white (**Figure 1E(a)**) to dark black (**Figure 1F(a)**) after PDA modification. One possible reason should be ascribed to the self-polymerization of dopamine under alkaline conditions, which was oxidized by oxygen to quinone and immediately reacted with catechol to generate a black PDA layer on the nanoparticle 39. Notably, it could be observed that the ZIF-8@PDA nanoparticles were uniformly dispersed in phosphate-buffered saline (PBS) solution (**Figure 1F(b)**), while the ZIF-8 nanoparticles all precipitated after 2 h (**Figure 1E(b)**). The better water dispersibility of the ZIF-8@PDA nanoparticles may be because of the formation of a shell shield by tethered organic molecules in PDA, which reduces interparticle electrostatic interactions to prevent the aggregation of ZIF-8@PDA.

The morphology and distribution of the as-synthesized nanoparticles were preliminarily observed using scanning electron microscopy (SEM) and transmission electron microscopy (TEM), as shown in **Figure 1E(c-d)-F(c-d)**. The results confirmed the production of spherical ZIF-8 and ZIF-8@PDA nanoparticles with nanometer-scale sizes after sonication. Moreover, agglomerated ZIF-8 clusters were also observed, in contrast to the uniformly dispersed ZIF-8@PDA nanoparticles, which was in accordance with the gross observation. It has been reported that PDA modification can reduce particle agglomeration and induce more even particle distribution 40. High-magnification TEM images showed that ZIF-8 had a relatively smooth surface, whereas ZIF-8@PDA had a core-shell structure (**Figure S1**). The opaque shell was supposed to be PDA layer deposited on the surface of ZIF-8 with a thickness of approximately 11 nm, demonstrating the strong binding between ZIF-8 and PDA. Energy dispersive spectroscopy (EDS) elemental mapping revealed that C, N, O, and Zn coexist and are distributed uniformly (**Figure S2**), further confirming the successful encapsulation of PDA. From dynamic light scattering (DLS) analysis, the average particle diameters of ZIF-8 and ZIF-8@PDA were 152.9 nm and 177.1 nm, with polydispersity indices (PDIs) of 0.997 and 0.32, respectively (**Figure 1E(e)-F(e)**). These DLS analysis data were basically consistent with the SEM and TEM results and further supported that ZIF-8@PDA is much more dispersed and more stable than ZIF-8 in aqueous solution, indicating a narrow size dispersion. Additionally, the ζ-potentials of ZIF-8 and ZIF-8@PDA were 22.6 and -4.62 mV, respectively (**Figure 1E(f)-F(f)**). The potential reduction in ZIF-8@PDA was primarily attributed to the exposure of phenolic hydroxyl groups on the surface of PDA, consistent with previously reported studies 22. Notably, a more negative zeta potential is beneficial for the dispersibility of nanoparticles 41. Modification of the particle surface with PDA effectively prevented agglomeration induced by the high surface energy, thus improving the stability and dispersibility of the nanomaterials. These data imply that ZIF-8@PDA is much more dispersed and more stable than ZIF-8 in aqueous solution, which agrees well with the SEM and TEM results. Subsequently, the chemical properties of bare ZIF-8 and ZIF-8@PDA were further determined via FTIR analysis. After mussel-inspired modification, ZIF-8@PDA exhibited three new peaks at 1218, 1553 and 1630 cm^-1^, which were associated with amide III, amide II, and amide I of PDA, respectively (**Figure 1G**), demonstrating the successful polymerization of dopamine in the system. For Raman spectroscopy, two broad peaks situated at 1591 and 1380 cm^-1^ appeared in the spectrum of ZIF-8@PDA (**Figure S3**), mainly due to the stretching and deformation of the aromatic ring from PDA, which is consistent with previously reported data 42. X-ray photoelectron spectroscopy (XPS) was used to verify the molecular structure of ZIF-8@PDA (**Figure 1H**). Compared with those of raw ZIF-8, lower N 1s peaks and higher O 1s peaks were observed in ZIF-8@PDA, which might be caused by the low N content and high O content in PDA (**Figure S4**), confirming that phenolic hydroxyl groups were grafted onto the ZIF-8 surface. Furthermore, the X-ray diffraction (XRD) pattern showed that ZIF-8@PDA still had strong diffraction peak intensities at the (011), (002), (112), (022), (013), and (222) crystal faces (**Figure 1I**), which was consistent with the results for standard samples 38, indicating that the modification did not destroy its original crystalline structure. Notably, the intensity of all these diffraction peaks decreased after the modification, which might be attributed to the masking effect of the PDA layer. Thermogravimetric (TG) analysis revealed that the remaining weight percentages at 900 °C for ZIF-8 and ZIF-8@PDA were 58.5% and 33.3%, respectively (**Figure 1J**), reflecting the successful deposition of PDA on the nanoparticles.

The NIR-triggered photothermal conversion efficiency of PDA endows the ZIF-8 nanosystem with excellent photothermal therapeutic activity to meet the basic requirements of photothermal biomaterials. Before incorporation into the hydrogel, the photothermal performance of ZIF-8@PDA was investigated using 1.0 W/cm^2^ of 808 nm NIR irradiation. The photothermal heating curves of the PBS, ZIF-8 and ZIF-8@PDA aqueous solutions were recorded after NIR irradiation for 5 min, as shown in **Figure S5A**. As expected, the pure PBS and ZIF-8 solutions showed no obvious temperature variation, as measured by an infrared thermogram under NIR irradiation. In comparison, the temperature of the ZIF-8@PDA aqueous solution continuously increased and eventually reached an equilibrium temperature of 55.6 °C within 5 min (**Figure S5B**). The enhanced photothermal activity of ZIF-8@PDA may be reasonably attributed to the strong NIR absorption induced by the π-π* transition of the polymeric backbone of the benzenoid ring originating from the PDA structure 20. Moreover, ZIF-8@PDA displayed high photothermal stability (**Figure S5C**), which could be switched on/off by NIR laser loading/unloading. The increase in temperature was fully retained after five on/off laser cycles, indicating the high stability of the PDA-functionalized ZIF-8 nanoparticles. Strong absorption from the UV to the NIR region is a prerequisite for photothermal conversion. Therefore, the photothermal performance of the ZIF-8@PDA nanoparticles was further evaluated using UV-vis spectroscopy. It could be observed that the absorption band across the UV-vis region of ZIF-8@PDA was remarkably enhanced compared with that of pure PBS and the ZIF-8 aqueous solution (**Figure S6**), showing a broader and stronger absorption band. Consequently, these fascinating characteristics of ZIF-8@PDA enable it to be used as a potential NIR-controlled photothermal conversion agent for bone tissue engineering applications.

**Figure 2:**
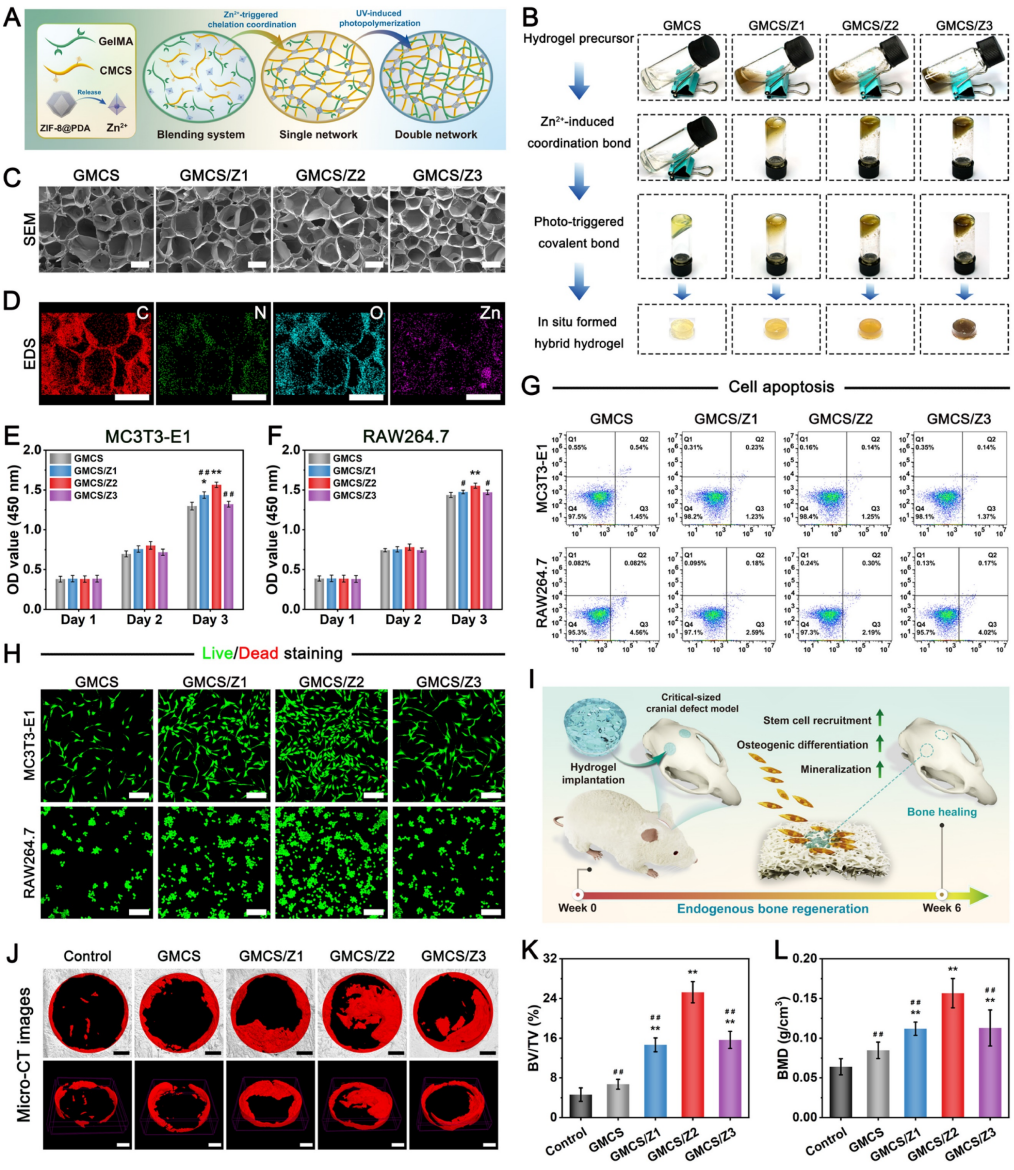
** Fabrication, characterization, and bioactivity of the prepared GMCS/Z hydrogels. (A)** Schematic illustration of the dual-crosslinked polymer network that forms the hybrid hydrogel. **(B)** Macroscopic view of various hydrogel precursor solutions before and after gelation. **(C)** SEM images and **(D)** EDS elemental mapping images of different hydrogels after lyophilization. Scale bar: 200 μm. Cytotoxicity of the GMCS/Z hydrogels in **(E)** MC3T3-E1 cells and **(F)** RAW264.7 cells determined by CCK-8 analysis. **(G)** Apoptosis detection in MC3T3-E1 cells and RAW264.7 cells after different treatments. **(H)** Live/dead staining images of MC3T3-E1 cells and RAW264.7 cells after different treatments. Scale bar: 100 μm. **(I)** Schematic diagram of hydrogel implantation for the treatment of critical-sized cranial defects. **(J)** Micro-CT images of new bone formation in the defect regions at 6 weeks after implantation. Scale bar: 1 mm. Quantitative analysis of **(K)** BV/TV and **(L)** BMD based on micro-CT. Data are presented as the mean ± SD (n = 3). *P < 0.05 and **P. < 0.01 indicate significant differences compared with the control group. ^#^P < 0.05 and ^# #^P < 0.01 indicate significant differences compared with the GMCS/Z2 group.

To maintain the biological activity of ZIF-8@PDA and simultaneously engineer a soft substrate with biomimetic ECM-like porous structure, the ZIF-8@PDA nanoparticles were mixed with GelMA and CMCS, which then formed GelMA/CMCS/ZIF-8@PDA (GMCS/Z) crosslinking system, as a result of the photo-polymerization of GelMA and ionic crosslinking between Zn^2+^ and CMCS (**Figure 2A**). In the constructed hybrid GMCS/Z hydrogel system, the C=C bonds in the methacrylate group can undergo free radical polymerization to form C-C bonds in the presence of the photoinitiator lithium phenyl (2,4,6-trimethylbenzoyl) phosphinate (LAP). Furthermore, the addition of ZIF-8@PDA contributed to the formation of dynamic physical crosslinking via chelation between the functional groups (-COOH, -NH_2_, and -OH) of the CMCS chains and Zn^2+^. The phase transitions of the GMCS/Z hydrogels before and after crosslinking were observed with digital images. As shown in **Figure 2B**, the color of the GMCS/Z hydrogel gradually changed from translucent to dark brown with increasing ZIF-8@PDA concentration after gelation, suggesting that ZIF-8@PDA was successfully encapsulated in the hydrogel matrix.

To determine the optimal composition ratio of this hydrogel for use as a soft substrate in our system for bone regeneration, a series of characterization experiments and biological experiments were conducted. The microscopic structure of the hydrogel was characterized by SEM, as shown in **Figure 2C**. After lyophilization, all the hydrogels exhibited uniform and highly interconnected 3D porous microstructures. The pore structure parameters did not differ significantly between groups with pore sizes ranging from 200-250 μm. This 3D interconnected network with desirable porosity (~85%) was highly similar to that of natural bone ECM (**Figure S7**), which could not only favor cell infiltration, vascular formation and tissue growth but also provide active adsorption sites for bioactive molecules as well as improved nutrient and metabolic waste transportation 43. From the SEM images at high magnification, it was demonstrated that a large amount of nanosized ZIF-8@PDA particles with spherical structures were homogeneously embedded throughout the hydrogel matrix (**Figure S8**), which exhibited a micro-nano hierarchical structure. The EDS elemental mapping analysis further confirmed this observation, as evidenced by the presence of C, O, N, and Zn element (**Figure 2D**). The introduction of ZIF-8@PDA nanoparticles showed no obvious influence on pore size, while the roughness of the pore wall visibly increased compared with that of the GMCS hydrogel, which was shown to be beneficial for cell adhesion and migration 44. Subsequently, the crystalline structure of the GMCS/Z hydrogel was investigated by XRD. As depicted in **Figure S9**, with increasing ZIF-8@PDA concentration, several characteristic peaks at 2θ = 7.4°, 10.6°, 12.8°, and 18.1°, corresponding to the (011), (022), (112), and (222) diffraction planes, gradually appeared, indicating that ZIF-8@PDA was successfully incorporated into the GMCS hydrogels.

After introducing the ZIF-8@PDA nanoparticles, Zn^2+^-triggered chelation coordination and UV-induced photo-polymerization could synergistically promote crosslinking of the hydrogel network, which was likely to improve the mechanical performance. Thus, the mechanical properties of the as-prepared hydrogels were investigated systematically. **Figure S10A** depicts the compressive stress-strain curve of the hybrid hydrogels. Our results demonstrated better mechanical performance of the GMCS/Z hydrogels with the incorporation of ZIF-8@PDA due to secondary network formation. As the concentration of ZIF-8@PDA increased, the GMCS/Z exhibited an enhanced compressive strength, indicating that the introduction of a secondary crosslinking network by loading ZIF-8@PDA could remarkably reinforce its mechanical strength. The introduction of ZIF-8@PDA nanoparticles with abundant phenolic hydroxyl groups could also provide additional hydrogen bonding sites for GelMA and CMCS, causing competitive binding in the hydrogel network. Previous studies have shown that mussel-inspired molecules, i.e., PDA, could act as bridge to anchor polymer chains to strengthen the crosslinked network 40. Beyond that, the uniform distribution of ZIF-8@PDA strengthened the hydrogel network via a nanoenhancement effect.

However, the high concentration of ZIF-8@PDA (> 5%) may destroy the chemical (photo-triggered free radical polymerization) or physical (chelation coordination and hydrogen bonds) interactions between the GelMA chains themselves or between the ZIF-8@PDA and CMCS chains, and rearrange the chains to a certain extent, which ultimately leads to a decrease in the mechanical performance (**Figure S10B**). According to the mechanical test, the addition of ZIF-8@PDA at a moderate concentration led to a dramatic improvement in the compressive strength of the hydrogel, while an increase in the ZIF-8@PDA content (>5%) could undermine the mechanical properties. The same phenomenon can also be observed in the rheological experiment, as displayed in **Figure S11**. As the oscillation frequency increased, all the hydrogels exhibited similar nonlinear rheological behavior. The energy storage modulus (G') of all the hydrogels was higher than the loss modulus (G''), suggesting good mechanical performance of the hydrogel. Simultaneously, the increases in the G′ and G″ values tended to increase with increasing ZIF-8@PDA concentration in the composite hydrogels, suggesting that additional crosslinking occurred to stabilize the network after the introduction of ZIF-8@PDA. More importantly, the values of G' and the corresponding G'' significantly increased in the GMCS/Z2 group in contrast to those in all the other groups, indicating that the GMCS/Z2 hydrogel had better mechanical properties than did the GMCS, GMCS/Z1, and GMCS/Z3 hydrogels, consistent with the compressive results. In summary, ZIF-8@PDA incorporated within the prepared hydrogel provided abundant adhesion and chemical reaction sites that improved the physicochemical properties of the resultant materials and facilitated interfacial integration between the GelMA and CMSC polymer chains.

### Optimization of the GMCS/Z hydrogel precursor

Next, the GMCS/Z hydrogels were optimized by both *in vitro* and *in vivo* evaluations. Osteoblasts and macrophages play critical roles in osteogenesis and immunomodulation, respectively, so the cytotoxicity of different hydrogel samples was tested on an osteoblast cell line (MC3T3-E1 cells) and a macrophage line (RAW264.7 cells), and the corresponding results are displayed in **Figure 2E-F**. According to the CCK-8 assay, the cell viability of the four hydrogel groups increased gradually with prolonged incubation time, indicating that the loading of ZIF-8@PDA had no obvious toxic effects. After 3 days of co-culture, a substantial increase in the number of both MC3T3-E1 and RAW264.7 cells was observed in the GMCS/Z2 hydrogel group, illustrating its considerably stimulatory effect on cell proliferation. Similarly, the quantitative flow cytometric assay results demonstrated that all the hydrogels had negligible cytotoxic effects on both the MC3T3-E1 cells and the RAW264.7 cells. After incubation for 3 days, both cell lines maintained high viability (> 95%), demonstrating reliable biosafety and proliferation ability after introducing ZIF-8@PDA (**Figure 2G**). To provide further evidence, a live/dead staining assay was used to investigate cell proliferation on day 3, and similar trends were observed (**Figure 2H**). In particular, compared with those in all the other groups, the number of live cells labeled with green fluorescence in the GMCS/Z2 group was significantly increased (**Figure S12**), demonstrating that GMCS/Z2 possessed the combined properties of optimal cytocompatibility and proliferation-promoting ability. The selection of hydrogel composition is of utmost importance in promoting cell growth and achieving desired tissue regeneration outcomes 45. In our study, both GelMA and CMCS were found to be derivatives of biomolecules (gelatin and chitosan), which ensures the cytocompatibility and biosafety of the hydrogels. Furthermore, the presence of abundant phenolic hydroxyl groups and bioactive Zn^2+^ in ZIF-8@PDA played a beneficial role in cell survival and proliferation, leading to the excellent biological effect of the GMCS/Z hydrogels. These results are in good accordance with previous cell viability assay data and further confirm the good biocompatibility of the fabricated composite hydrogels 33. Taken together, the results showed that the prepared GMCS/Z hydrogels had no cytotoxic effect and could create a favorable regenerative microenvironment for supporting cell growth and proliferation. Furthermore, the effect of the GMCS/Z2 hydrogel group was particularly significant, providing basic conditions for further application.

Next, we evaluated the therapeutic effects of the composite hydrogels on critical-sized calvarial defects in rats according to the experimental plan shown in **Figure 2I**. During the experimental period, bilateral calvarial defects were created using a trephine (5 mm in diameter). The sterilized hydrogels were then implanted into the defects to simulate clinical bone repair conditions. Following 6 weeks of implantation, rat cranial samples were collected for micro-CT, histological examination, and immunohistochemical staining analyses. As expected, the blank control group was filled with fibrous tissue, and almost no new bone formation was observed after implantation, indicating poor bone regeneration ability. Conversely, partial bone tissue was found in the defect region of the GMCS group, and a large amount of newly formed bone tissue was detected in all the GMCS/Z groups, especially in the GMCS/Z2 group, as evidenced by the 3D reconstructed micro-CT images (**Figure 2J**). Morphogenetic analysis of regenerated bone also confirmed that the percentage of regenerated bone volume and density in the GMCS/Z2 hydrogel-treated group was significantly higher than that in the other groups (**Figure 2K-L**), preliminarily confirming the prominent osteogenic ability of the GMCS/Z2 hydrogel.

Both hematoxylin and eosin (H&E) and Masson's trichrome (MST) staining showed that a greater amount of regenerated lamellar bone tissue was found in the GMCS/Z hydrogel groups, among which the GMCS/Z2 group had the optimal promotion effect on bone formation (**Figure S13A**). Additionally, the hydrogels were partially fused with the surrounding tissue, suggesting good compatibility and osteointegration *in vivo*. Immunohistochemical staining further demonstrated that the GMCS/Z2 group presented higher positive expression of CD90, Runx2, and OPN than did all the other groups (**Figure S13A-D**), collectively implying elevated osteogenesis and biomineralization. Notably, there was obvious enrichment of MSCs in the bone defect areas, which further supported the ability of the GMCS/Z2 hydrogel to facilitate new bone formation. The aforementioned *in vitro* and *in vivo* investigations indicated that the as-prepared GMCS/Z2 hydrogel had a substantial promoting effect on cell proliferation, survival, and growth, as well as efficiently accelerated the regeneration of damaged bone tissue.

### Preparation and characterization of the hydrogel-functionalized scaffolds

**Figure 3:**
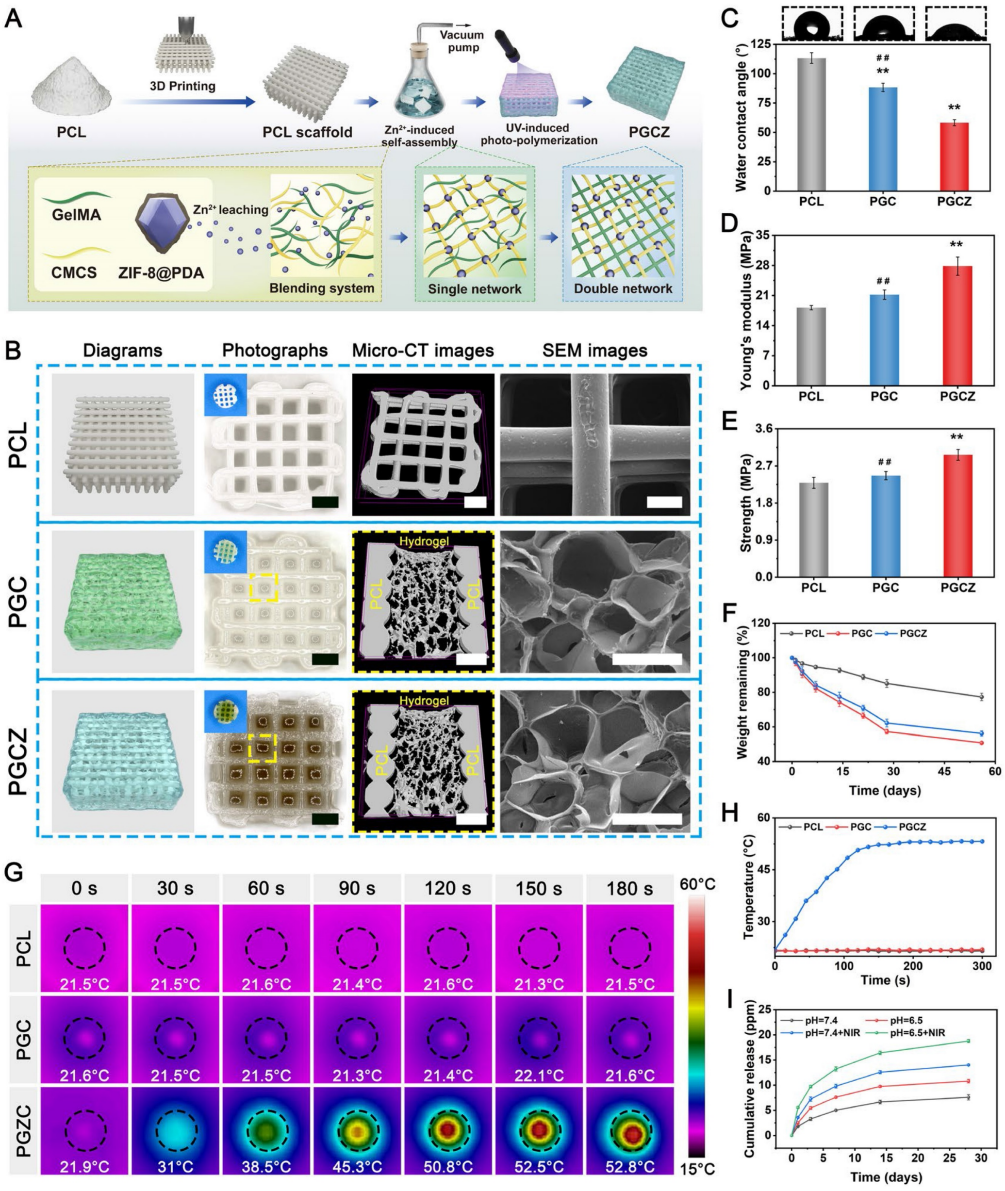
** Preparation and characterization of photoactivated PGCZ hybrid scaffolds. (A)** Schematic diagram of the PGCZ hybrid scaffold fabrication process. **(B)** Schematic and morphology of PCL, PGC, and PGCZ hybrid scaffolds. Scale bar: 1 mm (optical images), 1 mm (micro-CT images in PCL), 350 μm (micro-CT images in PGC and PGCZ), and 200 μm (SEM images). **(C)** Water contact angles of the various scaffolds. **(D)** Young's modulus, and **(E)** compressive strength of the various scaffolds. **(F)** Degradation curves of the various scaffolds. **(G)** Infrared thermal images and **(H)** temperature curves of the various scaffolds under NIR laser radiation (808 nm, 1 W/cm^2^). **(I)** Release profiles of Zn^2+^ from the PGCZ scaffold with or without intermittent NIR irradiation (808 nm, 1 W/cm^2^) at different pH levels. Data are presented as the mean ± SD (n = 3). *P < 0.05 and **P < 0.01 indicate significant differences compared with the PCL group. ^#^P < 0.05 and ^# #^P < 0.01 indicate significant differences compared with the PGCZ group.

Taking the outstanding biological activities (e.g., *in vitro* cell viability and *in vivo* bone formation) and mechanical strength into consideration, GMCS/Z2 was optimal and was selected for follow-up research. To realize the soft-hard concept, the prepared hydrogel precursor solutions were introduced into macroporous PCL scaffolds to obtain PGC and PGCZ scaffolds by combining the 3D printing technique with the infiltration coating process (**Figure 3A**). With the advancement of tissue engineering, 3D printing has become a powerful technology for manufacturing bone repair materials in the clinic; however, the majority of 3D-printed scaffolds are assembled from solid struts, which limits the delivery of nutrients and oxygen, and often results in delayed tissue formation at the center of defects 46. Moreover, pure 3D-printed scaffolds usually lack robust biofunctions to meet varied regeneration applications, especially in the diabetic pathological microenvironment, where the biofunctions are disrupted by conditions such as bacterial infection, high oxidative stress, excessive inflammation, and damage to osteoblast function and blood vessel networks 47. In view of the structural and functional characteristics of the GMCS/Z2 hydrogel mentioned above, we speculate that the combination of 3D printing and hydrogel impregnation may synergistically induce bone and vascular formation for accelerated bone healing. In the macroscopic view and SEM images of the 3D-printed scaffolds, pure PCL showed ordered and uniformly arranged interconnected macropores, which allowed vacuum impregnation of the hydrogel precursor solution. **Figure 3B** confirmed the penetration of the hydrogel phase in the 3D porous PCL framework, leading to the formation of a hybrid hydrogel scaffold after gelation. To create suitable scaffolds for *in vitro* biological studies and *in vivo* animal implantation, round 3D-scaffold specimens were further punched out using a biopsy punch 5 mm in diameter (**inserts of Figure 3B**). Besides, unlike white PCL and transparent PGC scaffolds, the PGCZ scaffold showed a light brown color due to the inclusion of ZIF-8@PDA, further revealing the successful introduction of the GMCS/Z hydrogel. Moreover, elemental mapping images showed that Zn element was uniformly distributed throughout the whole PGCZ scaffold (**Figure S14**), suggesting successful impregnation of the GMCS/Z hydrogel. Micro-CT analysis was further carried out to assess the interfacial reaction between the scaffold and the hydrogel after lyophilization. As illustrated in **Figure 3B**, both the PGC and PGCZ scaffolds feature a biphasic structure composed of orthogonally arranged polymeric fibers and porous hydrogel matrices within the inner pores, which resemble the anisotropic porous structure of collagen fibril arrays in native bone 29. Besides, 3D micro-CT reconstruction images showed that the PGCZ hydrogel bonded tightly to the PCL scaffold (**Figure S15**), showing a strong interfacial adhesion effect. This might be because ZIF-8@PDA contains abundant catechol groups, which can form strong covalent and non-covalent bonds with various organic and inorganic substrates 48. In contrast, a partial detachment trend was observed between the GMCS hydrogel and the PCL scaffold, indicating poor integration. Similar findings were also obtained from the cross-sectional SEM images, in which the surfaces of PGC and PGCZ were covered by a porous hydrogel layer, further evidencing the uniform infiltration of the hydrogel precursor into the macropores of the PCL scaffolds.

The basic physical properties of the composite scaffolds were also characterized. As shown in **Figure S16A**, the pore size of the PCL scaffold was 699.4 ± 15.7 μm, while the micropore sizes of PGC and PGCZ were 232.4 ± 35.2 μm and 228.8 ± 32.9 μm, respectively. Simultaneously, we measured the porosities of the PCL, PGC and PGCZ scaffolds were 79.3 ± 2.8%, 73.1 ± 0.6%, and 70.8 ± 1.4%, respectively (**Figure S16B**), which are within the range of those of cancellous bone (50%-95%) 46. Multiple studies have reported that optimized biomaterial scaffolds with pore sizes ranging from 100 μm to 500 μm and porosities greater than 50% are beneficial for osteogenesis-related cell adhesion, proliferation, ECM production, and vascularization during the bone regeneration process 49, which further validates PGCZ as a good candidate for bone regeneration applications. Except for desirable 3D porous structures, the surface hydrophilicity of biomaterials also plays a pivotal role in the early adhesion and differentiation of osteoblasts 50. Water contact angle measurements showed that the introduction of the GMCS/Z hydrogel greatly enhanced the hydrophilicity of the PCL substrate surface (**Figure 3C**), potentially due to the exceptional hydrophilic nature of GelMA and CMCS, as well as the presence of numerous hydrophilic phenolic hydroxyl groups from ZIF-8@PDA 48. The improved hydrophilicity was beneficial for facilitating physical interactions between the scaffold material and host cells, manipulating cellular functions (e.g., cell adhesion and proliferation), and ultimately facilitating tissue regeneration and integration. Because the PCL polymer is bioinert, a well-optimized bioactive hydrogel solution was introduced into the 3D-printed PCL scaffolds to form a ZIF-8@PDA-mediated PGCZ scaffold for better surface biofunctionalization, which could support the integration of multiple functional properties and extend its application in bone tissue engineering.

Biomaterial scaffolds with long-term structural stability and sufficient mechanical support are crucial for bone healing and remodeling. Therefore, the mechanical properties of the scaffolds were tested, as shown in **Figure 3D-E**. The compressive modulus of the PGCZ scaffold (27.8 ± 2.1 MPa) was higher than that of the PCL (18.2 ± 0.5 MPa) and PGC (21.2 ± 1.1 MPa) scaffolds. The compressive strength of the PGCZ scaffold (2.9 ± 0.1 MPa) was higher than that of the PCL (2.3 ± 0.1 MPa) and PGC (2.5 ± 0.1 MPa) scaffolds, which was more favorable for long-term bone repair. Benefiting from the strong interfacial integration between the hydrogel and PCL framework network, the loading of GMCS/Z2 could greatly enhance the mechanical performance of the PCL scaffolds. This design concept mimics the reinforced concrete structures used in house construction, resulting in improved mechanical properties. Furthermore, the improved compressive strength of the PGCZ scaffold was within the same range as that of human cancellous bone (2-12 MPa) 46, 51, which suggested that the resulting PGCZ scaffold was sufficient for maintaining an integrated 3D structure and providing basic mechanical support for bone regeneration. These factors are crucial for the overall functionality of the scaffold and its therapeutic effect on bone healing under diabetic conditions.

Biodegradation plays an important role in maintaining tissue regeneration space and achieving effective cell ingrowth and blood vessel formation. Therefore, the *in vitro* degradation kinetics of the composite hydrogels were studied in 0.05% collagenase-containing PBS solution at 37 °C (with an enzyme activity of approximately 100 U/mL). The *in vitro* degradation curves demonstrated that the pristine PCL scaffold exhibited slower degradation than did PGC or PGCZ and maintained ≈78% weight retention after 58 days in a hybrid degradation solution (**Figure 3F**). Moreover, the *in vitro* degradation rate of the PGCZ scaffold at each time point was lower than that of the PGC scaffold, which might be associated with the enhancement of internal crosslinking of the hydrogel through the interaction of ZIF-8@PDA with GelMA and CMCS. Next, we assessed the scaffold-hydrogel interface stability after 28 days of degradation. The interface morphology of the scaffolds was examined using SEM, and there was a significant difference in surface morphology among the three kinds of scaffolds (**Figure S17**). It could be observed that polymer degradation almost always occurred in parts of the PGC hydrogel matrix, as evidenced by the presence of cracks and brittle fracture sections between the PCL and GMCS hydrogels. In contrast, we found that the PCL layer and GMCS/Z layer were closely integrated without obvious dissociation, which might be attributed to the outstanding adhesiveness of PDA 48. The strong interfacial bonding between PCL and crosslinked GMCS/Z hydrogel networks would be able to maintain long-term mechanical stability for structural support after *in vivo* implantation during the bone generation process. The introduction of ZIF-8@PDA provides abundant adhesion and chemical reaction sites for subsequent interface integration, which allows covalent bonding between the hydrogel coating and the PCL substrate. Importantly, the PGCZ hydrogel phase formed tight contacts with the PCL framework without obvious interface separation, which was effective in integrating multiple functionalities into one synergistic therapeutic platform.

To form a stable biological combination between the implant and the bone, i.e., osteointegration, ideal tissue engineering materials should possess excellent bioactivity and biomineralization capacity 52. It is well acknowledged that natural polymers (e.g., chitosan, gelatin, silk, and cellulose) are good compound templates for biomineralization, and catechol group-rich materials could help accelerate biomineralization, which could facilitate bone regeneration and osteointegration 53. In the present study, the composite scaffolds were soaked in 10 × simulated body fluid (SBF) solution for 24 h and then taken out for SEM characterization. As shown in **Figure S18A**, the surfaces of the PGC and PGCZ scaffolds (especially the latter) were covered with a large number of newly formed mineral crystals with nanoflake-like structures, but for pristine PCL scaffolds that were not hydrogel-modified, hardly any deposition of mineral particles could be observed on the surface. Meanwhile, some scattered salt crystals appeared around the agglomerated calcium minerals after freeze-drying, as previously reported 39. After magnification, the homogeneous precipitates of lamellar mineral crystals spontaneously assembled into a continuous and stable mineralized layer (**Figure S18B**), indicating enhanced biomineralization. The enhanced mineralization occurred because both GMCS and ZIF-8@PDA can provide additional nucleation sites for calcium deposition and matrix mineralization, which may be beneficial for guiding bone regeneration. To identify the composition of the mineral crystals, FTIR and XRD analyses were performed, and the results are illustrated in **Figure S18C-D**. The results of FTIR spectra showed that the peaks at 603 and 566 cm^-1^ are characteristic stretching and deformation vibrations of PO_4_^3-^ groups (**Figure S18C**), manifesting that the deposited mineral layer mainly consisted of hydroxyapatite (Ca_10_(PO_4_)_6_(OH)_2_). Particularly, the characteristic peak at 962 cm^-1^ is a representative indication of hydroxyapatite. Similarly, the XRD results indicated that PGC had three obvious diffraction peaks at 2θ = 32.1°, 41.1°, and 49.7° corresponding to the (211), (310) and (213) crystalline planes of hydroxyapatite crystals, respectively, and that the relative intensity increased with the inclusion of ZIF-8@PDA (**Figure S18D**), which matched the results of FTIR analysis. Simultaneously, the two major peaks at 2θ = 22.1° and 24.5° originated from the crystallinity of PCL and were present in all the spectra. As the major inorganic component in natural bone tissue, hydroxyapatite has been widely used to promote bone regeneration owing to its ability to strongly enhance osteogenic differentiation and osseointegration 54. Under physiological conditions, the bioactive hydroxyapatite layer generated on the surface of the implanted scaffold can be chemically bonded to the host bone, which facilitates strong osseointegration. Overall, PGCZ possessed a superior ability to facilitate biomineralization and induce the generation of a hydroxyapatite layer *in vitro* under the synergistic effect of GelMA, CMCS, and ZIF-8@PDA, which would create an osteogenic microenvironment for new bone formation and bone maturation.

### Photothermal performance and Zn^2+^ release behavior of the hydrogel scaffolds

The development of an intelligent therapeutic platform with intrinsic photothermal effects and a stimuli-responsive ability to release active ingredients might be an important avenue to boost tissue regeneration and bacterial elimination in the diabetic microenvironment. As mentioned in the previous section, we successfully demonstrated the photothermal conversion capacity of ZIF-8@PDA nanoparticles. Next, the photothermal effects of various scaffold samples under 808 nm NIR irradiation were investigated. All scaffold samples immersed in PBS were subjected to NIR irradiation (1 W/cm^2^, 808 nm), and the temperature change was monitored via an infrared thermal imager. As displayed in **Figure 3G-H**, the temperature of the PGCZ scaffold had a striking temperature variation and rapidly increased to 53.2 ± 0.2 °C in the wet state within 5 min. In contrast, both the PCL and PGC scaffolds showed negligible temperature increases under the same irradiation conditions. Moreover, the as-prepared PGCZ scaffold exhibited excellent photothermal stability without significant variation in the maximum temperature during five on/off cycles of laser heating (**Figure S19**), supporting its use as a photothermal platform in repeated PTT. The exceptional photothermal effect of the PGCZ scaffold system greatly expands the potential applications of photoactivated biomaterial scaffolds, especially in the fields of tissue engineering and antibacterial therapy.

Both after bone injury and under diabetic pathological conditions, bone healing usually starts with post-traumatic slightly acidic milieu (pH~6.5) 55, 56, so it is essential to study the release of Zn^2+^ triggered by NIR irradiation at pH=6.5. In the following release experiment, the PGCZ scaffolds released the least amount of Zn^2+^ in neutral PBS (pH=7.4) (7.6 ± 0.5 ppm at 28 days) without NIR irradiation (**Figure 3I**). In comparison, weak acid (pH=6.5) and NIR irradiation strikingly accelerated the Zn^2+^ release rate (10.8 ± 0.4 ppm and 14.1 ± 0.2 ppm, respectively) at 28 days. A reasonable explanation for this controlled release behavior is not only the pH-responsive degradation of ZIF-8 and the pH sensitivity of the PDA layer, but also the enhanced diffusion effect at elevated temperatures 38. Furthermore, when NIR irradiation was applied to acidic PBS (pH=6.5), which simulates the diabetic microenvironment, the release of Zn^2+^ was significantly accelerated, with a cumulative release of 18.8 ± 0.3 ppm at 28 days, signifying that periodic NIR irradiation could effectively stimulate the release of Zn^2+^ under acidic diabetic conditions.

The sustained (up to 28 days) and controlled (NIR/pH dual responsiveness) release profile of Zn^2+^ allowed the PGCZ scaffold to meet the demands of the long-term bone repair process *in vivo*. It has been proven that PDA decoration has a protective effect against the degradation of ZIF-8 and is effective at controlling the release of Zn^2+^ 38. As an essential trace element of the human body, Zn^2+^ has been confirmed to play a fundamental role in various biological activities, such as cell proliferation and osteogenic differentiation, and can regulate the bone immune microenvironment in the early stage of osteogenesis 57. Moreover, Zn^2+^ possesses an ideal antibacterial effect and can be easily metabolized and cleared without obvious side effects 58. When applied to diabetic bone defects, it is envisioned that PGCZ has a controlled and on-demand release behavior under the combined effect of low pH and NIR irradiation at defect sites, which was effective at modulating the local immune microenvironment and facilitating tissue regeneration, ultimately leading to the intelligent treatment of diabetic bone defects. To summarize the above results, it was speculated that this soft-hard combined PGCZ scaffold system might be an excellent candidate for accelerated bone healing in the diabetic microenvironment via the synergistic effect of mild photothermal treatment and stimuli-responsive Zn^2+^ release.

### *In vitro* cytocompatibility and osteogenic activity of the hydrogel scaffolds

**Figure 4:**
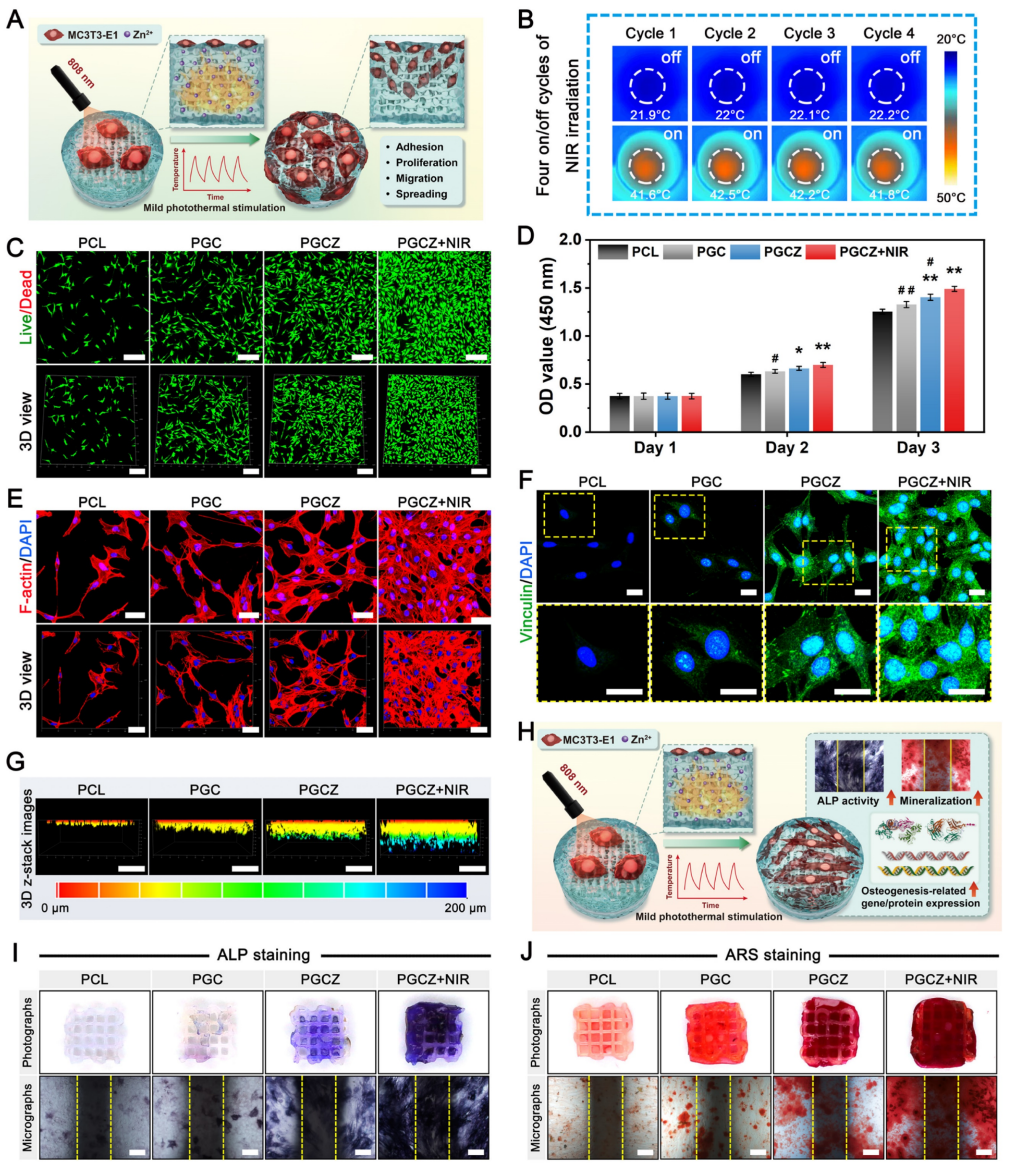
***In vitro* cytocompatibility and osteogenic potential. (A)** Schematic illustration of the co-culture system. **(B)** Infrared thermal images of the cell/scaffold complex during the four on/off cycles of NIR irradiation (1 W/cm^2^, 808 nm). **(C)** Live/dead staining images of MC3T3-E1 cells after culturing for 3 days. Scale bar: 200 μm. **(D)** CCK-8 assay of MC3T3-E1 cells after culturing for 1, 2, and 3 days. **(E)** Confocal immunofluorescence images of cytoskeleton staining for MC3T3-E1 cells after culturing for 3 days. (green: F4/80; red: F-actin; blue: DAPI). Scale bar: 50 μm. **(F)** Immunofluorescence staining images of vinculin (green: vinculin; blue: DAPI). Scale bar: 25 μm. **(G)** 3D reconstructed confocal images of MC3T3-E1 cells after culturing for 3 days. Scale bar: 200 μm. **(H)** Schematic illustration of the induction of osteogenesis in MC3T3-E1 cells. **(I)** ALP staining images of different cell/scaffold complexes after 7 days of co-culture. The yellow dotted lines indicate the boundaries of the scaffold struts. Scale bar: 200 μm. **(J)** ARS staining images of different cell/scaffold complexes after 14 days of co-culture. The yellow dotted lines indicate the boundaries of the scaffold struts. Scale bar: 200 μm. Data are presented as the mean ± SD (n = 3). *P < 0.05 and **P < 0.01 indicate significant differences compared with the PCL group. ^#^P < 0.05 and ^# #^P < 0.01 indicate significant differences compared with the PGCZ+NIR group.

In addition to suitable physiochemical properties, good cytocompatibility is a prerequisite for the application of biomaterial scaffolds in bone tissue repair and regeneration. Therefore, to ascertain the *in vitro* biological performance of the mild photothermal platform, MC3T3-E1 cells, a kind of osteoprogenitor cell, were co-cultured on PGCZ scaffolds and subjected to periodic NIR irradiation, as schematically shown in **Figure 4A**. During the four on/off cycles of NIR irradiation, an infrared thermal imager was used to monitor the change in temperature, ensuring that the temperature reached 42 ± 1 °C (**Figure 4B**). The viability and proliferation of MC3T3-E1 cells were detected by live/dead staining and CCK-8 assays. After being cultured for 3 days, the fluorescence images illustrated that almost all the cells in the three types of hydrogel scaffolds were alive, and no obvious red fluorescence-labeled dead cells appeared (**Figure 4C**). In particular, the number of live MC3T3-E1 cells in the PGCZ scaffold group was significantly higher than that in the PCL and PGC scaffold groups, as evidenced by an increased number of green-stained cells. The introduction of the ZIF-8@PDA-loaded hydrogel could provide a superior ECM-mimicking microenvironment, which expanded the growth space and provided favorable niches for cell adhesion and proliferation. Beyond that, an appropriate concentration of Zn^2+^ has been found to be beneficial for the proliferation and differentiation of osteoblasts 57. Significantly, with the assistance of periodically applied NIR stimulation, the proliferation level of the PGCZ+NIR group was obviously higher than that of the PGCZ group, indicating that the combination of hydrogel functionalization and on-demand NIR irradiation was more effective in favoring osteoblast growth and survival *in vitro*. According to the quantitative results of the CCK-8 assay, the number of MC3T3-E1 cells in all groups gradually increased within the co-culture period (**Figure 4D**), indicating good proliferative activity. Encouragingly, the NIR-treated PGCZ scaffold exhibited optimal proliferation efficiency on day 3, which was in line with previous live/dead cell staining results. Owing to the increased accessibility of the electron transport chain, mild photothermal stimulation has been proven to exert a stimulatory effect on cell metabolism and cell growth 59. Furthermore, the mildly elevated temperature induced by PGCZ+NIR could also interact with the released Zn^2+^ ions, thereby producing a stepwise and synergistic amplification biological effect on cell proliferation.

To further validate that the NIR-mediated PGCZ scaffold could influence cellular behavior, cytoskeleton staining was performed after being co-cultured for 3 days. As shown in **Figure 4E**, MC3T3-E1 cells could attach and spread efficiently on all scaffolds, indicating their ability to support cell adhesion. Specifically, the cell spreading area on the surface of the original PCL scaffold was small, and no filopodium was observed, implying limited cell extension and a poorly developed cytoskeleton. In contrast, the cells on the PGC and PGCZ scaffolds displayed better flat-spreading and elongated spindle-shaped morphology. Under the action of ZIF-8@PDA, polygonal or spindle-shaped osteoblasts differentiated from MC3T3-E1 cells in the PGCZ scaffold group were detected, with more obvious filamentous prosthetic feet and flat membranes, suggesting enhanced cell-scaffold interactions. In addition, we discovered that more cells adhered to the surface of the PGCZ+NIR group, which also had the largest F-actin spreading area (**Figure S20**), suggesting that PGCZ combined with mild photothermal treatment significantly promoted cell adhesion, proliferation, and spreading. The interaction between the ECM and the F-actin cytoskeleton involves the formation of organelles called focal adhesions (FAs), which can trigger signaling mechanisms that converge to promote cell proliferation, adhesion, and differentiation 60. The CLSM observation in **Figure 4F** shows that higher expression levels of vinculin were observed in the PGCZ+NIR group than in the PGCZ and PGC groups, whereas no significant positive expression was detected in the PCL group. Moreover, FAs were mainly distributed both in the center and periphery of the cell, indicating that the integration of the ECM-like bioactive hydrogel into the PCL scaffold was conducive to cell adhesion, which could be further promoted by mild thermal treatment.

A previous study confirmed that mild thermal stimulation could alter the cytoskeleton and integrin signaling, thereby promoting osteoblast differentiation and bone regeneration 61. To better observe the growth and distribution of osteoblasts in the scaffolds, 3D images of each scaffold after culturing cells for 3 days were also obtained by CLSM (**Figure 4G**). According to 3D-reconstructed CLSM images, both the number and migration distance of the infiltrating cells within the PGCZ scaffold were significantly higher than those in the PGC and PCL groups, indicating that the introduction of the ZIF-8@PDA-loaded hydrogel was able to afford an ECM-like biomimetic microenvironment and bioactive components (e.g., Zn^2+^) for cell enrichment and migration. Besides, the stiffness of the PGCZ scaffold can be sensed by cells through FA, thereby affecting cell adhesion and migration behavior, and this chemotactic effect could be further improved with the aid of NIR irradiation.

These features synergistically influence cell behavior and function, consistent with the findings of previous studies showing that mild photothermal effects play a positive regulatory role in cell adhesion, proliferation, spreading, and migration 62. Overall, the results obtained in this study provide further support for the role of NIR-assisted mild hyperthermia in favoring cellular behaviors (e.g., cell adhesion, proliferation, migration, and spreading).

Except for the requirement for cytocompatibility, investigating the influence of the PGCZ photothermal platform on osteogenic differentiation is also crucial because it can provide a theoretical basis for further clinical applications. Therefore, to determine the *in vitro* osteogenic activity of the MC3T3-E1 cells, alkaline phosphatase (ALP) staining, alizarin red S (ARS) staining, and expression of osteogenesis-related markers were performed (**Figure 4H**). ALP staining and corresponding quantitative analysis are shown in **Figure 4I** and **Figure S21A**. Compared with the PCL scaffold, the PGC scaffold slightly upregulated the expression of ALP on day 7, while the PGCZ composite scaffold dramatically promoted ALP expression due to the degradation-produced Zn^2+^ from ZIF-8@PDA. In addition, the combination of the PGCZ scaffold and om-demand mild hyperthermia induced the most compact ALP-positive staining. The introduction of GelMA and CMCS endows the PCL scaffold with a certain osteogenic ability 12, and the addition of ZIF-8@PDA further reinforced this effect for the PGCZ scaffold, which was ascribed to the inherent osteogenic properties of Zn^2+^^25^. It has been demonstrated that osteogenic differentiation and bone regeneration can be promoted by bioactive ions, such as Zn, Sr, and Si ions 63. Especially, Zn^2+^ ions have been reported to stimulate bone growth and mineralization, as well as increase osteoblast activity by modulating key cellular signaling pathways, including the MAPK and Wnt/β-catenin signaling pathways 26, 32. In the present study, the cells treated with PGCZ+NIR showed the best osteogenic differentiation capacity, which might be derived from the fact that NIR light triggered the release of more bioactive Zn^2+^ as well as the mild hyperthermia-mediated upregulation of HSPs (**Figure S22**). As demonstrated in previous studies, the collagen-specific chaperone HSP47 is essential for the biosynthesis and maturation of Col-I related to osteogenesis 18. In this case, the NIR-irradiated PGCZ scaffold platform could combine physical (photothermal) stimuli and biochemical (bioactive Zn^2+^) cues to jointly promote osteogenesis by activating key cellular signaling pathways and upregulating the expression of HSPs. Next, calcium mineral deposition, a vital indicator of late osteogenesis, was evaluated via ARS staining and corresponding quantitative data. After 14 days, a large amount of calcium deposition was observed in the PGC and PGCZ groups, and the highest mineralization level with densified distribution of calcium nodules was presented in the PGCZ+NIR group, while only a small amount of insignificant mineral matrix formation was produced in the PCL group (**Figure 4J**). Quantitative analysis of ECM mineralization demonstrated a consistent trend (**Figure S21B**), further confirming the exceptional osteoinductive potential of the PGCZ+NIR group. The action of mild photothermal stimulation upregulated the expression of HSPs, co-promoting the osteogenic differentiation of MC3T3-E1 cells with bioactive Zn^2+^ ions. Moreover, our work demonstrated that both F-actin levels and FA expression were significantly increased in the PGCZ+NIR group, supporting that PGCZ combined with on-demand NIR irradiation might improve the osteogenic differentiation of cells by accelerating the formation of FAs and actin polymerization, which strongly coincides with previous findings 43. Therefore, it is speculated that with the aid of mild hyperthermia, the NIR-irradiated PGCZ platform not only greatly promoted the attachment, proliferation, migration, and spreading of osteoblasts, but also induced a stronger osteogenic response *in vitro*.

Next, the expression levels of osteogenesis-related genes, including *Col-1*, *Runx2*, *OPN*, and *OCN*, were examined via quantitative real-time polymerase chain reaction (qRT-PCR) (**Figure S23A**). The PGCZ and PGCZ+NIR groups had significantly higher expression levels of these genes than did the PGC and PCL groups, especially for *Runx2* and *OPN* in the PGCZ+NIR group. Runx2, a critical osteogenesis-specific transcription factor, is considered an early marker of osteogenic differentiation that can trigger the expression of other osteogenic factors, such as ALP, Col-1, OPN, and OCN. Meanwhile, OPN is a highly phosphorylated glycoprotein involved in bone matrix organization and deposition 60. This phenomenon was also verified by immunofluorescence staining and corresponding quantitative statistics, as shown in **Figure S23B** and **Figure S24**. The PGCZ+NIR group showed the highest positive expression of Runx2 and OPN, followed by the PGCZ and PGC groups, demonstrating the excellent ability of these groups to induce osteogenic differentiation of MC3T3-E1 cells. These results suggested that the introduction of an ECM-like biomimetic hydrogel matrix into the 3D-printed PCL scaffold could stimulate osteogenesis through the desired continuous release of Zn^2+^ ions and mild photothermal effects, which are assumed to be beneficial for augmented bone regeneration. Our findings also align with previous studies reporting the significant enhancement of ALP activity, calcium deposition, and osteogenic gene expression in ZIF-8-based scaffolds 64. Beyond that, mild photothermal treatment upregulated the expression of HSPs, which have been found to activate osteogenic signaling pathways, such as the MAPK and TGF-β pathways, and participate in bone metabolism 61. From the *in vitro* biocompatibility and osteogenic test results, the photoactivated PGCZ scaffold is anticipated to become a potential therapeutic platform for bone defect repair and regeneration.

### Antioxidant and immunomodulatory performance

**Figure 5:**
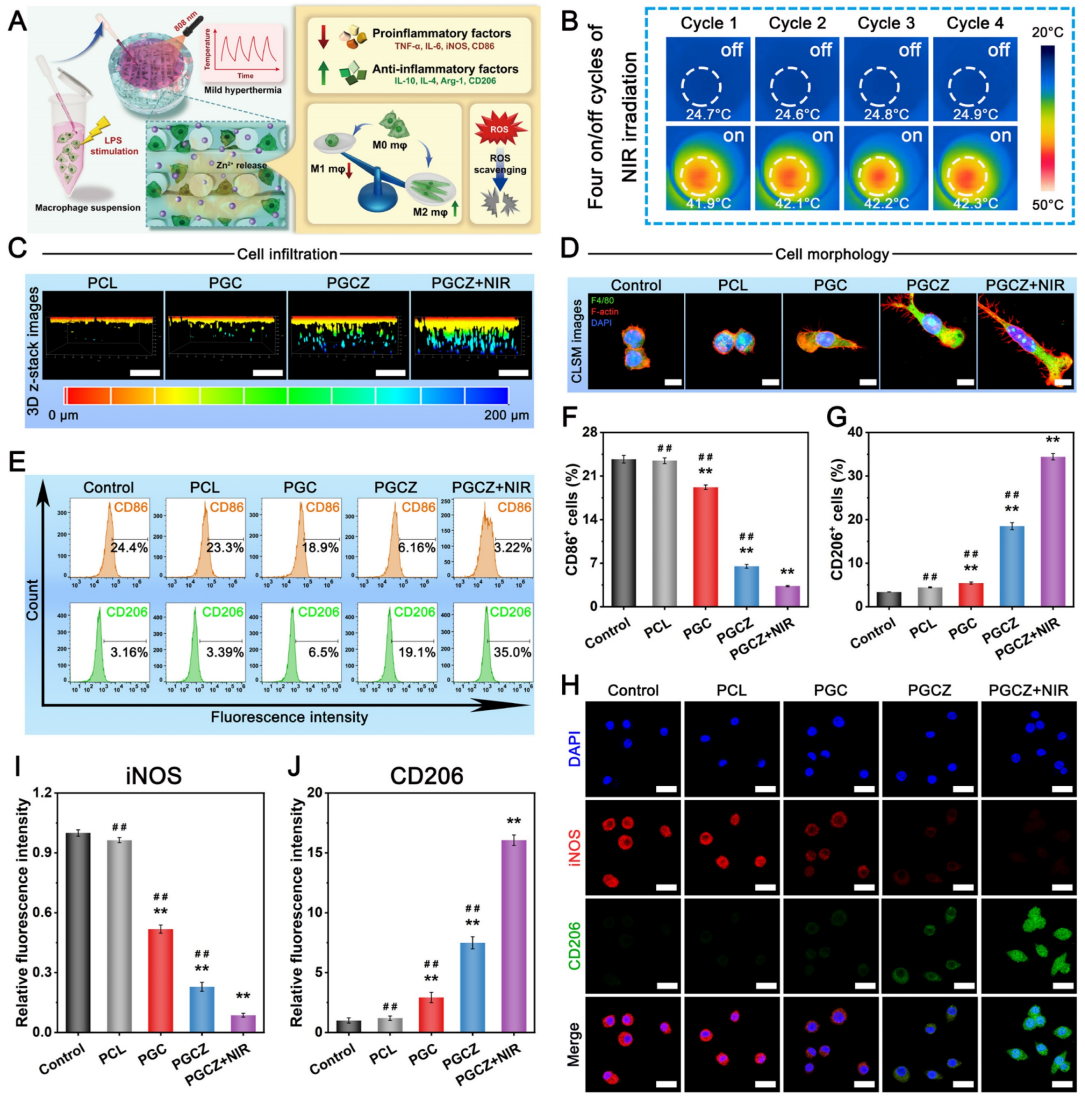
***In vitro* immunomodulatory performance. (A)** Schematic illustration of immunomodulation induced by the photoactivated PGCZ hybrid scaffold. (B) Infrared thermal images of the cell/scaffold complex during the four on/off cycles of NIR irradiation (1 W/cm^2^, 808 nm). **(C)** 3D reconstructed confocal images of macrophages after culturing for 3 days. Scale bar: 200 μm. **(D)** Confocal immunofluorescence images of cytoskeleton staining for macrophages after co-culturing for 3 days. (red: F-actin; blue: DAPI). Scale bar: 5 μm. **(E)** Flow cytometry analysis and **(F-G)** corresponding quantification of macrophage phenotypes after co-culturing for 3 days. **(H)** Immunofluorescence staining images and **(I-J)** corresponding quantitative analysis of iNOS and CD206 (red: iNOS; green: CD206; blue: DAPI). Scale bar: 20 μm. Data are presented as the mean ± SD (n = 3). *P < 0.05 and **P < 0.01 indicate significant differences compared with the control group. ^#^P < 0.05 and ^# #^P < 0.01 indicate significant differences compared with the PGCZ+NIR group.

In the diabetic inflammatory microenvironment, overactivation of proinflammatory M1 macrophages and overexpression of ROS escalate inflammatory cell infiltration and trigger a local “cytokine storm”, ultimately leading to deterioration of regenerative capacity. The accumulation of excessive ROS results in sustained cell/tissue injury and induces amplification of the inflammatory cycle, which further disrupts osteogenesis and angiogenesis 4, 65. Therefore, biomaterial-based bone repair scaffolds with excellent antioxidant activity can powerfully scavenge ROS and reverse high oxidative stress, which is helpful for diabetic bone healing. It has been proven that LPS-activated macrophages promote proinflammatory cytokine production and ROS generation 5. In this work, RAW264.7 cells were pretreated with LPS to mimic the diabetic inflammatory microenvironment and induce macrophages to the M1 phenotype (classically activated macrophages), thus leading to the production of numerous free radicals (i.e., ROS) and chronic inflammation (**Figure 5A**). For the NIR irradiation treatment, “on-off” cyclic heating was continuously conducted four times, as mentioned above, with the peak radiation temperature remaining at 42 ± 1 °C (**Figure 5B**).

After that, ROS levels were analyzed through 2',7'-dichlorodihydrofluorescein diacetate (DCFH-DA) staining and flow cytometry. As shown in **Figure S25A**, the intracellular ROS level (green fluorescence) was lower in the PGC, PGCZ, and PGCZ+NIR groups than in the LPS-stimulated control and PCL groups, indicating the strong antioxidant properties and ROS scavenging ability of these materials. In particular, the most substantial reduction in intracellular ROS was observed in the PGCZ+NIR group, as demonstrated by the small amount of fluorescent signals observed. The results of flow cytometry analysis also demonstrated that the percentage of fluorescence-positive cells was significantly decreased in the PGCZ (31.1%) and PGCZ+NIR (13.3%) groups than in the control (78%), PCL (77%), and PGC (60.2%) groups (**Figure S25B**). The main reason for the strong capacity to scavenge ROS was that NIR-mediated mild heat stimulation accelerated the disassembly of ZIF-8@PDA and facilitated adequate contact between the reductive components (phenolic hydroxyl groups and Zn^2+^) and free radical detection reagents 22. According to the results of the ROS scavenging assay, we assumed that the NIR-irradiated PGCZ scaffold system has great potential to alleviate inflammation and protect cells from oxidative damage by eliminating excessive ROS in the diabetic bone microenvironment.

Multiple studies have indicated that M1 macrophage overactivation and suppression of M2 macrophage polarization can result in imbalanced M1/M2 macrophages under diabetic conditions. This imbalanced macrophage polarization impairs bone healing by disrupting paracrine signaling, leading to the interference of osteogenesis and angiogenesis in the bone microenvironment 66. During the initial phase of inflammation, macrophages are important immune cells, and their polarization toward the anti-inflammatory phenotype (M2) can drive the transition from the inflammatory phase to the proliferation and remodeling phases, which is beneficial for functional tissue regeneration 12. Consequently, the rational regulation of M1-to-M2 macrophage polarization (i.e., immunomodulation) has profound effects on bone healing in the pathological milieu of diabetes. After bone injury, the infiltration of macrophages is an important step in angiogenesis and tissue regeneration. As displayed in **Figure 5C**, 3D-reconstructed CLSM images demonstrated that the cells co-cultured on the PGCZ scaffold exhibited strong migration and penetration capabilities, followed by those on the PGC and PCL groups, and that the penetration of macrophages became more obvious after periodic NIR irradiation, along with more cell growth to the interior of the scaffold. In addition, a much denser macrophage distribution was observed on the PGCZ+NIR group, covering large areas of the scaffold. In sharp contrast, cell infiltration was almost invisible inside the PCL scaffold, in which cells mainly grew on the top surface of the scaffold. This result indicated that functionalization of the bioactive hydrogel facilitated macrophage infiltration, which was ascribed to the 3D ECM-mimicking microenvironment and ZIF-8@PDA. And this effect could be further enhanced by appropriate NIR irradiation owing to the desired continuous release of active Zn^2+^ ions and mild photothermal effects.

Changes in cell morphology, including cell proliferation and migration, stem cell differentiation, and macrophage polarization, are related to changes in the functional states of cells. It has been demonstrated that M0/M1 macrophages exhibit a round-like shape, while M2 macrophages are spindle-shaped and spread better 35. Cytoskeleton staining results revealed that the RAW264.7 cells co-cultured in the PGCZ and PGCZ+NIR groups exhibited obvious pseudopod-like structures and elongated morphologies with significant M2 activation characteristics, especially those of the PGCZ+NIR group (**Figure 5D**). Furthermore, macrophages cultured on hydrophilic surfaces were reported to exhibit increased potential to differentiate toward the anti-inflammatory M2 phenotype 67, which was in agreement with our current findings. In comparison, LPS-treated macrophages in the control and PCL groups exhibited a round, dot-shaped morphology with short pseudopodia, indicating the M0/M1 phenotype. Recent studies have demonstrated the synergistic effect of PDA and mild heat stimulation on expediting the substantial development of adhesive structures on macrophages 20. These findings are in line with our present results and further suggested that the combination of ZIF-8@PDA and MPTT not only promoted cell stretching and spreading but also facilitated the M1-to-M2 phenotype switch.

To further confirm the polarization tendency of M1 and M2 macrophages, flow cytometry analysis was performed on RAW264.7 cells. As illustrated in **Figure 5E-G**, the ratio of CD206^+^ macrophages in the PGCZ+NIR group was significantly higher than that in the PGCZ group, followed by that in the PGC group, demonstrating that ZIF-8@PDA loading and MPTT treatment could achieve a potent ability to induce the polarization of macrophages toward the M2 phenotype. Simultaneously, compared with those in the control and PCL groups, the percentage of CD86^+^ macrophages in the other three groups was significantly lower, with the PGCZ+NIR group having the lowest. The results of immunofluorescence staining showed a consistent trend (**Figure 5H**), with more robust iNOS fluorescence observed in the LPS-stimulated control and PCL groups, illustrating M1 macrophage activation and the successful induction of inflammation. On the contrary, the PGCZ and PGCZ+NIR groups displayed stronger fluorescence intensity of CD206 staining, especially the PGCZ+NIR group, indicating that the PGCZ scaffold promoted M2 macrophage activation, and that mild heat treatment further strengthened this effect (**Figure 5I-J**). All these results clearly demonstrated that the NIR-irradiated PGCZ platform could exert desirable immunomodulatory effects to suppress M1 macrophage polarization while activating macrophage transformation toward the M2 phenotype, resulting in balanced M1/M2 macrophages, which was advantageous for alleviating inflammatory reactions and promoting subsequent tissue regeneration.

Then, we assessed the expression of proinflammatory (M1) genes (*IL-6*, *TNF-α*, *iNOS*, and *CD86*) and anti-inflammatory (M2) genes (*IL-4*, *IL-10*, *Arg-1*, and *CD206*) in RAW264.7 cells using qRT-PCR. As depicted in **Figure S26A**, the expression of *IL-6*, *TNF-α*, *iNOS*, and *CD86* in the LPS-stimulated control group was significantly increased, indicating successful polarization of macrophages to the proinflammatory M1 phenotype. In contrast, PGCZ exhibited a significant inhibitory effect on the overexpression of these proinflammatory factors as well as induced much higher expression levels of anti-inflammatory factors (**Figure S26B**). These qRT-PCR results corroborated the trends observed via immunofluorescence and flow cytometry analyses, demonstrating that the PGCZ+NIR group could mediate the polarization of macrophages from the M1 to the M2 phenotype and ameliorate inflammation. This phenomenon could be explained by two potential pathways: 1) Zn^2+^ released from ZIF-8@PDA nanoparticles could inhibit inflammation by activating the P38 MAPK pathway and inhibiting the NF-κB signaling pathway 68; and 2) the PDA molecules on the nanoparticles could also inhibit the NF-κB signaling pathway and reduce LPS-induced ROS in macrophages to enhance their anti-inflammatory ability 69. In the present study, the PGCZ scaffold exhibited better immunomodulatory potential to accelerate the M1-to-M2 transition of macrophages than did PGC or PCL, which was ascribed to the ability of the ZIF-8@PDA-loaded bioactive hydrogel to enhance adhesion and pseudopod formation. Furthermore, the periodic NIR irradiation-derived MPTT at a mild fever-range temperature (42 ± 1 °C) accelerated the transition of M1-to-M2 macrophage polarization and induced the secretion of M2-featured cytokines by activating the PI3K/AKT1 signaling pathway 20. These data highlighted the synergistic effects of hydrogel functionalization and mild thermal stimulation on activating macrophage polarization to the M2 phenotype, which is expected to shorten the period of inflammation resolution.

### The influence of immunomodulation on angiogenesis

**Figure 6:**
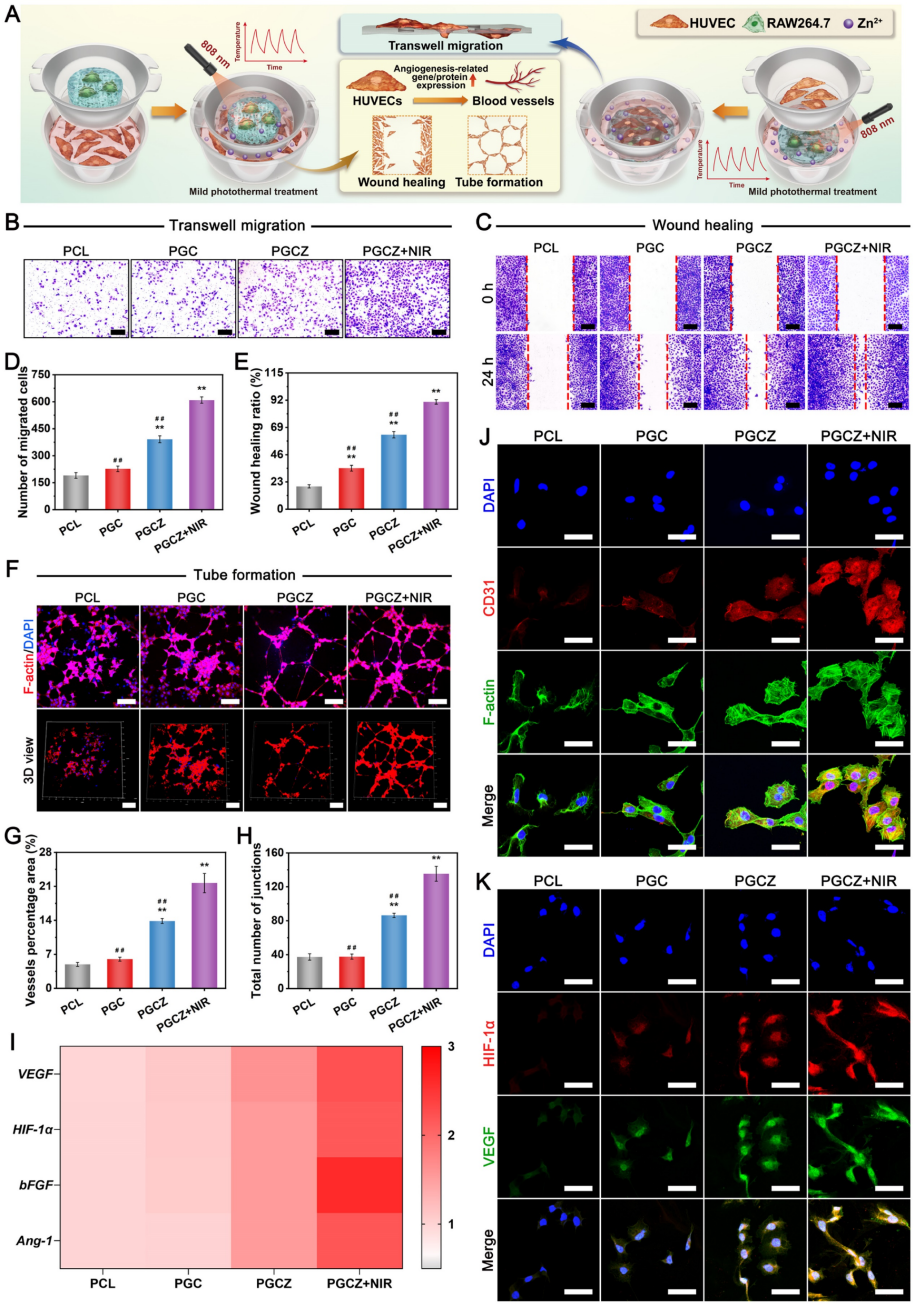
***In vitro* angiogenic potential regulated by macrophage polarization. (A)** Schematic illustration of the establishment of *in vitro* co-culture system and the subsequent induction of vascularization in HUVECs. **(B)** Crystal violet staining images of HUVECs after treatment and **(D)** corresponding quantification of migrated cells. Scale bar: 200 μm. **(C)** Optical images of the scratch wound healing assay for HUVECs after treatment and **(E)** corresponding quantification of the wound migration rate. Scale bar: 200 μm. **(F)** Confocal fluorescence images of the tube formation assay for HUVECs and corresponding quantitative analysis, including **(G)** vessel percentage area and **(H)** total number of junctions. Scale bar: 200 μm. **(I)** Relative mRNA expression of angiogenesis-related genes, including *VEGF*, *HIF-1α*, *bFGF*, and *Ang-1*, in HUVECs. **(J)** Immunofluorescence staining images of CD31 (red: CD31; green: F-actin; blue: DAPI). Scale bar: 20 μm. **(K)** Immunofluorescence staining images of HIF-1α and VEGF (red: HIF-1α; green: VEGF; blue: DAPI). Scale bar: 20 μm. Data are presented as the mean ± SD (n = 3). *P < 0.05 and **P < 0.01 indicate significant differences compared with the PCL group. ^#^P < 0.05 and ^# #^P < 0.01 indicate significant differences compared with the PGCZ+NIR group.

During the process of bone development and repair, the immune response is strongly correlated with angiogenesis and osteogenesis. Various studies have shown that a favorable immune microenvironment is able to promote vascular function through M2 macrophage-secreted anti-inflammatory cytokines and therapeutic growth factors, which emphasizes the importance of immunomodulation in vascularization and tissue regeneration 56, 70. To explore whether macrophages in response to the scaffolds with or without NIR irradiation could influence vascularization, a cell-scaffold co-culture model was established (**Figure 6A**). We first co-incubated macrophages on scaffolds with or without NIR radiation for 24 h to evaluate the secretion of macrophage-associated cytokines by using ELISA assay. As shown in **Figure S27**, the macrophages in the PGCZ+NIR group secreted the highest amounts of the anti-inflammatory cytokine (IL-10), followed by those in the PGCZ and PGC groups, which are produced mainly by M2 macrophages. In contrast, the expression level of the proinflammatory cytokine TNF-α, which is mainly secreted by M1 macrophages, was the lowest in the PGCZ+NIR group. As previously reported, IL-10 is one of the most important anti-inflammatory mediators that targets a variety of leukocytes and mainly inhibits excessive inflammatory responses.

IL-10 could also promote the transformation of macrophages from the M1 state to the M2 state, which would help to regulate the immune microenvironment during bone regeneration 71. More significantly, the secretion of pro-healing cytokines (VEGF and bFGF) was considerably increased in the PGCZ and PGCZ+NIR groups than in the PCL and PGC groups, especially in the PGCZ+NIR group. Both VEGF and bFGF are potent growth factors for the recruitment and function of endothelial cells, which in turn promote angiogenesis and vascular development 72. Collectively, these data indicated enhanced M2 macrophage polarization in the PGCZ+NIR group, which promoted the production of anti-inflammatory and pro-healing cytokines, such as IL-10, VEGF, and bFGF, thereby collaboratively accelerating vascularization and bone tissue regeneration.

A growing number of studies have demonstrated that vascular endothelial cell behaviors, including recruitment, migration, and angiogenic differentiation, are very important for tissue regeneration and bone repair. Simultaneously, the inflammatory cytokines and chemokines secreted by macrophages can effectively initiate angiogenesis at the initial stage after implantation 35. Therefore, to validate the effect of the M2 macrophage polarization-mediated microenvironment on angiogenesis, Transwell migration assays, scratch wound healing tests, and tube formation experiments were conducted, as they are the major and classic methods used to assess angiogenic potential. As shown in **Figure 6B**, all the treatments promoted the migration of HUVECs to some extent after 24 h, and the cells treated with PGCZ+NIR demonstrated the greatest migration and recruitment capabilities, followed by those in the PGCZ group. More specifically, the number of recruited cells in the PGCZ+NIR group was approximately 3.2-fold, 2.6-fold and 1.6-fold greater than that in the PCL, PGC, and PGCZ groups, respectively (**Figure 6D**), suggesting that the combination of PGCZ and periodic NIR irradiation created a beneficial immunomodulatory microenvironment for the accelerated migration and recruitment of HUVECs. The results of the scratch wound healing assay further demonstrated that the PGCZ+NIR group had a significant promotion effect on the migration of HUVECs (**Figure 6C**). After incubation for 24 h, we found that the wound-healing percentages of the PCL, PGC, PGCZ and PGCZ+NIR groups reached approximately 19.3%, 34.6%, 62.9%, and 90.6%, respectively (**Figure 6E**), similar to that in the Transwell assay. Because proper M2 macrophage polarization can facilitate the vascularization of endothelial cells through the secretion of VEGF and bFGF, we hypothesized that the photothermal PGCZ scaffold system could promote HUVEC migration and recruitment through M2 macrophage-mediated immunomodulation. This conjecture can be verified by ELISA, scratch wound healing assays, and Transwell experiments, as described above. These results highlighted the importance of crosstalk between macrophages and endothelial cells for vascularization, and revealed that the NIR-mediated PGCZ scaffold system could orchestrate a favorable immune microenvironment for angiogenesis through promoting the polarization of M2-type macrophages and the secretion of pro-healing cytokines, such as VEGF and bFGF.

Since neovascularization could provide necessary nutrients and oxygen to diabetic defect sites to promote the recruitment, proliferation, and subsequent osteogenic differentiation of osteoprogenitors, reconstruction of the skeletal vascular network is crucial for bone development and fracture healing. Next, an *in vitro* tube formation assay was performed to investigate the influence of reprogramming M2 macrophages on angiogenic differentiation in HUVECs (**Figure 6F**). After 6 h, the PGCZ and PGCZ+NIR groups exhibited more distinct and complete vascular-like network structures, while only some incomplete or sparse tube-like structures were formed in the PCL and PGC groups.

Consistently, the quantified levels of junctions and vessel percentage areas were profoundly better in the PGCZ and PGCZ+NIR groups than in the other groups (**Figure 6G-H**), with the PGCZ+NIR group showing the best pro-angiogenic effect. Besides, similar trends were also observed for the expression of angiogenesis-related genes, where the relative mRNA expression levels of *VEGF*, *HIF-1α*, *bFGF*, and *Ang-1* in the PGCZ+NIR group were significantly higher than those in the PGCZ group (**Figure 6I**), indicating superior angiogenic activity. The above data revealed that the migration and tube formation as well as the expression of angiogenic indicators of HUVECs can be promoted by PGCZ scaffold system-mediated immunomodulation, which is conducive to revascularization during the bone repair process. These results are in line with previous findings that M2 macrophages can generate a favorable immunomodulatory microenvironment to promote vascularization 56. Furthermore, the influence of the macrophage-mediated immune microenvironment on the expression of proteins in HUVECs was also investigated. As a vascular endothelial cell marker, CD31 is always used to indicate the tendency and potential of angiogenesis, and HIF-1α is an upstream regulator that can activate the transcription of downstream angiogenic genes, including VEGF and Ang-1^51^. Furthermore, VEGF is an angiogenesis-related protein that can bind to the VEGF receptor and activate downstream molecules to promote endothelial cell proliferation, differentiation, and remodeling during angiogenesis 73. From the results of the immunofluorescence staining analysis, the expression levels of CD31, VEGF, and HIF-1α were the highest in the PGCZ+NIR group, followed by those in the PGCZ and PGC groups, and were the lowest in the PCL group (**Figure 6J-K**). Combined with the results of the ELISA assay, it could be inferred that M2 macrophages boost the angiogenesis of HUVECs via the expression of VEGF and HIF-1α. Our previous work confirmed that immunomodulation had the ability to promote vascular function through M2 macrophage-secreted anti-inflammatory cytokines and therapeutic growth factors 39, 74. In this work, the same conclusion was obtained, and another reason for the promotion of vascularization may be partially attributed to the release of bioactive Zn^2+^ upon degradation. To sum up, these findings corroborated the gene expression results, further demonstrating that PGCZ-induced M2 macrophages promoted angiogenesis under mild NIR irradiation.

During the entire process of scaffold-mediated tissue regeneration, macrophages can shift phenotypes during the initial inflammatory response, leading to the secretion of various cytokines to regulate vascular regeneration and bone formation 60. Considering the above analysis, it could be shown that the M2 macrophage-enriched tissue microenvironment generated from the PGCZ+NIR system exhibited a better ability to promote angiogenesis than the other groups. This finding is in accordance with previous studies showing that the activation of M2 macrophages could facilitate the production of key cytokines and growth factors, such as IL-10, VEGF, and bFGF, to promote cell proliferation, migration, recruitment, ECM deposition, and angiogenesis for enhanced bone healing 75. In addition, this stronger pro-angiogenic ability also benefited from the synergetic enhancement of NIR-triggered Zn^2+^ release, further confirming the necessity of ZIF-8@PDA loading. Previous studies have revealed that bioactive Zn^2+^ participates in angiogenesis by activating the PI3K/AKT pathway 32. Thus far, the results obtained in this study were well in line with our design and hypothesis, with a perfectly ordered polarization transition of macrophages toward the M2 phenotype, and could further promote subsequent revascularization through paracrine effects.

### Antibacterial properties

**Figure 7:**
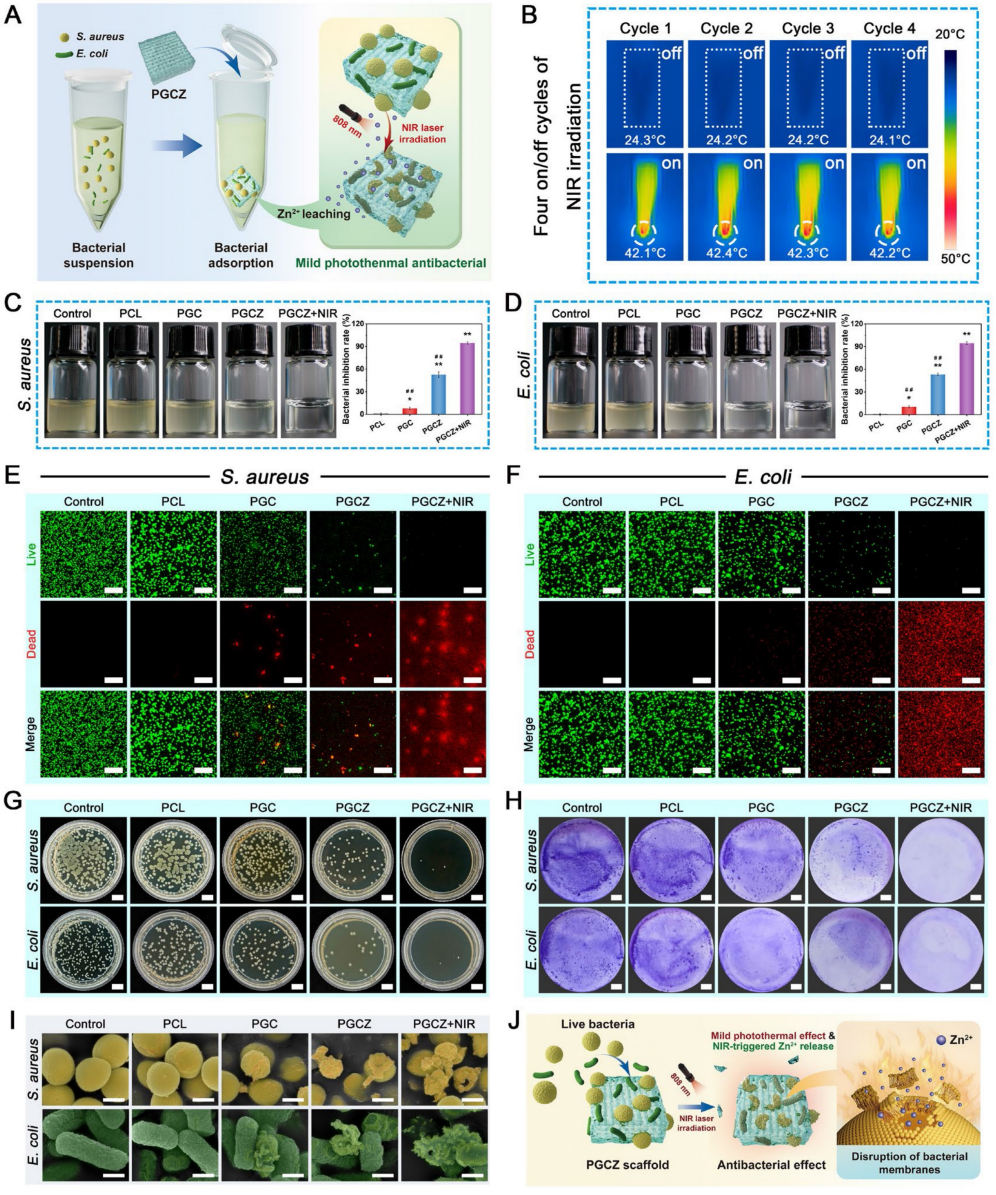
***In vitro* antibacterial activity. (A)** Schematic diagram of the antibacterial performance of the photoactivated PGCZ hybrid scaffold. **(B)** Infrared thermal images of the bacterial/scaffold complex under NIR irradiation (1 W/cm^2^, 808 nm) for four on/off cycles. Agar plate counting assay of **(C)** *S. aureus* and **(D)** *E. coli* showing antibacterial potential after different treatments and quantitative analysis of the bacterial inhibition rate. Confocal images of live/dead bacterial staining of **(E)** *S. aureus* and **(F)** *E. coli* after different treatments. Scale bar: 50 μm. **(G)** Photographs of *S. aureus* and *E. coli* bacterial colonies after different treatments. Scale bar: 1 cm. **(H)** Crystal violet staining images of *S. aureus* and *E. coli* bacterial biofilms after different treatments. Scale bar: 2 mm. **(I)** SEM images of *S. aureus* and *E. coli* after different treatments. Scale bar: 500 nm. **(J)** Schematic illustration of the antibacterial mechanism of the photoactivated PGCZ scaffold. Data are presented as the mean ± SD (n = 3). *P < 0.05 and **P < 0.01 indicate significant differences compared with the PCL group. ^#^P < 0.05 and ^# #^P < 0.01 indicate significant differences compared with the PGCZ+NIR group.

Bacterial infection after bone injury or bone implantation surgery remains a major clinical challenge that requires extensive surgical intervention and long-term antibiotic therapy 58. Biomaterial scaffolds with good bacteria-killing properties for bone healing are crucial, especially for treating diabetic bone defects. Zn^2+^ ions are natural antibiotics with strong antibacterial ability; thus, Zn-based biomaterials have been widely used for antibacterial applications 76. As the PGCZ scaffold system features excellent photothermal effects and controlled Zn^2+^ release *in vitro*, it is expected to achieve remarkable photothermal antibacterial efficacy (**Figure 7A**). Both Gram-positive (*S. aureus*) and Gram-negative (*E. coli*) bacteria were used to investigate the *in vitro* antibacterial ability of the different samples with or without NIR irradiation (**Figure 7B**). Turbidimetric analysis and quantitative data illustrated that both the PGCZ and PGCZ+NIR groups showed good inhibitory effects on the growth of *S. aureus* and *E. coli* (**Figure 7C-D**). Particularly, the antibacterial ratio of the PGCZ+NIR group reached above 90% for both *S. aureus* and *E. coli*, which was mainly assigned to the outstanding photothermal effects together with the thermally triggered release of Zn^2+^ from the PGCZ scaffold. The potential antibacterial mechanisms of the PGCZ scaffold system might involve the interplay between the ZIF-8@PDA nanoparticles and the membrane under mild NIR irradiation, and the release of Zn^2+^. In addition, the hydrophilicity of PDA was shown to favor bacterial adhesion and capture, which could further amplify the antibacterial efficiency of the PGCZ scaffold. The introduction of CMCS also exerted a positive effect on the improvement of antibacterial properties. By comparison, no obvious antibacterial activities were found for the control or PCL groups. In summary, the PGCZ scaffolds markedly inhibited the proliferation of *S. aureus* and *E. coli*, especially after 808 nm NIR exposure, and the antibacterial effect was more significant. Live/dead bacteria staining was then performed to visualize the antibacterial effects of the scaffold system. As shown in **Figure 7E-F**, the red fluorescence intensity of dead bacteria in the PGCZ+NIR group was the highest, followed by that in the PGCZ and PGC groups. As expected, both the PGC and PGCZ samples displayed good antibacterial abilities against *S. aureus* and *E. coli*, especially the latter, which could be derived from the intrinsic antibacterial properties of CMCS and Zn^2+^. This antibacterial trend is consistent with the results of the turbidimetric test analysis. Furthermore, the NIR-irradiated PGCZ (PGCZ+NIR) group had a better antibacterial effect than the PGCZ group, and the effect became more pronounced as the Zn^2+^ ions continued to be released from the scaffolds. The standard plate counting assay showed similar results to those of live/dead bacteria staining. As displayed in **Figure 7G**, *S. aureus* and *E. coli* colonies were densely distributed in the control and PCL groups. However, significantly fewer colonies were observed in the PGCZ group. In particular, upon periodic NIR irradiation, the bacteria in the PGCZ+NIR group were almost invisible. The quantitative data also showed that the bacterial survival ratio of *S. aureus* colonies in the PGCZ+NIR group was approximately 4.5%, while that in the PGCZ and PGC groups was approximately 36% and 86%, respectively (**Figure S28**), indicating that the PGCZ scaffold could significantly inhibit bacterial growth under NIR irradiation. The synergistic antibacterial actions of Zn^2+^ and CMCS were responsible for the enhanced antibacterial efficiency. Upon exposure to 808 nm NIR light irradiation, the MPTT-improved Zn^2+^ permeability made the bacteria highly susceptible to antibacterial Zn^2+^ and CMCS, which further amplified the antibacterial activity.

After confirming the excellent antibacterial properties of the PGCZ scaffold with NIR irradiation, the biofilm elimination ability of the scaffold was also verified via crystal violet staining. Since biofilm formation is an important cause of persistent bacterial infection that significantly inhibits bone healing under diabetic conditions, efficient biofilm removal is also a desirable property of biomaterials 77. As shown in **Figure 7H**, compared with those of the control and PCL groups with intact biofilms, the biofilms of *S. aureus* and *E. coli* treated with the PGCZ scaffold were significantly eliminated, especially with the help of periodic NIR irradiation, indicating that the combination of ZIF-8@PDA and mild photothermal effects was effective in disrupting biofilm structure and membrane integrity. The quantification of the remaining biofilm biomass of both bacteria under different treatments also showed the same trend (**Figure S29**), supporting the superior efficacy of the photoactivated PGCZ scaffold system in destroying and removing established bacterial biofilms. Furthermore, we evaluated the antibacterial capability of the PGCZ scaffold system against a model biofilm consisting of the most common bacteria of suppurative infected bone defects (i.e., *S. aureus*) 78. As shown in **Figure S30**, uniform and dense bacterial biofilms with green fluorescence were formed in the control and PCL-treated groups, indicating an intact 3D biofilm structure with highly active* S. aureus*. Although biofilms with green fluorescence predominated in the PGC scaffold-treated group, some dead bacteria with red fluorescence could also be detected, partially due to the efficient antibacterial activity of the CMCS. In contrast, the *S. aureus* biofilm was severely disrupted in the PGCZ+NIR group, with thin and dispersed features. Simultaneously, intense red fluorescence was observed in the biofilm treated with the PGCZ scaffold, which became more obvious after being subjected to NIR stimulation, indicating that a substantial number of bacteria were killed, consistent with the results of the live/dead staining assay. These results confirmed that the synergistic antibacterial ability of the released Zn^2+^ and photothermal treatment could prevent biofilm formation and simultaneously lead to bacterial death.

To further explore the antibacterial behavior of the NIR-irradiated PGCZ system, the morphological changes in both *S. aureus* and *E. coli* after treatment were observed via SEM. As shown in** Figure 7I**, large amounts of *S. aureus* and *E. coli* with normal shapes and intact membranes were detected in the control and PCL groups. In contrast, after being co-cultured with PGC or PGCZ scaffold (especially the latter), the cell membranes of both *S. aureus* and *E. coli* collapsed and were damaged to varying degrees. Under NIR stimulation, combined with the photothermal effect of the ZIF-8@PDA nanoparticles, it is evident that the structural integrity of the bacteria was obviously impaired, leading to leakage of the bacterial cytoplasm and a reduction in the number of bacteria, further indicating the effectiveness of the designed mild photothermal antibacterial platform. Generally, PCL could not prevent the adhesion of bacteria or the formation of biofilms, while PGCZ achieved strong antibacterial and antibiofilm activities with the aid of periodic NIR irradiation, revealing the synergetic cooperation of mild photothermal effects, NIR-triggered Zn^2+^ release, and intrinsic antibacterial CMCS.

Herein, a well-founded bactericidal mechanism based on the synergistic intrinsic antibacterial property of Zn^2+^ and mild hyperthermia therapy triggered by the engineered PGCZ scaffold for actively killing bacteria and inhibiting biofilm formation is visually shown in **Figure 7J**. As reported in the previous literature, Zn^2+^ could cause bacterial death by damaging the integrity of the bacterial membrane, and the photo-triggered mild thermal effect further improved the bacterial sensitivity to antibacterial Zn^2+^ ions under elevated temperature, leading to synergistic antibacterial activity 28. In view of the structural and compositional characteristics, the engineered PGCZ scaffold system exhibited controlled and multi-stimuli-responsive Zn^2+^ release behavior under periodic NIR irradiation, and this intelligent on-demand delivery is anticipated to provide long-term physical (photothermal) and chemical (Zn^2+^ delivery) intervention for antibacterial therapy. However, further investigations are needed to systematically understand the specific antibacterial mechanisms of the photoactivated PGCZ scaffold and its precise impact on inhibiting biofilm formation in the context of specific applications. Taken together, our proposed PGCZ scaffold has outstanding antibacterial and antibiofilm properties upon moderate photothermal radiation, which is extremely effective in preventing bacterial infections during the early implantation of bone implants.

### *In vivo* assessment of subcutaneous inflammation and angiogenesis

**Figure 8:**
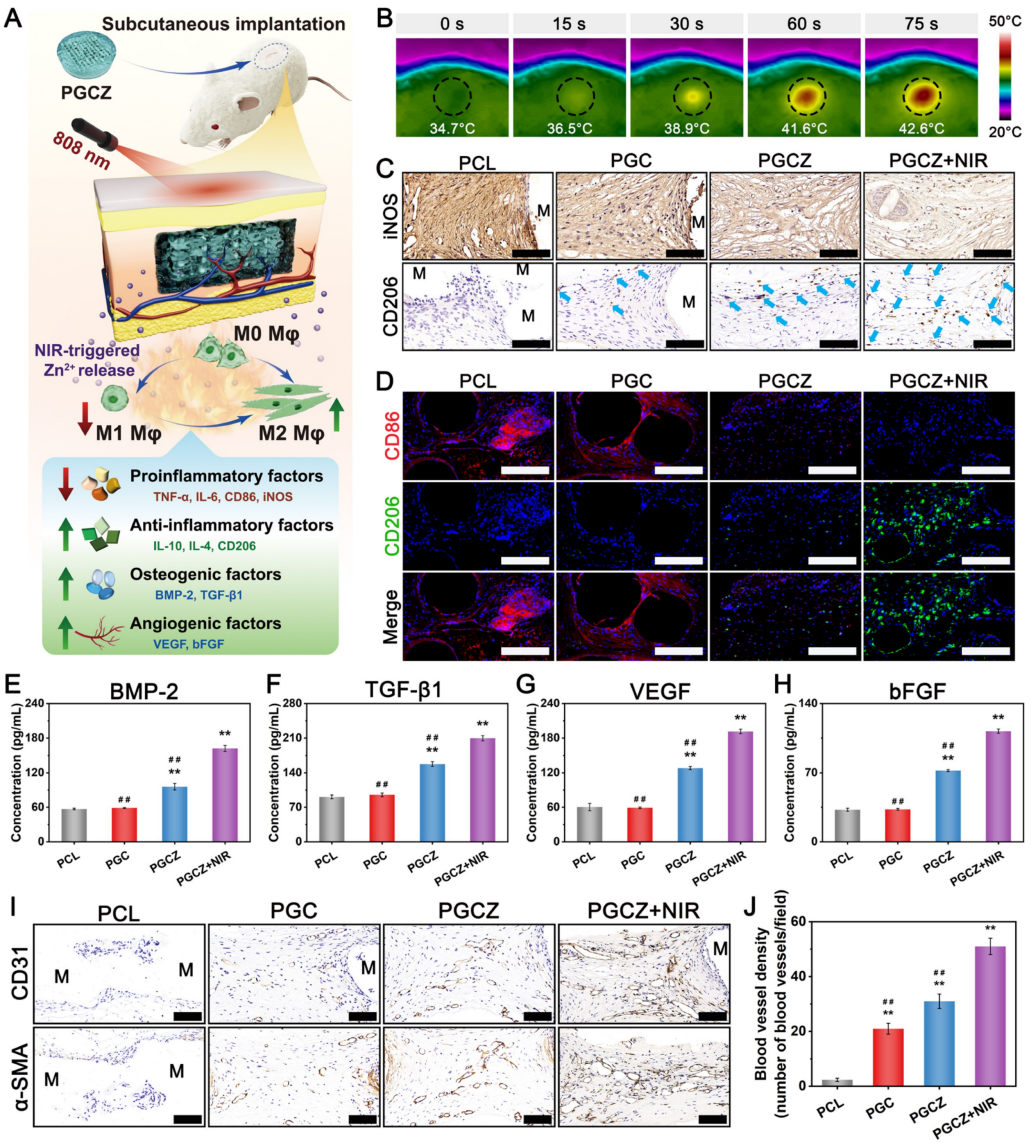
***In vivo* immunomodulatory and angiogenic activities in a rat subcutaneous implantation model. (A)** Schematic diagram of macrophage polarization and vascularization induced by the photoactivated PGCZ hybrid scaffold. **(B)** Infrared thermal images of the implantation site under NIR irradiation (1 W/cm^2^, 808 nm). **(C)** Immunohistochemical staining images of iNOS and CD206 after 7 days of implantation. The blue arrows indicate CD206-positive cells. Scale bar: 100 μm. **(D)** Immunofluorescence staining images of CD86 and CD206 (red: CD86; green: CD206; blue: DAPI). Scale bar: 200 μm. **(E-H)** Secretion of osteogenic (BMP-2 and TGF-β1) and angiogenic (VEGF and bFGF) cytokines induced by the scaffolds *in vivo*. **(I)** Immunohistochemical staining images of CD31 and α-SMA and **(J)** quantitative analysis of the blood vessel density after 2 weeks of implantation. Scale bar: 100 μm. Data are presented as the mean ± SD (n = 3). *P < 0.05 and **P < 0.01 indicate significant differences compared with the PCL group. ^#^P < 0.05 and ^# #^P < 0.01 indicate significant differences compared with the PGCZ+NIR group.

Encouraged by these promising *in vitro* biological experiments, we further evaluated the immunomodulatory and angiogenic capabilities of the photoactivated PGCZ scaffold system using a rat subcutaneous implantation model. After implantation, some of the SD rats in the PGCZ group were treated with 808 nm NIR irradiation every 2 days (**Figure 8A**). During the experiment, the changes in temperature and infrared images were dynamically monitored by an infrared thermal imaging camera. As shown in **Figure 8B**, after irradiation with NIR light for 75 s, the temperature of the implanted PGCZ scaffold reached 42 ± 1 °C, and the scaffold cooled to body temperature within 120 s (**Figure S31**). Furthermore, the photothermal performance of the PGCZ scaffold system was well maintained after four cycles of laser on and off processes, endowing the PGCZ scaffold with potential as a durable photothermal agent for repeated *in vivo* treatment. The ability to repeatedly increase and decrease the temperature allowed for accurate, controlled release of Zn^2+^ while not affecting the biological activity of ZIF-8@PDA or damaging normal cells and tissues.

After 7 days, the results of immunohistochemical staining showed that the PGCZ+NIR group had significantly increased expression of CD206, a representative surface marker of M2 macrophages (**Figure 8C**), revealing favorable anti-inflammatory activity. Correspondingly, a reduction in iNOS expression was observed in the groups treated with the PGCZ scaffold compared to the PCL and PGC groups, and the most substantial decrease was found in the PGCZ scaffold group upon NIR irradiation (**Figure S32**). Similar findings were also observed by immunofluorescence staining and ELISA assays, as illustrated in **Figure 8D** and **Figure S33**.

A substantially higher expression level of CD206 (green) was detected in the PGCZ+NIR group, while a lower expression level of iNOS (red) was observed (**Figure 8D**). Additionally, as displayed in **Figure S33**, NIR-irradiated PGCZ effectively decreased the expression of various proinflammatory cytokines (IL-6 and TNF-α) while markedly increasing the secretion of anti-inflammatory cytokines (IL-4 and IL-10). These cytokines are major pathophysiological factors that affect the occurrence and progression of bone regeneration and repair in the diabetic pathological milieu. In addition, there was no significant difference in the levels of CD4 and CD8 in the serum of the rats for each group (**Figure S34**), suggesting that none of the experimental groups exhibited significant immune-activating or immune-suppressing effects.

Benefiting from mild and appropriate NIR irradiation, the PGCZ scaffold system can scavenge ROS and simultaneously release Zn^2+^ to stimulate M2-type polarization of macrophages as well as downregulate proinflammatory factor expression *in vivo*. Notably, the chemical structure of ZIF-8@PDA can dissociate at low pH, which contributes to the accelerated and sustained release of bioactive Zn^2+^, thereby promoting M2 macrophage polarization and alleviating inflammation progression in the slightly acidic inflammatory milieu. The activation of M2 macrophages in the inflammatory state was demonstrated to be beneficial for reducing local tissue inflammation and remodeling the regenerative microenvironment through the production of various anti-inflammatory and reparative cytokines 79. Consistent with the *in vitro* results, ELISA assay *in vivo* verified that the PGCZ scaffold and NIR-assisted mild hyperthermia had a functional role in the production of osteoinductive and angiogenic cytokines, such as BMP-2, TGF-β1, VEGF, and bFGF (**Fig 8E-H**), recreating a favorable microenvironment for vascularization and tissue regeneration. Numerous studies have shown that Zn-containing biomaterials can modulate macrophage polarization toward the anti-inflammatory and pro-healing M2 phenotype by inhibiting the NF-κB signaling pathway 27. Moreover, recent studies have revealed that NIR-triggered mild hyperthermia can exert immunomodulatory, osteogenic and angiogenic effects by modulating the secretion of macrophage-associated cytokines 6, 80, which further validates our results. These results indicated that under the combined action of sustained Zn^2+^ release and MPTT treatment, the PGCZ platform could promote M2 polarization, increase the expression levels of anti-inflammatory and reparative factors, and achieve favorable therapeutic outcomes for expediting tissue regeneration.

In addition to favorable immunomodulation, angiogenesis is also a vital step in accelerating bone regeneration and remodeling by providing sufficient oxygen, nutrients, and cytokines to the impaired region. During tissue repair, following an early transitional inflammatory phase, the main obstacle to achieve efficient bone healing is revascularization. Consequently, to observe new blood vessel formation within the scaffold, immunohistochemical staining for CD31 and α-SMA was performed after implantation for 2 weeks. As depicted in **Figure 8I**, densely interconnected CD31- and α-SMA-positive protein networks were observed in the PGCZ and PGCZ+NIR groups (especially the latter), indicating enhanced vascularization during the tissue regeneration process. In contrast, only scattered CD31- and α-SMA-positive vessels were found in the PGC and PCL groups. Statistical analysis further confirmed that the number of positive vessels within the scaffold was the highest in the PGCZ+NIR group, followed by the PGCZ and PGC groups (**Figure 8J**). The capillary structure within the NIR-irradiated PGCZ scaffold exhibited obvious positive regions, indicating that the combination of ZIF-8@PDA loading and NIR-triggered MPTT synergistically enhanced the expression of early vascularization markers. We assumed that these results could be explained by the improved regenerative microenvironment caused by the sustained release of Zn^2+^ ions and mild photothermal stimulation after implantation, which led to the activation of M2 macrophages and the secretion of pro-angiogenic cytokines. This conclusion was consistent with the high expression levels of BMP-2, TGF-β1, VEGF, and bFGF detected by ELISA assay. Considering these data together, it was determined that the introduction of the ZIF-8@PDA-loaded hydrogel in the hybrid scaffold system exerted a significant role in immune regulation and vascular regeneration. Meanwhile, periodic mild hyperthermia (42 ± 1 °C) also had a synergistic effect on inducing macrophage polarization from M1 to M2, inhibiting proinflammatory cytokine production, and relieving inflammation. Overall, we believe that our PGCZ system, which integrates various intriguing characteristics of PCL and GMCS/Z, can coordinate well with each other to improve bone repair efficacy, showing immense potential in tissue engineering and biomedical applications.

### *In vivo* evaluation of bone regeneration

**Figure 9:**
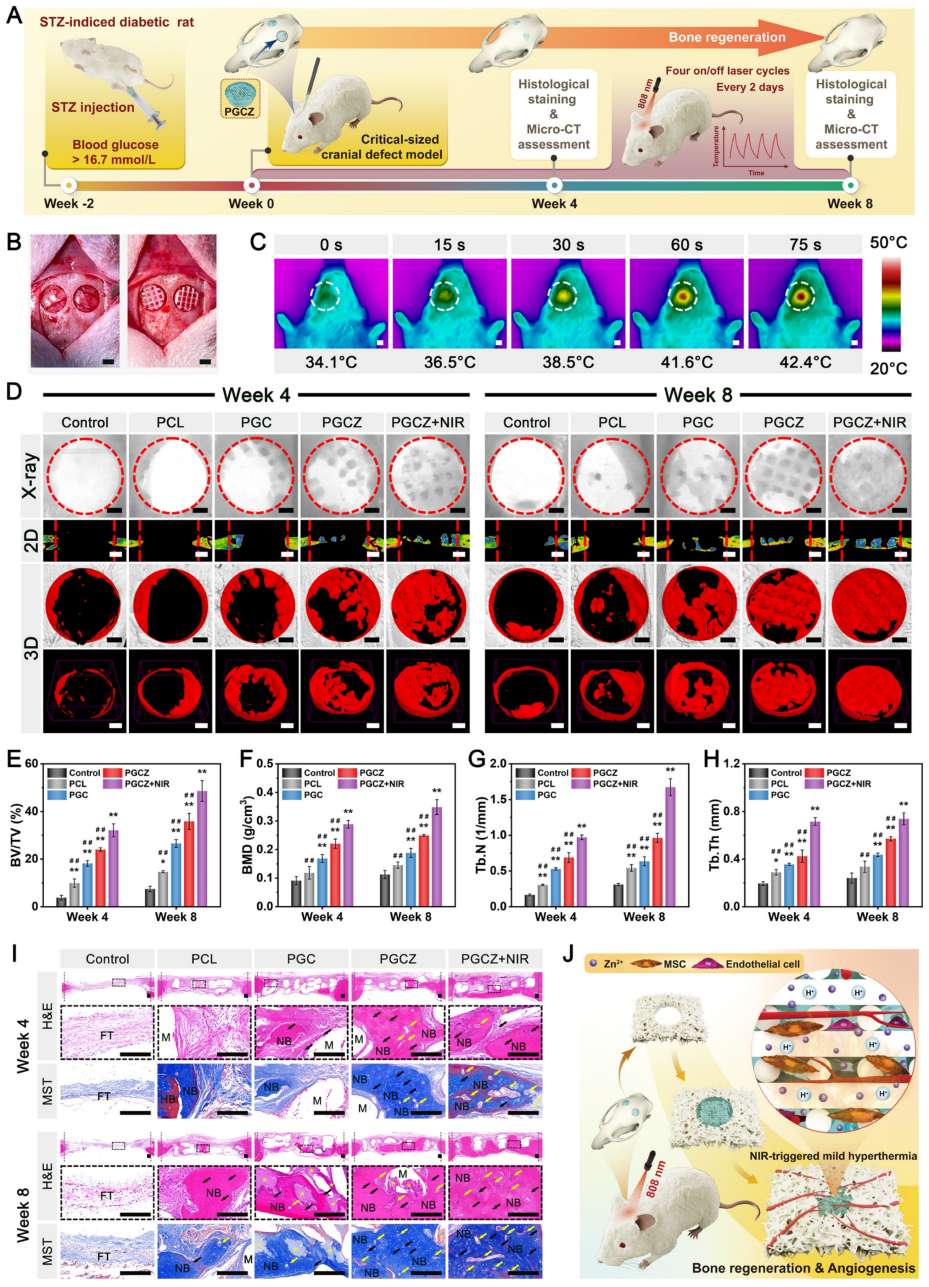
***In vivo* bone repair in a diabetic rat cranial defect model. (A)** Schematic diagram of the *in vivo* treatment procedure of cranial defect regeneration under diabetic conditions. **(B)** Establishment of the calvarial defect model in diabetic rats. Scale bar: 2 mm. **(C)** Infrared thermal images of the implantation site under NIR irradiation (1 W/cm^2^, 808 nm). Scale bar: 5 mm. **(D)** X-ray, 2D, and 3D micro-CT images of the newly formed bone in the defect areas at 4 and 8 weeks after implantation. Scale bar: 1 mm. **(E-H)** Quantitative analysis of bone morphology parameters, including BV/TV, BMD, Tb.N, and Tb.Th. **(I)** H&E staining and MST staining images of decalcified bone tissue. FT: fibrous tissue. HB: host bone. NB: newly formed bone tissue. M: residual PCL materials. The black arrows represent the bone lacunae. The yellow arrows represent the central canal. The yellow asterisks represent the residual hydrogel materials. Scale bar: 200 μm. **(J)** Schematic diagram of bone formation and vascularization in the defect areas under diabetic conditions. Data are presented as the mean ± SD (n = 3). *P < 0.05 and **P < 0.01 indicate significant differences compared with the control group. ^#^P < 0.05 and ^# #^P < 0.01 indicate significant differences compared with the PGCZ+NIR group.

Motivated by the results obtained from the *in vitro* biological experiments and *in vivo* subcutaneous implantation assay, we next examined the *in situ* bone repair ability of the scaffold therapeutic system in a diabetic rat model of cranial defects, as schematically illustrated in **Figure 9A**. After diabetes was successfully induced in rats via streptozotocin (STZ) injection, critical-sized cranial defects (ϕ = 5 mm) were established, followed by scaffold implantation. The application of the scaffold sample in the defect is shown in **Figure 9B**, which could adapt well to the shape of the defect after implantation. During the animal experiment, some of the SD rats in the PGCZ group were subjected to mild NIR irradiation every 2 days, and the temperature at the defect site was kept at 42 ± 1 °C, which was recorded by an infrared thermal imaging camera (**Figure 9C**). With four cycles of irradiation, the photothermal performance of the PGCZ scaffold was well maintained (**Figure S35**), indicating its feasibility for clinical application. At 4 and 8 weeks postoperatively, micro-CT and X-ray images were used to visualize new bone formation in the defect region, where the red region represents the newly formed bone within the defect areas (**Figure 9D**). There was a small amount of new bone formation around the margin of the defect areas in the control group, but varying degrees of regenerated new bone formation were observed in the experimental groups. In addition, there were obvious empty channels within the newly formed bone tissue, which were believed to be the polymer struts of the scaffolds. 3D-reconstructed micro-CT images further revealed that larger amounts of new bone tissue grew into the interconnected pore area in the PGCZ and PGCZ+NIR groups than in the PGC group, whereas minimal new bone formation was observed in the PCL group. It is worth noting that under the action of appropriate NIR irradiation, the PGCZ group exhibited prominent bone repair effects, which led to apparent bridging of the defect at 8 weeks, demonstrating that the PGCZ scaffold with mild hyperthermia had improved bioactivity to induce new bone generation. These findings agreed with the *in vitro* osteogenic differentiation results and further proved that the engineered multifunctional PGCZ scaffold system exhibited satisfactory performance in inducing osteogenesis *in vivo*. Quantitative morphometric analysis based on micro-CT scanning further corroborated that the PGCZ+NIR group had significantly higher BV/TV, BMD, Tb.Th, and Tb.N values than the other groups (**Figure 9E-H**). Among these indices, BV/TV, the ratio of bone tissue volume to total tissue volume, refers to the bone volume fraction, with higher values directly reflecting enhanced formation of new bone 13. These trends were consistent with the results of the *in vitro* experiments, further verifying that the PGCZ scaffold has an excellent capacity to facilitate bone defect repair and healing, with enhanced formation and thickening of new bone tissues. Moreover, no obvious pathological abnormalities or inflammation was observed in the major organs of the rats, including the heart, liver, spleen, lung, and kidney, according to H&E staining (**Figure S36**). These results validated that the combination of the engineered PGCZ scaffold system and NIR-mediated MPTT used in this study accelerated bone healing in a safe way, showing superior therapeutic efficacy in a diabetic rat bone defect model.

Histological staining analysis, including H&E staining and MST staining, further confirmed the micro-CT results. As shown in **Figure 9I**, the white region represents the PCL scaffold, and the pink or dark blue regions represent the newly generated bone tissue. In the control group, the bone defect areas were mainly covered by a large amount of fibrous tissue and showed little new bone formation at 4 and 8 weeks after implantation, implying poor bone regeneration capacity. In sharp contrast, extensive newly formed bone tissue and bony structures were observed in the scaffold group, especially in the PGCZ and PGCZ+NIR groups. At 8 weeks after implantation, the new bone layer within the defect region was more continuous and thicker in the PGCZ group than in the PCL and PGC groups. Upon NIR irradiation, the PGCZ scaffold group exhibited significantly enhanced bone repair capacity, with the highest amount of regenerated bone tissue formed in the defect region, revealing the active bone regeneration process. Surprisingly, numerous mineralized collagen fibers were observed to penetrate within the pores of the PGCZ scaffolds, which was particularly evident after NIR treatment, indicating the ability of the PGCZ scaffold plus mild heat stimulation to promote bone ingrowth and facilitate scaffold-tissue integration. In addition, more newly formed bone lacunae and central canals with blood vessel ingrowth were detected in the defect areas of the PGCZ+NIR group, suggesting the enhanced bone reconstruction and neovascularization capacity of the PGCZ scaffolds under NIR irradiation. Therefore, the photoactivated PGCZ scaffold system showed a better match between material biodegradation and bone regeneration than did the pure PCL and PGC scaffolds. The results of Goldner's trichrome (GST) staining were similar to those of H&E staining and MST staining, further supporting that ZIF-8@PDA loading and NIR irradiation synergistically accelerated new bone formation and maturation.

The maximum amount of mature woven bone tissue (green-stained area) was generated within the defect areas of the PGCZ+NIR group, with continuous bony structure and bone mineralization comparable to those of the surrounding native bone tissue (**Figure S37**). In contrast, there was no significant mature bone formation in the control group, and only a small amount of mature bone formed in the PCL and PGC groups. A schematic diagram of the effect of the PGCZ scaffold on promoting bone regeneration under mild photothermal stimulation is shown in **Figure 9J**. Notably, the local enrichment of acid metabolites induced a weakly acidic environment in diabetic bone defect areas, which is an important prerequisite for guaranteeing the on-demand release of Zn^2+^. Benefiting from its intrinsic pH-responsive characteristics, the PGCZ scaffold could achieve acid-responsive release of encapsulated ZIF-8@PDA, leading to the rapid release of Zn^2+^ in the weakly acidic milieu in the early stage after bone injury. Meanwhile, under periodic NIR irradiation, PGCZ-derived mild hyperthermia could synergize with released Zn^2+^ ions to further expedite bone formation by reversing high oxidative stress, inducing M2 macrophage polarization, and regulating the regenerative microenvironment to facilitate vascularization and osteogenic differentiation. Collectively, these data verified that this multifunctional PGCZ scaffold, designed by the combination of soft and hard concepts under periodic NIR irradiation, displayed the most substantial therapeutic efficacy for augmented bone regeneration under diabetic pathological conditions.

### Mechanisms of diabetic bone healing

According to the results of a series of *in vitro* and *in vivo* biological experiments, as described above, our proposed photoactivated PGCZ scaffold system, which has prominent osteogenic, immunomodulatory, antioxidant, antibacterial, and angiogenic functions, holds promise for the treatment of diabetic bone defects. However, the underlying mechanism by which the PGCZ scaffold platform promotes diabetic bone regeneration remains unclear, limiting its development for bone tissue engineering.

**Figure 10:**
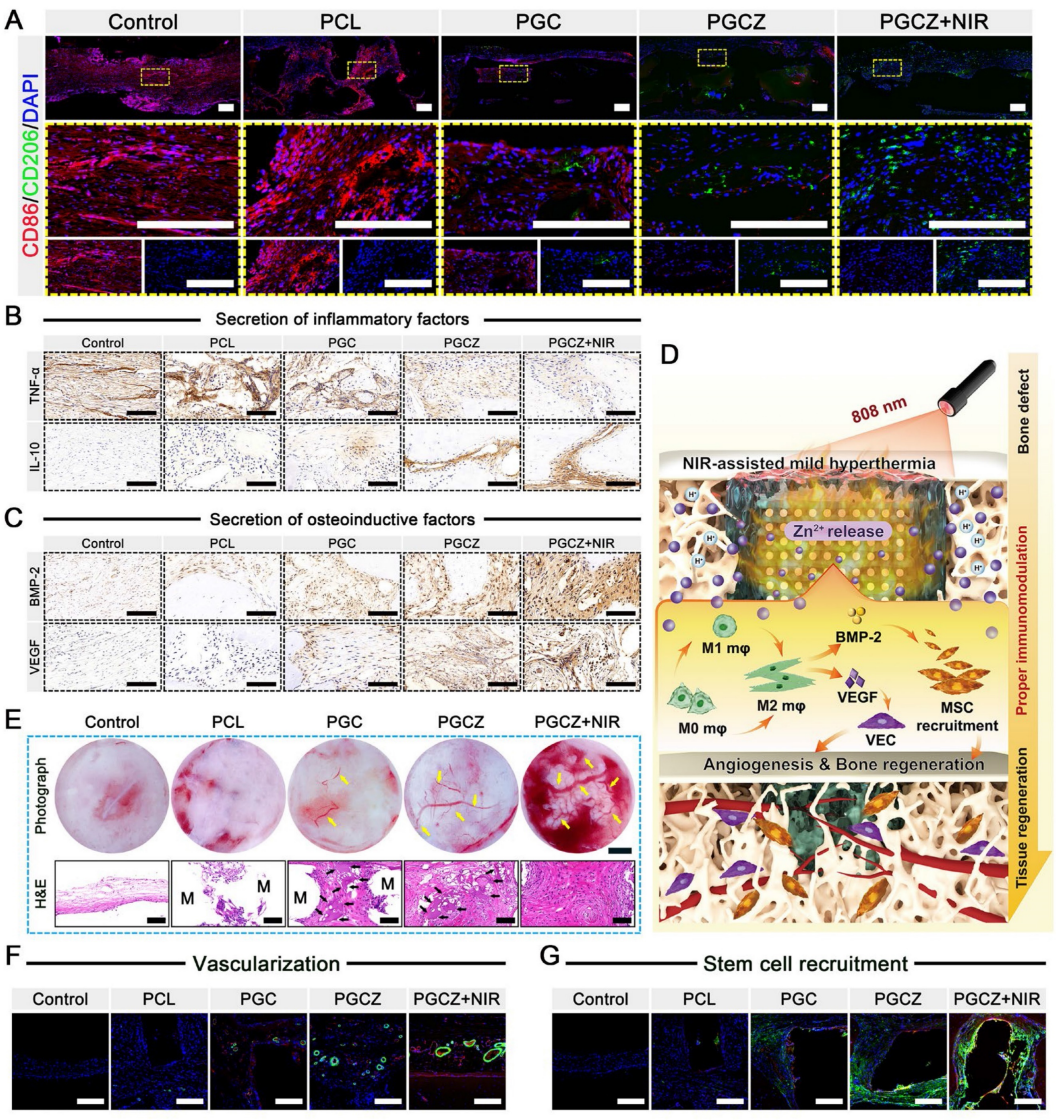
***In vivo* immune regulation, revascularization and endogenous stem cell recruitment during the inflammation and repair stages. (A)** Immunofluorescence staining images of CD86 and CD206 (red: CD86; green: CD206; blue: DAPI) after 2 weeks of implantation. Scale bar: 200 μm. **(B)** Immunohistochemical staining images of TNF-α and IL-10. Scale bar: 100 μm. **(C)** Immunohistochemical staining images of BMP-2 and VEGF. Scale bar: 100 μm. **(D)** Schematic illustration of early immunomodulation and tissue regeneration induced by the photoactivated PGCZ hybrid scaffold. **(E)** H&E staining and macroscopic images of the defect regions after 4 weeks of implantation. Scale bar: 100 μm (H&E staining images) and 1 mm (optical images). The yellow arrows represent the newly formed capillary vessels in the defect areas. The black arrows represent the degradation of the hydrogel phase, accompanied by the infiltration of endogenous cells. **(F)** Immunofluorescence staining images of CD31 and α-SMA (red: CD31; green: α-SMA; blue: DAPI). Scale bar: 200 μm. **(G)** Immunofluorescence staining images of CD44 and CD90 (red: CD44; green: CD90; blue: DAPI). Scale bar: 200 μm. Data are presented as the mean ± SD (n = 3). *P < 0.05 and **P < 0.01 indicate significant differences compared with the control group.^ #^P < 0.05 and ^# #^P < 0.01 indicate significant differences compared with the PGCZ+NIR group.

Considering the significant role of immune regulation in the process of bone healing 71, it is crucial to precisely coordinate and harmonize the interactions between various bone regeneration-related events (e.g., MSC recruitment, vascularization, osteogenic differentiation, and bone mineralization) and the immune response. Following bone injury, the persistent inflammatory expression of M1 macrophages usually causes difficulty in transitioning from the inflammation to the proliferation phase, which is known to be responsible for the prolonged non-healing of diabetic bone defects 71. Effective modulation of the macrophage transition from the proinflammatory M1 phenotype to the anti-inflammatory M2 phenotype is indispensable for the transition from the acute inflammation phase to the proliferation phase, thus restoring the normal immune response and expediting diabetic bone healing 75, 81. The results mentioned above unraveled that the NIR-irradiated PGCZ scaffold system possesses a desirable immunomodulatory effect *in vitro*, which was more effective in suppressing M1 polarization, scavenging ROS while activating macrophage transformation toward the M2 phenotype. Thus, it was reasonable to hypothesize that the enhanced bone repair observed upon implantation of the PGCZ scaffold into cranial defects of diabetic SD rats is related to a differential immune response. To confirm the possible mechanism underlying the therapeutic efficacy of the photothermal PGCZ scaffold system, its macrophage polarization and anti-inflammatory effects were investigated *in vivo*. Immunofluorescence staining and flow cytometry analysis were performed to determine the polarization status of the macrophages. As displayed in **Figure 10A** and **Figure S38A-C**, the population of M1 phenotype macrophages (CD86^+^) was substantially increased in both the control and PCL groups at 2 weeks, suggesting that diabetes and bone injury resulted in a severe acute inflammatory response. This phenomenon was mainly attributed to the activation of the NF-κB signaling pathway under diabetic conditions 82, which induces macrophage polarization toward the M1 type, thereby leading to the continuous expression of ROS and proinflammatory cytokines. This was further evidenced by the remarkably elevated level of inflammatory cytokines (TNF-α), as depicted in **Figure 10B**. In contrast, the population of M2 phenotype macrophages (CD206^+^) in the PGCZ group was significantly higher than that in the other groups, which was consistent with the conclusions of *in vitro* studies. Correspondingly, a reduction in CD86 expression was observed in the groups treated with PGC and PGCZ scaffolds. Notably, this effect was particularly obvious in the PGCZ+NIR group, which exhibited the lowest CD86^+^ and highest CD206^+^ expression among all the groups, confirming its potent ability to promote the phenotypic transition of macrophages from M1 to M2. Furthermore, to assess the secreted cytokines involved in macrophage polarization, the mRNA levels of M1 proinflammatory markers (*IL-6*, *TNF-α*, *iNOS*, and *CD86*) and M2 anti-inflammatory markers (*IL-4*, *IL-10*, *Arg-1*, and *CD206*) were detected at 2 weeks using qRT-PCR analysis. As shown in **Figure S38D-E**, gene expression associated with an M1 phenotype was downregulated, while gene expression related to an M2 phenotype was upregulated in both the PGCZ and PGCZ+NIR groups compared to all other groups. Notably, the PGCZ+NIR group exhibited the lowest expression of M1 phenotype markers and the highest expression of M2 phenotype markers, corroborating with the aforementioned *in vitro* and *in vivo* results that PGCZ+NIR group favored M2 polarization of macrophages. It is widely known that M2 macrophages feature high expression of CD206 and secretion of anti-inflammatory cytokines, such as IL-10, Arg-1, and TGF-β1, which play an indispensable role in inflammation regression and tissue repair 1. The ability of the PGCZ+NIR system to regulate the polarization of macrophages and reduce inflammatory factor levels was also proven by immunohistochemical staining, in which the production of the proinflammatory cytokine TNF-α was reduced and the anti-inflammatory cytokine IL-10 was increased after treatment with PGCZ+NIR compared to all the other groups (**Figure 10B**). IL-10 expression in the PGCZ group was higher than that in the PGC, PCL, and control groups, and its expression was significantly increased through NIR stimulation. In contrast, intense inflammation was observed in the control and PCL groups, in which the highest expression level of TNF-α was observed (**Figure S39A**). These findings showed that the PGCZ scaffold combined with mild photothermal treatment synergistically promoted M2 macrophage polarization and inhibited the local “cytokine storm”, which alleviated incipient inflammation and oxidative damage, and accelerated the transition of the bone healing phase from inflammation to repair and remodeling.

According to previous reports, activated M2 macrophages not only secrete anti-inflammatory cytokines (such as IL-10) to reduce inflammation at the early stage after implantation but also release large amounts of tissue growth factors, such as BMP-2 and VEGF, which can facilitate osteogenesis and angiogenesis *in situ* 77. Consequently, to further elucidate the mechanism of diabetic bone healing induced by PGCZ+NIR, immunohistochemical staining for BMP-2 and VEGF was conducted at 2 weeks. As shown in **Figure 10C**, the PGCZ group had higher BMP-2 and VEGF expression levels than did the PGC, PCL, and control groups. In particular, the NIR stimulation-derived MPTT increased the positive area (brown) of BMP-2 and VEGF in the PGCZ group (**Figure S39B**), indicating enhanced osteogenic and angiogenic potential.

Considering the slightly acidic inflammatory microenvironment, the PGCZ scaffold system with dual NIR/pH responsiveness can significantly accelerate the release of Zn^2+^ ions to modulate the local immune response and reduce inflammation in diabetic pathological milieus under the action of on-demand NIR irradiation. Accordingly, the stimuli-responsive release of Zn^2+^ ions and mild photothermal stimulation might be the primary reasons for the activation of M2 macrophages and the corresponding favorable immune microenvironment provided by the PGCZ scaffold system. This conclusion was consistent with the results of *in vitro* immunomodulatory activity, and further verified that the designed PGCZ photothermal scaffold was expected to break this vicious cycle, reduce the acute inflammation period, and promote bone healing by producing a beneficial pro-healing immune microenvironment. The potential mechanism proposed above is schematically illustrated in **Figure 10D**.

After ameliorating inflammation by polarizing macrophages to the M2 phenotype, the subsequent tissue repair phase involving MSC recruitment and neovascularization is activated. In particular, the establishment of functional blood vessel networks is a critical step in the reconstruction process during bone healing, which can play a beneficial synergistic effect on cell recruitment, tissue ingrowth, cytokine secretion and transportation, bone mineralization, and vascularized bone regeneration 11. At 4 weeks after implantation, gross observation and H&E staining revealed abundant host cell aggregation and apparent capillary vessel formation in the defect treated with PGCZ+NIR (**Figure 10E**), indicating that the PGCZ scaffold combined with on-demand NIR irradiation recreated an optimized regenerative microenvironment to initiate endogenous cell recruitment and angiogenesis. Meanwhile, along with cell infiltration, the hydrogel materials gradually degraded, which provided sufficient space for subsequent vascular formation and new bone ingrowth. We then performed immunofluorescence staining of CD44/CD90 and CD31/α-SMA to testify whether MSC recruitment and the angiogenic response were activated after hydrogel treatment. As depicted in **Figure 10F-G**, the expression levels of CD44^+^/CD90^+^ cells (specific antigens of stem cells) and CD31^+^/α-SMA^+^ blood vessels were substantially higher in the PGCZ+NIR group than in the PGCZ and PGC groups, whereas they were significantly lower in the control and PCL groups. With the introduction of GMCS and ZIF-8@PDA, PGCZ could efficiently stimulate early angiogenesis, induce the formation of functional capillaries, and simultaneously drive the migration of endogenous MSCs to the bone defect site under mild NIR irradiation. This trend is identical to the data of *in vitro* biological experiments, providing further evidence that PGCZ in combination with mild hyperthermia has great potential to promote angiogenesis and stem cell recruitment. Additionally, the paracrine signaling mediated by the pro-healing factors expressed by M2 macrophages (e.g., BMP-2 and VEGF) is also responsible for the enhancement of *in situ* stem cell recruitment and angiogenic differentiation. According to the previous literature, M2 macrophages can synergistically promote osteogenesis and vascularization through regulating multiple key factors and signaling pathways, thereby promoting new bone regeneration in a highly hyperglycemic inflammatory microenvironment 83, 84. Our findings are consistent with the abovementioned reports. Overall, these results indicated that the PGCZ scaffold combined with mild photothermal stimulation exerted a favorable impact on the inflammatory phase of diabetic bone healing, which was conducive to its transition to the proliferative phase, laying a fundamental foundation for subsequent bone regeneration and reconstruction.

**Figure 11:**
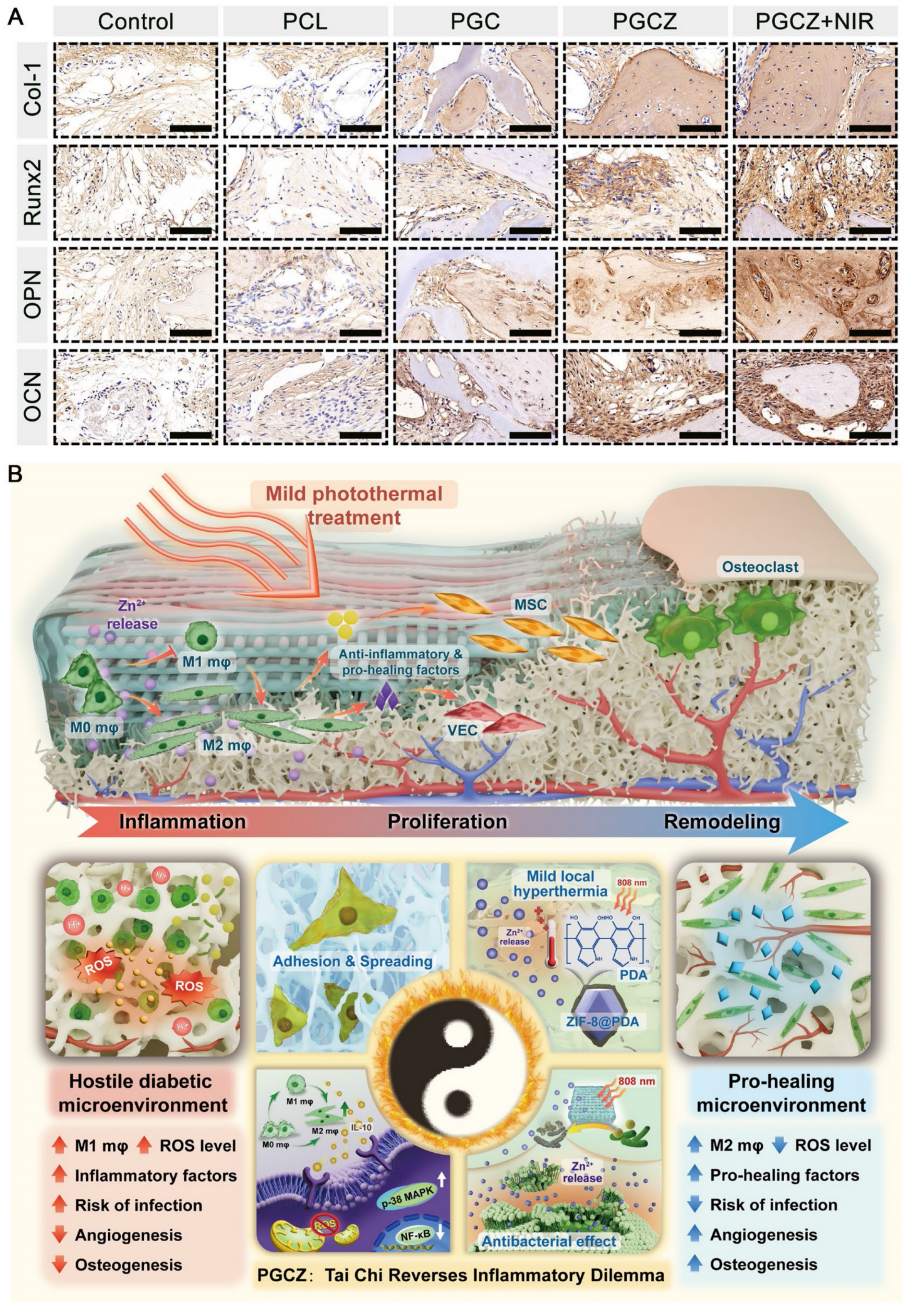
***In vivo* bone mineralization and resorption during the remodeling stage. (A)** Immunohistochemical staining images of Col-1, Runx2, OPN, and OCN. Scale bar: 100 μm. **(B)** Schematic illustration of the mechanism of the photoactivated PGCZ scaffold for accelerated diabetic bone healing.

During the mineralization and remodeling phases, bone-specific ECM deposition is a key factor that determines bone maturation in the later stage and is mainly mediated by osteoblasts. Col-1, Runx2, OPN, and OCN are well-known factors involved in regulating ECM mineralization and bone tissue maturity during the process of bone regeneration 3. From the results of the immunohistochemical staining analysis, the PGCZ group showed significantly upregulated expression of Col-1, Runx2, OPN, and OCN compared to that in the PGC group at 8 weeks, whereas no significant expression was observed in the PCL and control groups (**Figure 11A**). More remarkably, the expression of these four osteogenic proteins in the PGCZ group substantially increased under periodic NIR stimulation, revealing enhanced osteoinductive capability. Further quantitative analysis of immunohistochemical staining showed that the NIR-irradiated PGCZ group had the best promotion effect on osteogenic differentiation and bone mineralization (**Figure S40**), which was also consistent with the gene expression level *in vitro*. Except for promoting osteoblast differentiation and inducing ECM mineralization, osteoclast-mediated bone resorption also plays a distinct role in the process of bone remodeling 13. When osteoclast-mediated bone resorption exceeds osteoblast-mediated bone formation, bone repair is impeded, leading to delayed tissue healing. As demonstrated in the previous section, the prepared PGCZ scaffold could remarkably promote new bone formation and matrix mineralization during bone healing; therefore, it is also crucial to evaluate the effect of the photothermal therapeutic platform on osteoclast differentiation. Tartrate-resistant acid phosphatase (TRAP), a critical marker for osteoclast phenotype, is synthesized by osteoclast-like cells and can be recognized as a reliable biomarker for osteoclast differentiation associated with bone resorption and remodeling 85. As seen from TRAP staining in **Figure S41**, the number of osteoclasts (dark purple) near the bone defect margins in the control and PCL groups was significantly higher than that in the other groups, illustrating osteoclast activation, which was related to the excessive accumulation of M1 macrophages, ROS, and proinflammatory factors such as IL-6 and TNF-α in the diabetic inflammatory microenvironment 4. Notably, this phenomenon was obviously reversed after the utilization of the PGCZ scaffold, which can even be further reduced with NIR irradiation, confirming that PGCZ combined with NIR irradiation effectively inhibited osteoclast differentiation and activity in the bone remodeling phase. During the final process of bone repair, osteoclasts resorb woven bone, while osteoblasts replace it with lamellar bone. Many studies have suggested that Zn^2+^ ions exert dual functions of inducing osteogenesis and mineralization, as well as inhibiting osteoclast activity by modulating the NF-κB signaling pathway 26. Based on the above results and related studies, it could be speculated that Zn^2+^ ion release, which is controlled by pH and photothermal stimuli, is favorable for inducing osteoblast differentiation and calcium deposition as well as inhibiting osteoclast differentiation, thereby expediting bone remodeling and mineralization.

Herein, the excellent performance of the PGCZ scaffold in promoting diabetic bone defect healing could be mainly attributed to the following reasons (**Figure 11B**): 1) The prepared PGCZ scaffold with adequate mechanical support and highly biomimetic 3D natural ECM provided a 3D physical scaffold for cell adhesion, growth, migration, spreading, and differentiation. 2) After implantation *in vivo*, the photothermal PGCZ scaffold system triggered Zn^2+^ release via thermal stimulation under NIR irradiation, efficiently eliminated excessive ROS, polarized macrophages toward the pro-regenerative M2 phenotype and cooperated with osteoblasts and endothelial cells for subsequent bone tissue reconstruction and regeneration. 3) The combined impact of Zn^2+^ release, which fosters immunomodulation and osteogenesis while inhibiting osteoclastogenesis, and mild heat stimulation, generates a microenvironment conducive to converting the inflammation phase into the proliferation phase, thereby stimulating neovascularization, stem cell recruitment, and osteogenesis as well as suppressing the osteoclastic resorption process at the bone defect site, ultimately accelerating diabetic bone defect healing. Furthermore, IL-10 secreted by M2 macrophages has been recognized for its ability to inhibit osteoclast differentiation in the early stage of osteoclast formation. IL-10 can also form an immune microenvironment favorable for osteogenic differentiation and bone mineralization by activating the P38 MAPK pathway and inhibiting the NF-κB pathway 86. Remarkably, our PGCZ scaffold system successfully upregulated IL-10 expression, thereby reducing its impact on osteoclastogenesis and promoting bone matrix deposition during tissue remodeling. 4) Upon NIR irradiation, the mild photothermal effect improved the bacterial sensitivity to antibacterial Zn^2+^ ions, which endowed the scaffold with effective antibacterial activity and provided a viable strategy to effectively prevent bacterial infection and reduce the risk of becoming multi-drug-resistant bacteria. The synergistic cascade of the above beneficial factors actively matches the complex process of bone repair *in vivo* under diabetic conditions, leading to a pro-restorative microenvironment and ultimately achieving high-performance bone regeneration, much like “Tai Chi reverses inflammatory dilemma”. Overall, the results above provide compelling evidence that the photoactivated PGCZ scaffold system we propose could accelerate bone healing by activating M2 polarization, inhibiting inflammation, promoting angiogenesis and osteogenic differentiation, and suppressing osteoclast activity, offering new avenues to optimize the regenerative microenvironment and enhance bone regeneration processes, especially in the context of diabetic conditions.

## Conclusion

In summary, we successfully engineered an intelligent photoactivated soft-hard combined system (PGCZ) with integrated immune regulation, bacterial eradication, revascularization, and bone regeneration based on ZIF-8@PDA nanosystem-mediated functionalization for enhanced healing of diabetic bone defects. The multifunctional PGCZ scaffold system was designed by *in situ* integration of the ZIF-8@PDA-loaded double-network hydrogel (GMCS/Z) into a 3D-printed PCL scaffold. 3D-printed PCL provides effective and long-term mechanical support, and the incorporation of ZIF-8@PDA into the hydrogel provides chemically reactive and bioadhesive sites and improves the interfacial integration between the hydrogel and PCL phases. Owing to the perfect combination of structural and functional properties, the engineered PGCZ composite scaffold not only presented superior physical and photothermal properties, but also recreated a conducive regenerative microenvironment through mild photothermal effects and the on-demand release of Zn^2+^. *In vitro* biological experiments verified that the PGCZ scaffold system exhibited outstanding cytocompatibility, ROS scavenging and antibacterial activities, and osteogenic capacity upon appropriate NIR irradiation as a result of synergistic mild hyperthermia, hydrogel functionalization, and Zn^2+^ leaching.

Moreover, the NIR-assisted PGCZ scaffold system potently reversed the adverse effects of the proinflammatory microenvironment and reprogrammed dysfunctional macrophages into the pro-regenerative M2 phenotype simultaneously, producing a cascade of anti-inflammatory and pro-healing cytokines for enhanced angiogenesis and osteogenesis. Thanks to these features, after implantation of our PGCZ in a critical-sized bone defect model of diabetic rats, it could shorten the inflammation period and activate the polarization of macrophages to the M2 phenotype, leading to accelerated vascular regeneration and endogenous stem cell recruitment at the early inflammatory stage and inducing the inflammatory phase transition to subsequent proliferation and remodeling stages, which ultimately promoted bone repair and healing in the diabetic microenvironment. Therefore, such a photoactivated soft-hard combined scaffold system features the integrated functions of mild hyperthermia, antioxidant, antibacterial and immunomodulatory properties, as well as angiogenesis and osteogenesis promotion, which provides an efficient multifunctional strategy for the individual treatment of diabetic bone defects and can be extended to applications for other bone injuries, such as neoplastic bone defects and infected bone defects, showing high clinical translation potential.

## Materials and Methods

### Materials

Gelatin (strength of the gelatin, approximately 100 g Bloom), carboxymethyl chitosan (CMCM, molecular weight (MW): 50000-190000 Da, 98%), PCL, methacrylic anhydride, lithium phenyl (2,4,6-trimethylbenzoyl) phosphinate (LAP), dopamine hydrochloride (DA), lipopolysaccharide (LPS), and tris (hydroxymethyl) aminomethane (Tris) were provided by Sigma-Aldrich Trading Co., Ltd. (Shanghai, China). Zinc nitrate hexahydrate (Zn(NO_3_)_2_·6H_2_O) was obtained from Shanghai Youshi Chemical Co., Ltd. (Shanghai, China). Fetal bovine serum (FBS), high glucose Dulbecco's modified Eagle's medium (DMEM), phosphate-buffered saline (PBS), trypsin-EDTA, and penicillin/streptomycin (P/S) were purchased from Gibco Life Technologies Co. (Grand Island, USA) for cell culture *in vitro*. Mouse calvaria-derived MC3T3-E1 preosteoblastic cells, human umbilical vein endothelial cells (HUVECs) and macrophages (RAW264.7 cells) were supplied by the Institute of Biochemistry and Cell Biology of the Chinese Academy of Sciences (Shanghai, China). Cell counting kit-8 (CCK-8) was acquired from Dojindo Laboratories (Kumamoto, Japan). *Staphylococcus aureus* (*S. aureus*, ATCC 6538) and *Escherichia coli* (*E. coli*, ATCC 25922) were obtained from Guangdong Microbial Culture Collection Center. The live/dead cell staining kit was purchased from BestBio Biotechnologies (Shanghai, China). Triton X-100 (Sigma-Aldrich), DAPI (Sigma-Aldrich), and TRITC-labeled phalloidin (Invitrogen) were used for cell staining. TRIzol RNA extract kit, radioimmunoprecipitation assay (RIPA) lysis buffer, 5-bromo-4-chloro-3-indolyl phosphate/nitro blue tetrazolium (BCIP/NBT) Alkaline Phosphatase (ALP) Color Development Kit, 2′,7′-dichlorofluorescein diacetate (DCFH-DA) probe, and bicinchoninic acid (BCA) protein assay kit were purchased from Beyotime Biotechnology Co., Ltd. (Jiangsu, China). The ALP assay kit was provided by Jiancheng Biotech Institute (Nanjing, China). The live/dead bacterial staining kit, alizarin red S (ARS) solution, and Von Kossa solution were acquired from Solarbio Co., Ltd. (Beijing, China). All chemicals were used without further purification. The water applied in all experiments was purified by a Milli-Q cycle purification system (Millipore, USA).

### Preparation and characterization of GelMA and ZIF-8@PDA

In this work, GelMA was chemically synthesized following a previous report 87. Briefly, 100 mL of gelatin solution (10%, w/v) was obtained by dissolving gelatin powder in PBS (pH = 7.4) under stirring conditions. Then, 8.0 mL of methacrylic anhydride was added dropwise to the above gelatin solution at 50 °C and stirred for 4 h. To remove unreacted methacrylic anhydride and any byproducts, the above mixed solution was poured into a dialysis bag (cutoff molecular weight 3500) and purified at 40 °C for 7 days. The final GelMA product was obtained by lyophilization and stored for further use. The synthesis of GelMA was confirmed by using proton-1 nuclear magnetic resonance (^1^H-NMR, AV500 MHz, Bruker, Switzerland) and Fourier transform infrared spectroscopy (FTIR; Thermo Scientific Nicolet iN10, USA).

PDA-modified ZIF-8 (ZIF-8@PDA) was synthesized based on a previously reported method with minor modifications 4. Briefly, 200 mg of Zn(NO_3_)_2_·6H_2_O was completely dissolved in 2.8 mL of deionized (DI) water, and the mixture was stirred for 20 min. Then, 2 g of 2-methylimidazole (2-MIM) was dissolved in 8 mL of DI water to form a homogeneous solution, which was quickly added to the above mixture and allowed to react at room temperature for 24 h. Subsequently, the synthesized white ZIF-8 nanoparticles were collected by centrifugation and washed with DI water three times. ZIF-8@PDA was prepared by the self-polymerization of dopamine as a coating layer on ZIF-8, as previously described 38. For PDA modification, the collected ZIF-8 nanoparticles (1 g) were uniformly dispersed in 200 mL of Tris-HCl solution (10 mM, pH = 8.5) containing dopamine (400 mg), which was stirred for 12 h in the dark. Finally, the mixture was washed, centrifuged, and freeze-dried to obtain black ZIF-8@PDA nanoparticles.

The morphology and elemental distribution of the synthesized nanomaterials were observed using field emission scanning electron microscopy (FE-SEM, Zeiss, SIGMA, Germany) and transmission electron microscopy (TEM, JEM2100, Hitachi, Tokyo, Japan) equipped with energy-dispersive spectroscopy (EDS). The particle size distribution, polydispersity index (PDI), and ζ-potential of the synthesized nanomaterials were measured by a dynamic light scattering system (DLS, Zetasizer Nano Series, Malvern Instruments Ltd., Malvern, UK). The phase composition and chemical bond structure of the samples were analyzed by X-ray diffraction (XRD, D8A A25 X, Bruker, Germany), FTIR, and Raman spectroscopy (LabRAM HR800 Horiba, JobinYvon, France). The elemental composition and valence state were analyzed by X-ray photoelectron spectroscopy (XPS, ESCALAB 250XI, Thermo Scientific, New York). The ultraviolet-visible-near-infrared (UV-vis-NIR) absorption spectra were detected by a UV-vis spectrophotometer (UV-2600i, Shimadzu, Japan). The degree of PDA deposition was characterized by thermogravimetric (TG) analysis (Diamond TG/DTA; PerkinElmer Instruments, Shanghai, China) at a heating rate of 10°/min under a nitrogen flow of 100 mL/min.

### Preparation of 3D-printed PCL scaffolds

In this study, 3D-printed PCL scaffolds were fabricated using a Biomaker desktop bio3D printer made by SunP Biotech (Beijing). Briefly, PCL raw material (Mw: 80 kDa) was melted at 120 °C in a printing chamber and then sprayed with a metal nozzle along lines 0, 0, 90, and 90, which were deposited on a receiving platform. Afterward, the samples were cut from the printed scaffold using a puncher with a diameter of 5 mm and used in subsequent experiments.

### Fabrication and characterization of hybrid scaffolds

The ZIF-8@PDA-loaded hybrid hydrogel (GMCS/Z) was prepared by the following method. First, a certain amount of ZIF-8@PDA nanoparticles was dispersed in DI water (10 mL) by sonication and stirring. Then, GelMA (0.7 g) and CMCS (0.3 g) powders were added to the above solution containing the photoinitiator LAP (0.3%, w/v) at 50 °C and stirred to generate a uniform suspension (GMCS/Z precursor solution). Here, we prepared four groups of GMCS/Z hydrogel precursor solutions, namely, GMCS, GMCS/Z1, GMCS/Z2, and GMCS/Z3, with weight ratios of ZIF-8@PDA to GelMA/CMCS of 0%, 2.5%, 5%, and 7.5%, respectively. The chemical structure was examined by XRD, FTIR, and XPS. The thermal stability was characterized by TG analysis.

To prepare the composite scaffold of PGCZ, pure 3D-printed PCL scaffolds were washed with DI water three times and immersed in the prepared hydrogel precursor solution. Afterwards, they were placed under vacuum to ensure that the hydrogel precursor solution infiltrated into the porous skeletons of the PCL scaffolds. After 10 min, the pressure was released. The above process was repeated three times to allow the hydrogel solution to fully infiltrate the PCL framework. Finally, the PCL scaffold filled with GMCS/Z precursor solution was removed and exposed to 405 nm UV light to establish crosslinking. Under the action of UV irradiation and Zn^2+^, the hydrogel/scaffold composite was polymerized to form PGCZ scaffolds. Additionally, a hydrogel precursor solution containing only GMCS was used to prepare the composite hydrogel scaffold by the same method described above, which was designated PGC. The pure 3D-printed PCL scaffold without any modification was designated PCL. Before both *in vitro* and *in vivo* biological experiments, all the samples were first immersed in 75% (v/v) ethanol solution for 24 h, followed by sterilization with UV for 2 h on each side.

After completion of the crosslinking reaction, the gross morphologies of the prepared samples were observed using a digital camera. The surface morphology and microstructure of the freeze-dried hydrogels were examined using FE-SEM and microcomputed tomography (micro-CT; SkyScan 1276, Bruker, Germany). The elemental distribution and chemical composition of the prepared scaffolds were assessed by EDS (UltimMax 40, Oxford, UK). The mean pore size was calculated by ImageJ software. The porosity of the scaffolds was measured based on the ethanol displacement method, as previously described 39. Briefly, porosity (%) = (W1-W0)/ρV0 × 100%, where W0 refers to the initial weight of the samples, W1 refers to the total weight of the samples after immersion in ethanol for 24 h, ρ is the density of ethanol at room temperature, and V0 is the initial volume of the samples. The hydrophilicity of the different scaffolds in terms of the water contact angle was investigated using a contact angle meter (SL200B, Solon Technology Science, Shanghai, China) at room temperature. To measure the mineralization ability *in vitro*, all the samples were soaked in modified simulated body fluid (10 × SBF) at 37 °C for 24 h^88^. To investigate the *in vitro* degradation behavior of the samples, the lyophilized scaffold samples were soaked in 0.05% collagenase (Sigma‒Aldrich)-containing PBS solution at 37 °C (with an enzyme activity of approximately 100 U/mL) on a shaker at 37 °C. The mass remaining (%) = M1/M0 × 100%, where M0 refers to the initial weight of the freeze-dried samples and M1 refers to the total weight of the dried samples at predetermined intervals after immersion. Additionally, SEM was used to observe the lyophilized morphologies of the samples at 28 days after degradation.

### Mechanical performance

The rheological properties of the hydrogel samples were measured using a rheometer (Kinexus, Malvern) under an oscillation-frequency sweep model. To obtain the relevant storage modulus of the hydrogel samples (G') and loss modulus (G''), the samples were prepared into suitable sizes and placed on parallel plates, which was carried out under frequencies of 0.1 to 20 Hz with a constant strain of 0.5% at room temperature. For compression analysis, the samples were tested by a universal testing machine (CMT6503, Shenzhen SANS Test Machine, China) with a compression rate of 1 mm/min.

### Detection of photothermal properties and stimuli-responsive Zn^2+^ release

To evaluate the photothermal properties of the prepared nanoparticles, a ZIF-8@PDA aqueous solution (1 mg/mL) was continuously irradiated with an 808 nm NIR laser (KS-810F-8000, Kai Site Electronic Technology, China) at a power density of 1 W/cm^2^. During irradiation, the heat distribution and temperature changes were recorded with an infrared thermal imager (FLIR Systems, Inc., Wilsonville, OR) at different time intervals. PBS solution with or without ZIF-8 was used as a control in this experiment. Following a similar procedure, the photothermal performances of the hybrid scaffolds were also tested. The photothermal stability of the samples was tested at 1 W/cm^2^ by turning the NIR laser on and off.

To investigate the effects of pH and NIR irradiation on bioactive ion release, lyophilized scaffold samples with a height of 2 mm and diameter of 5 mm were immersed in 10 mL of PBS solution (pH = 6.5 or 7.4) at 37 °C. One group was exposed to daily periodic 808 nm NIR irradiation at 1 W/cm^2^ for 75 s, followed by four heating-cooling cycles, while the other group was not exposed to NIR irradiation. An infrared thermal imager was used to monitor the change in temperature, ensuring that the temperature reached 42 ± 1 °C. When the scaffold was cooled to ambient temperature, the supernatant was collected, and then an equal amount of fresh PBS was added to the above medium. Inductively coupled plasma atomic emission spectroscopy (ICP-AES, Varian 715 ES, California, USA) was used to examine the Zn^2+^ concentration in the leaching solution at different time intervals.

### Screening of the optimal GMCS/Z precursor hydrogel

MC3T3-E1 preosteoblastic cells and RAW264.7 cells were selected and cultured in high-glucose DMEM supplemented with 10% FBS and 1% (v/v) P/S at 37 °C in a humidified atmosphere of 5% CO_2_. The culture medium was refreshed every 2 days, and the cells were passaged upon reaching 80% confluence. To explore the cytotoxicity of various hydrogels, both types of cells were cocultured with hydrogel samples and immersed in cell culture medium. After 1, 2, and 3 days of incubation, cell proliferation was quantitatively evaluated using a CCK-8 assay according to the manufacturer's protocol. The corresponding absorbance was measured at 450 nm by a microplate reader (Multiskanfc, Thermo Scientific). Then, the cells cocultured with different samples were stained with a calcein-AM/EthD-1 double stain kit and an Annexin V-FITC apoptosis detection kit according to the instructions on day 3. Fluorescence images showing the live/dead cells on different hydrogels were acquired with a confocal laser scanning microscope (CLSM; TCS SP8, Leica, Germany). Moreover, cell apoptosis was detected by flow cytometry (FC500, Beckman Coulter, Fullerton, CA, USA), and the data were analyzed with FlowJo software.

For *in vivo* detection of bone formation, a critical-sized rat cranial defect model was established following a published protocol 89. All animal experimental procedures were conducted under supervision and approved by the Animal Care and Use Committee of Wuhan University, and the protocols were in accordance with the Guide for the Care and Use of Laboratory Animals. Briefly, fifteen male Sprague‒Dawley (SD) rats (8 weeks old, 230-250 g, n = 3 for each group) were used for *in vivo* study and anesthetized by inhalation of isoflurane, followed by an incision of the skin and periosteum to expose the skull. Then, two full-thickness defects with a 5 mm diameter on both sides of the skull were created using a trephine drill with continuous irrigation. After hydrogel implantation in the defects and disinfection with iodophor, the incision was carefully closed layer-by-layer with 4-0 nylon sutures. The defects without any treatment composed the control group. At 6 weeks after implantation, the rats were sacrificed, and the harvested calvarial samples were processed for micro-CT analysis. Afterwards, the samples were fixed in 4% paraformaldehyde, decalcified, dehydrated in a graded ethanol series, embedded in paraffin, and prepared into 5 μm-thick sections for histological and immunohistochemical staining. Histological analyses, including hematoxylin and eosin (H&E) staining and Masson's trichrome (MST) staining, were conducted on the sections. Simultaneously, immunohistochemical staining of CD90, Runx2, and OPN was conducted to examine *in situ* MSC recruitment and osteoinductive activity in the defect area after implantation.

### Cytocompatibility and osteogenesis of the hybrid scaffolds

MC3T3-E1 cells were seeded on different sterilized samples and cocultured in an incubator (37 °C, 5% CO_2_). In addition, cells cultured on the PGCZ scaffold were subjected to periodic NIR irradiation (1 W/cm^2^, 808 nm) for 75 s while ensuring that the peak irradiation temperature was maintained at 42 ± 1 °C. When the temperature cooled to room temperature, the NIR light source was applied again. This “on-off” cyclic heating process was continuously conducted four times and recorded by a thermal imaging system. The intermittent and continuous NIR irradiation described above was applied every 2 days. The cell viability of the PCL, PGC, PGCZ, and PGCZ+NIR groups was determined by a CCK-8 assay and live/dead staining assay in accordance with the instructions. Afterwards, the cytoskeleton and spreading morphology of the MC3T3-E1 cells on the scaffolds were observed by CLSM and SEM, respectively. After being cultured for 3 days, the MC3T3-E1 cells on the scaffolds were stained with phalloidin and vinculin to determine the F-actin and focal adhesion (FA) distributions, respectively. The fluorescence images were observed using CLSM.

For the evaluation of osteogenic differentiation, an ALP activity assay was conducted as previously described 22. After being cultured in osteogenic induction medium for 7 days, the cell/scaffold complex was stained with a BCIP/NBT ALP color development kit, and the intracellular ALP activity was measured by using a commercial ALP assay kit and a BCA protein assay kit following the manufacturer's instructions. On day 14 after osteogenic induction, ARS staining was performed to detect mineralized matrix formation according to the manufacturer's instructions. To quantify the mineralization results, 10% cetylpyridinium chloride was used to dissolve the deposited calcium, and the absorbance of the sample solution was measured at a wavelength of 562 nm using a UV-vis spectrophotometer.

Furthermore, quantitative real-time polymerase chain reaction (qRT-PCR) was used to detect the relative expression levels of osteogenesis-related genes on day 7. Total RNA from cells in different groups was collected using TRIzol reagent following the manufacturer's instructions. The data were normalized to the housekeeping gene GAPDH, and the mRNA levels were calculated using the 2^-ΔΔCT^ method. The primer sequences are presented in **Table S1**. For immunofluorescence staining, the cells were washed and fixed in 4% paraformaldehyde for 30 min, permeabilized with 0.1% Triton and blocked with 1% bovine serum albumin for 30 min. Then, the samples were incubated with primary antibody at 4 °C overnight before being treated with the fluorescently labeled secondary antibody. The nuclei were then counterstained with DAPI and visualized by CLSM. The relative fluorescence intensity was determined via ImageJ software.

### *In vitro* evaluation of immunomodulatory performance

The intracellular ROS scavenging abilities of the prepared scaffold system were measured by an ROS assay kit. Briefly, to mimic oxidative stress and macrophage activation in the diabetic microenvironment, RAW264.7 cells were first treated with LPS (100 ng/mL) for 2 days and cocultured on various scaffolds with or without NIR irradiation for 3 days as described above. This irradiation procedure was repeated every two days with four cycles of laser on and off processes. The peak irradiation temperature was maintained at 42 ± 1 °C. Subsequently, the cell/scaffold samples were incubated with DCFH-DA (25 μM), followed by incubation for 20 min in the dark. Finally, the immunofluorescence-labeled cells were visualized by CLSM, and the intracellular ROS levels were quantified by flow cytometry.

After 3 days of coculture, the cell morphologies after different treatments were observed by cytoskeleton staining as described above. Subsequently, the expression of M1 and M2 markers was detected by flow cytometry, immunofluorescence staining and qRT-PCR assays. The macrophages were treated with C86 antibody (Biolegend, 105011) and CD206 antibody (Biolegend, 141703) and then analyzed by flow cytometry according to the manufacturer's protocol. For immunofluorescence staining, the cells were stained for iNOS and CD206 at 4 °C. Then, the stained samples were observed by CLSM. The expression levels of inflammation-related genes were quantified by qRT-PCR as described above. The results were normalized against GAPDH expression and analyzed using the 2^-ΔΔCt^ method. The forward and reverse primer sequences are listed in **Table S1**. This experimental design allowed for the assessment of the potential immunomodulatory effects of PGCZ scaffolds on RAW264.7 cells stimulated with LPS.

### *In vitro* evaluation of angiogenic activity

To explore the effects of macrophage-endothelial cell crosstalk on vascularization *in vitro*, a coculture model was established using a Transwell chamber system, as previously described 90. Briefly, RAW264.7 cells were seeded onto the surface of the scaffolds, followed by incubation at 37 °C for 30 min. The complex was then transferred to the upper chamber of the Transwell, while HUVECs at a density of 2 × 10^4^ cells per well were seeded into the lower chamber of a 24-well Transwell plate with or without NIR irradiation (1 W/cm^2^) as mentioned above. Furthermore, the concentrations of immune-related cytokines released into the supernatant were measured by commercial ELISA kits (Meimian, Jiangsu, China) according to the manufacturer's instructions. For the wound healing assay, a linear scratch was created with a 10 μL pipette tip on the HUVEC monolayer when the cells reached 70-80% confluency, and the cells were rinsed with PBS to remove cellular debris. After 24 h, the cells were fixed with 4% paraformaldehyde, stained with 0.1% crystal violet solution, and then observed under an optical microscope. The scratch area was measured using ImageJ software. For the Transwell migration assay, the prepared cell/scaffold complex was placed in the lower chamber of the Transwell system, while HUVECs were seeded into the upper chamber of 24-well Transwell plates. After 24 h, the migrated cells were observed with an optical microscope and quantified using ImageJ software. For the *in vitro* tube formation assay, the prepared cell/scaffold complex was placed in the upper chamber of a 24-well Transwell plate, and HUVECs were seeded on the surface of growth factor-reduced Matrigel in the lower chamber and then incubated at 37 °C. After 6 h, the cells were stained with TRITC-labeled phalloidin/DAPI and observed by CLSM. The average parameters of tube formation, including the vessel percentage area and the total number of junctions, were quantified using AngioTool (National Cancer Institute, NIH).

After 7 days of coculture, qRT-PCR was conducted to evaluate the expression of angiogenesis-related genes in HUVECs, as described above. GAPDH was used as an internal reference, and relative gene expression levels were calculated by the 2^-ΔΔCT^ method. The primer sequences are shown in **Table S2**. For the immunofluorescence staining assay, the samples were fixed, permeabilized, and incubated with primary antibody following the same method described above. The fluorescence images were detected using CLSM.

### Antibacterial activity assessment

In this study, both *S. aureus* (Gram-positive bacteria) and *E. coli* (Gram-negative bacteria) were used as bacterial models to evaluate the antibacterial properties of the scaffolds. First, the bacterial growth activity was evaluated by a turbidimetric method 42. Briefly, bacterial suspensions (~10^6^ CFU/mL) were cocultured with various scaffolds, followed by exposure to NIR laser irradiation (808 nm, 1 W/cm^2^) for four “on-off” heating cycles, as mentioned above. The peak irradiation temperature was maintained at 42 ± 1 °C. After 24 h of culture, the absorbance of the bacterial suspension at 600 nm was measured using a UV-vis spectrophotometer. Moreover, changes in the turbidity of the bacterial suspension were imaged by a digital camera. A bacterial suspension without any treatment was used as a control sample.

Moreover, bacterial plate colony counting experiments and live/dead bacterial staining assays were used to evaluate the antibacterial performance of the scaffold samples, as previously described 28. Following a procedure similar to that described above, the treated bacterial suspension with an appropriate dilution ratio was spread onto Luria-Bertani (LB) plates (1.5% agar) at 37 °C for another 24 h. Then, the bacterial colonies on the plates were photographed and counted. Meanwhile, the treated bacterial suspension was subjected to a live/dead bacterial staining assay following the manufacturer's instructions, and fluorescence images were captured by CLSM. Finally, the above treated bacterial suspensions were collected, centrifuged, fixed with 2.5% glutaraldehyde for 4 h at 4 °C, dehydrated in gradients (30%, 50%, 70%, 90%, and 100% ethanol) and dried via critical point drying. After coating with gold, the morphology of the treated bacteria was observed by SEM.

The effect on bacterial biofilm formation was determined by crystal violet staining, as previously described 91. Briefly, bacterial suspensions were added to a 24-well plate and incubated for 2 days at 37 °C to form mature biofilms. Subsequently, various scaffolds were cocultured with bacterial biofilms at 37 °C for 2 h. Similarly, the bacteria in the PGCZ+NIR group were exposed to NIR laser irradiation (808 nm, 1 W/cm^2^) for 75 s and maintained for four on/off cycles, as mentioned above. Then, the plates were gently washed with PBS three times, followed by staining with 0.1% crystal violet solution for 20 min. After rinsing with PBS, the stained biofilms were imaged by a digital camera. To quantify the remaining biofilm biomass, the stained samples were dissolved in ethanol and measured by a UV-vis spectrophotometer at an absorbance of 590 nm. To observe the 3D structure of the bacterial biofilm, the biofilm was established on confocal dishes as previously described 92. After various treatments, the formed biofilm was stained using a live/dead BacLight bacterial viability kit (Invitrogen, USA) following the manufacturer's instructions. Afterwards, the samples were gently rinsed with PBS, and the stained biofilms were directly observed via CLSM.

### Rat subcutaneous implantation experiment

The hydrogels (5 mm in diameter and 1 mm in thickness) were implanted into the subcutaneous tissue of rats to assess the early immune response and neovascularization. Briefly, twenty-four SD rats (6 weeks old, 190-220 g) were randomly divided into four groups (6 rats per group), namely, the (1) PCL, (2) PGC, (3) PGCZ, and (4) PGCZ+NIR groups, and anesthetized by inhalation of isoflurane (2%). Then, a sagittal subcutaneous incision (~3 cm in length) was made on the back of each rat, and the sterilized scaffold samples were implanted. All surgical procedures were conducted by the same surgeons in a sterile environment. For *in vivo* evaluation of the photothermal properties, the PGCZ+NIR group was subjected to periodic NIR irradiation (1 W/cm^2^, 808 nm) for 75 s and cooled for 2 min, and this procedure was repeated 4 times each time without interruption. The intermittent and continuous NIR irradiation procedures mentioned above were performed every 2 days, and the peak irradiation temperature was maintained at 42 ± 1 °C. During the photothermal procedure, an infrared thermal imaging instrument was used to record the temperature changes. After 7 and 14 days of implantation, the implanted samples were collected and immersed in 4% paraformaldehyde for further processing. After paraffin embedding the tissue samples, macrophage polarization was evaluated by immunohistochemical (iNOS and CD206) and immunofluorescence (CD86 and CD206) staining. Additionally, angiogenesis was evaluated by immunohistochemical staining for CD31 and α-SMA. The stained samples were scanned by an automatic digital slide scanner and quantified by ImageJ software. Furthermore, the secretion of inflammatory and pro-healing cytokines from the collected scaffold implants, as well as the levels of CD4 and CD8 in the serum of the rats, were examined using ELISA kits following the manufacturer's instructions.

### Diabetic rat calvarial bone defect model

A diabetic rat calvarial bone defect model was induced in SD rats (6 weeks old, 190-220 g). Briefly, male SD rats in a specific pathogen-free animal room received intraperitoneal injections of STZ (60 mg/kg), which was dissolved in sodium citrate buffer (pH = 4.5). After 2 weeks of adaptive feeding, rats whose blood glucose was >16.7 mmol/L were selected, which means that the STZ-induced diabetic rat model was successfully constructed 93. Following the aforementioned process, two critical-sized cranial defects 5 mm in diameter were created on both sides of the skull after anesthetization. The incision was carefully sutured after scaffold implantation in the defects and disinfected with iodophor. The diabetic rats were randomly divided into five groups (12 rats per group): (1) the control group (bone defects without implantation), (2) the PCL group (implanted with PCL scaffold), (3) the PGC group (implanted with PGC scaffold), (4) the PGCZ group (implanted with PGCZ scaffold), and (5) the PGCZ+NIR group (implanted with PGCZ scaffold and then subjected to intermittent and continuous NIR irradiation every 2 days as mentioned above). Four cycles of laser on and off processes were carried out at each time point. The rats were sacrificed at 2, 4, and 8 weeks after implantation, and cranial samples were harvested and fixed with 4% paraformaldehyde for subsequent experiments. After micro-CT scanning and reconstruction analysis, immunofluorescence, immunohistochemistry, H&E, MST, Goldner's trichrome (GST), and tartrate-resistant acid phosphatase (TRAP) staining were performed on the sample sections according to the manufacturers' protocols. Specifically, the tissue sections were subjected to immunofluorescence (CD86 and CD206) and immunohistochemical (TNF-α, IL-10, BMP-2, and VEGF) analyses to further assess early immune microenvironment reprogramming at 2 weeks. Additionally, some of the harvested samples were processed for flow cytometry and qRT-PCR analysis according to the manufacturer's instructions as described above. The primer sequences are shown in **Table S1**. At 4 weeks, immunofluorescence staining for CD31, α-SMA, CD44, and CD90 was performed to investigate the local neovascularization and *in situ* recruitment of MSCs during bone repair. At 8 weeks, H&E, MST, and Goldner's trichrome staining were performed to evaluate the bone repair efficiency under diabetic conditions. Simultaneously, TRAP staining and immunohistochemical (Col-1, Runx2, OPN, and OCN) analysis were conducted to evaluate bone remodeling and mineralization. Finally, major organs, including the heart, liver, lung, spleen, and kidney, were collected from the rats for H&E staining at 8 weeks.

### Statistical analysis

Statistical analysis in this research was performed using Origin 2018 software (Origin Lab Corporation, USA) by one-way ANOVA with Tukey's test. The results are expressed as the mean ± standard deviation (mean ± SD) of three representative experiments. Data with abnormal distribution or heterogeneity of variance were analyzed by the Mann-Whitney U test and Kruskal-Wallis's nonparametric test. The values were considered significant at p* or p^#^, represented by a p value < 0.05, and highly significant at p** or p^##^, with a p value < 0.01.

## Supplementary Material

Supplementary figures and table.
