# Supplementary material for: Bioinspired soft-hard combined system with mild photothermal therapeutic activity promotes diabetic bone defect healing via synergetic effects of immune activation and angiogenesis
Source: Theranostics. 2024 Jul 1;14(10):4014–57. doi: 10.7150/thno.97335 (PMC11234279; doi:10.7150/thno.97335)
Supplement: Supplementary file 1 — Supplementary figures and table. [file thnov14p4014s1.pdf]

Supporting Information

Bioinspired soft-hard combined system with mild photothermal therapeutic activity promotes diabetic bone defect healing via synergetic effects of immune activation and angiogenesis

Minhao Wu[1#], Huifan Liu[2#], Yufan Zhu[1#], Ping Wu[3], Yun Chen[4], Zhouming Deng[1], Xiaobin Zhu[1*], Lin Cai[1*]

[1] Department of Spine Surgery and Musculoskeletal Tumor, Zhongnan Hospital of Wuhan University, 168 Donghu Street, Wuchang District, Wuhan 430071 Hubei, People's Republic of China

[2] Department of Anesthesiology, Research Centre of Anesthesiology and Critical Care Medicine, Zhongnan Hospital of Wuhan University, Wuhan, Hubei, China

[3] National Key laboratory of macromolecular drug development and manufacturing, School of Pharmaceutical Science, Wenzhou Medical University, Wenzhou, 325035, China

[4] Department of Biomedical Engineering and Hubei Province Key Laboratory of Allergy and Immune Related Disease, TaiKang Medical School (School of Basic Medicine Sciences), Wuhan University, Wuhan 430071, China

[#] These authors contributed equally to this work
* Correspondence should be addressed to:

Dr. Lin Cai. Email: orthopedics@whu.edu.cn
Department of Spine Surgery and Musculoskeletal Tumor, Zhongnan Hospital of Wuhan University, 168 Donghu Street, Wuchang District, Wuhan 430071 Hubei, People's Republic of China

Dr. Xiaobin Zhu. Email: xiaobinzhu@whu.edu.cn
Department of Spine Surgery and Musculoskeletal Tumor, Zhongnan Hospital of Wuhan University, 168 Donghu Street, Wuchang District, Wuhan 430071 Hubei, People's Republic of China

Supplementary figures and tables

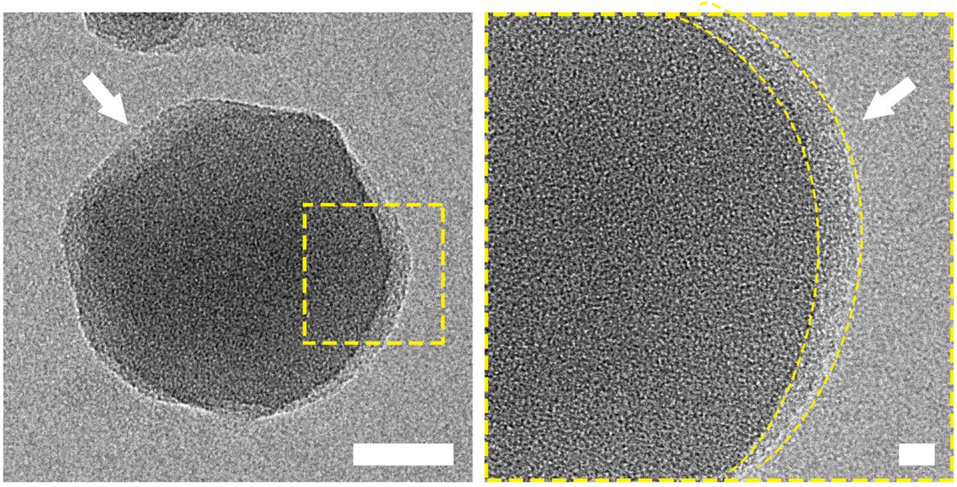

Figure S1. TEM images of ZIF-8@PDA nanoparticles at different magnifications. The white arrows indicate the PDA layer. Scale bar: 50 and 10 nm for the left and right images, respectively.

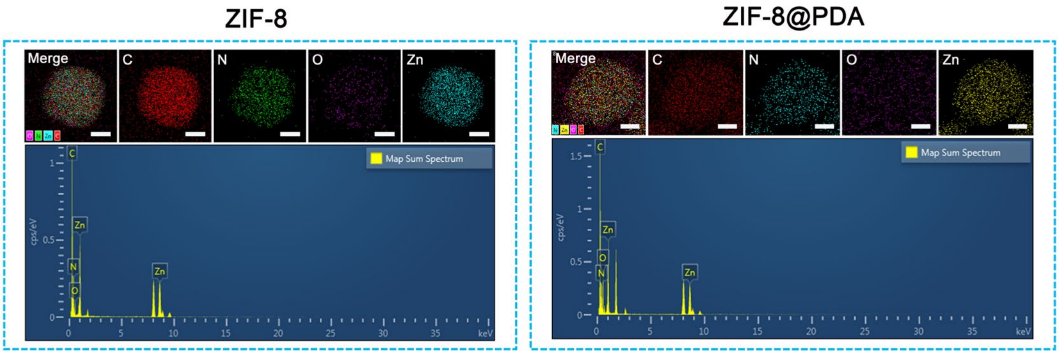

Figure S2. TEM-EDS elemental mapping images and spectra of the ZIF-8 and ZIF-8@PDA nanoparticles. Scale bar: 50 nm.

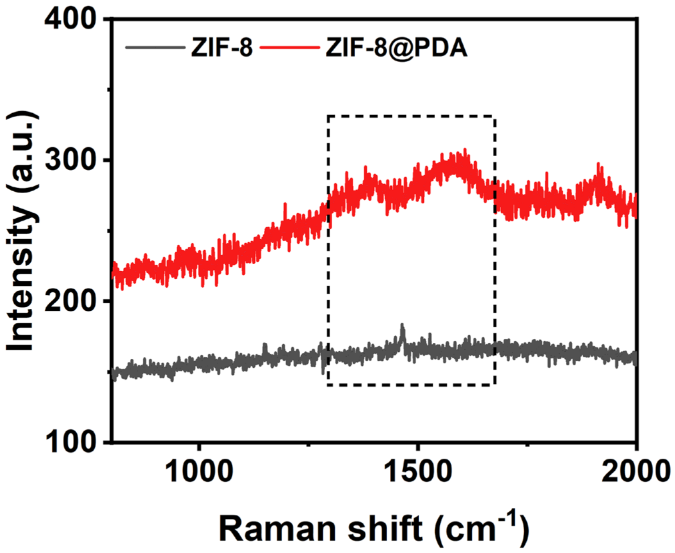

Figure S3. Raman spectra of the ZIF-8 and ZIF-8@PDA nanoparticles.

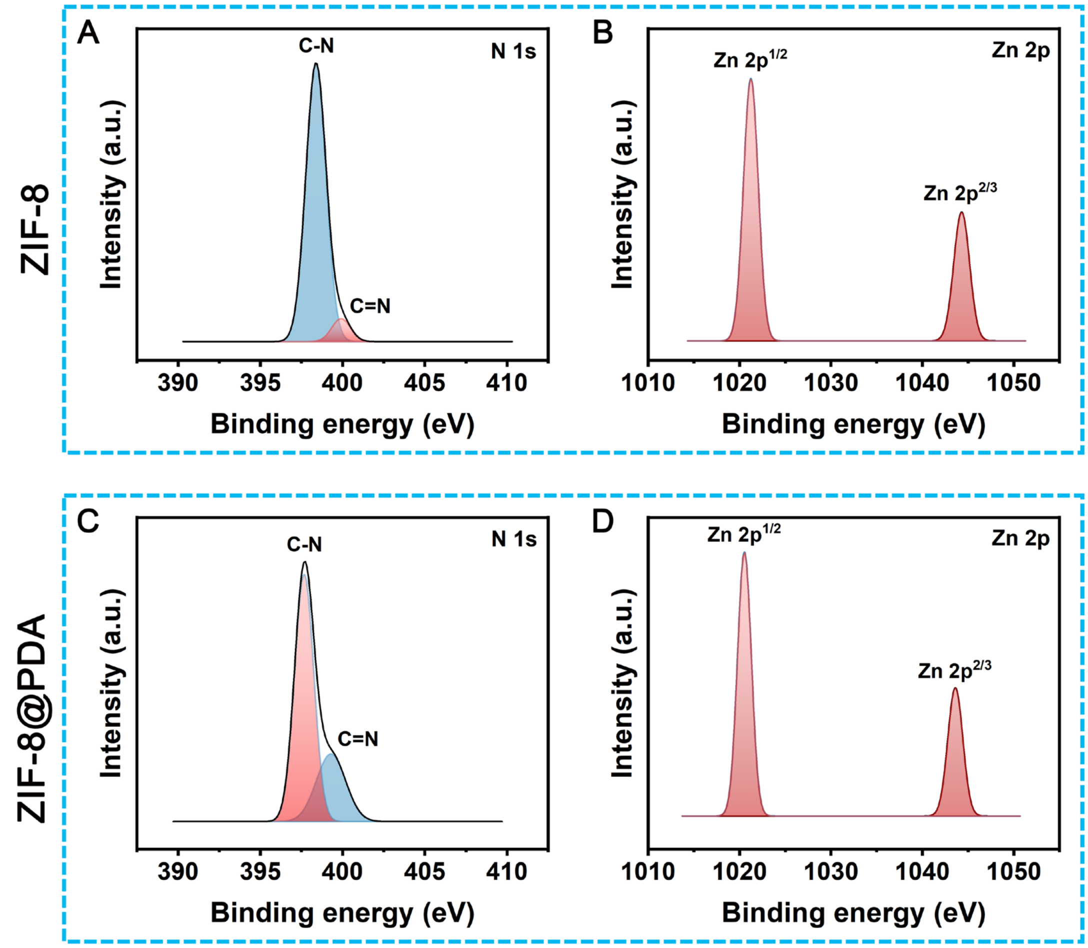

Figure S4. High-resolution XPS spectra of N 1s **(A, C)** and Zn 2p **(B, D)**.

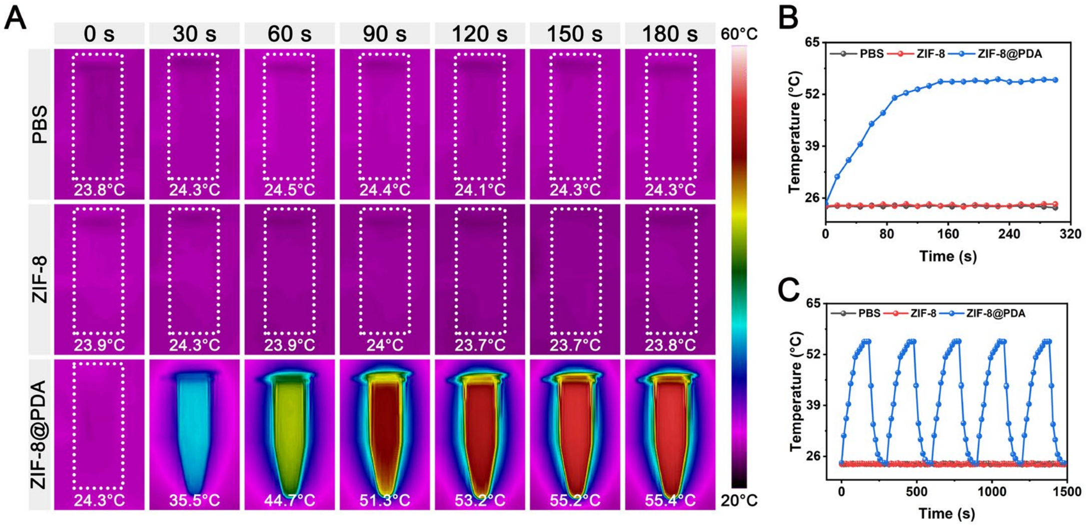

Figure S5. **(A)** Infrared thermal images, **(B)** temperature curves, and **(C)** thermal cycle profiles of the prepared nanoparticle aqueous solutions (1 mg/mL) under NIR laser

radiation (808 nm, 1 W/cm^2).

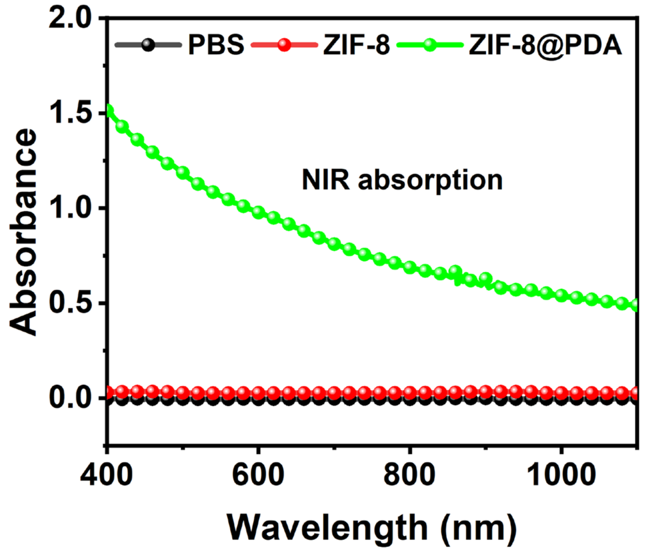

Figure S6. UV-vis-NIR absorption spectra.

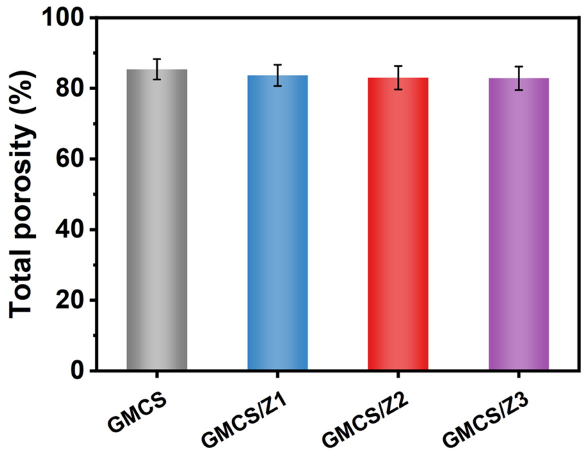

Figure S7. Total porosity of different hydrogels. Data are presented as the mean ± SD (n = 3).

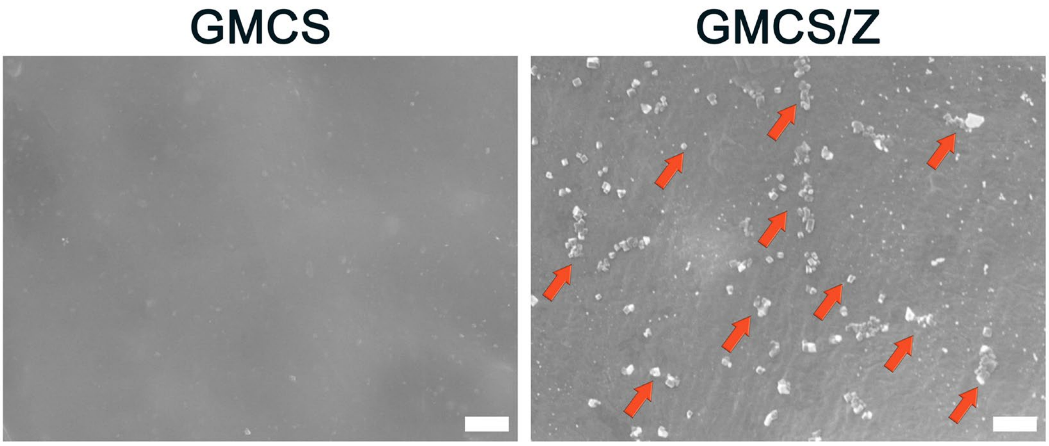

Figure S8. The distribution of ZIF-8@PDA nanoparticles in the hydrogel was observed by SEM. The red arrows represent the nanoscale particles on the hydrogel surface. Scale bar: 1 μm.

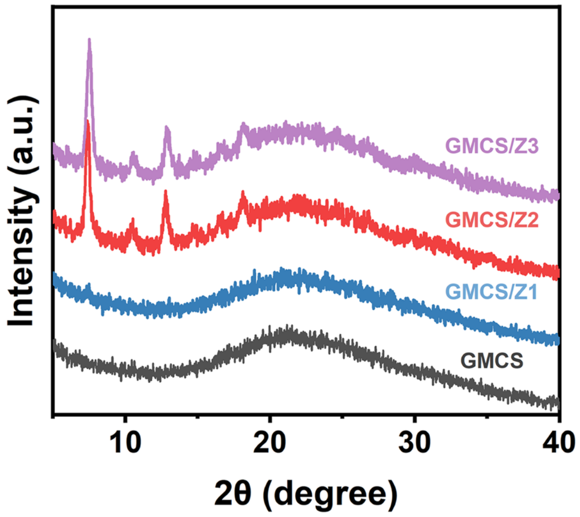

Figure S9. XRD spectra of different hydrogels.

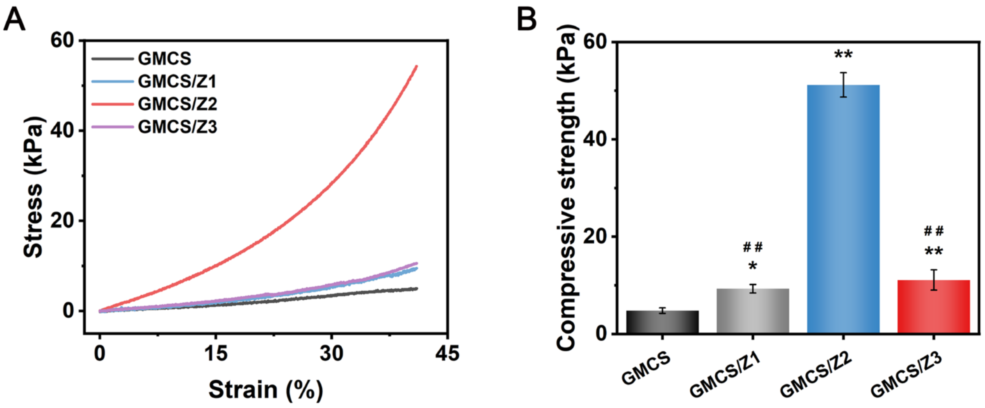

Figure S10. (A) Compressive stress-strain curves and **(B)** compressive strength of

different hydrogels. Data are presented as the mean ± SD (n = 3). *P < 0.05 and **P < 0.01 indicate significant differences compared with the GMCS group. #P < 0.05 and # #P < 0.01 indicate significant differences compared with the GMCS/Z2 group.

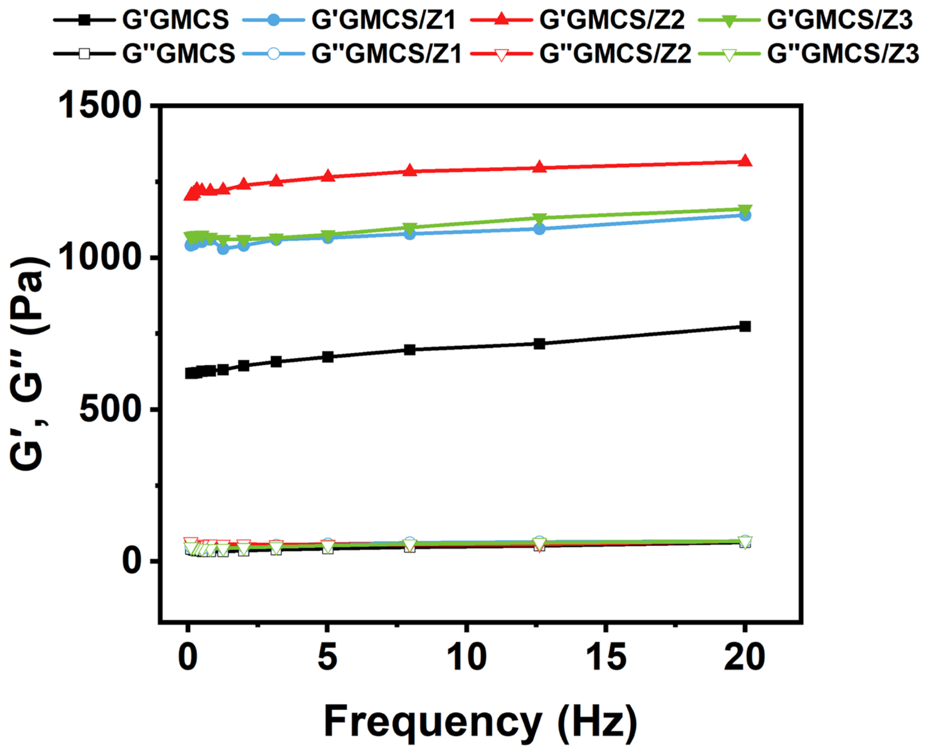

Figure S11. Rheological properties of different hydrogels.

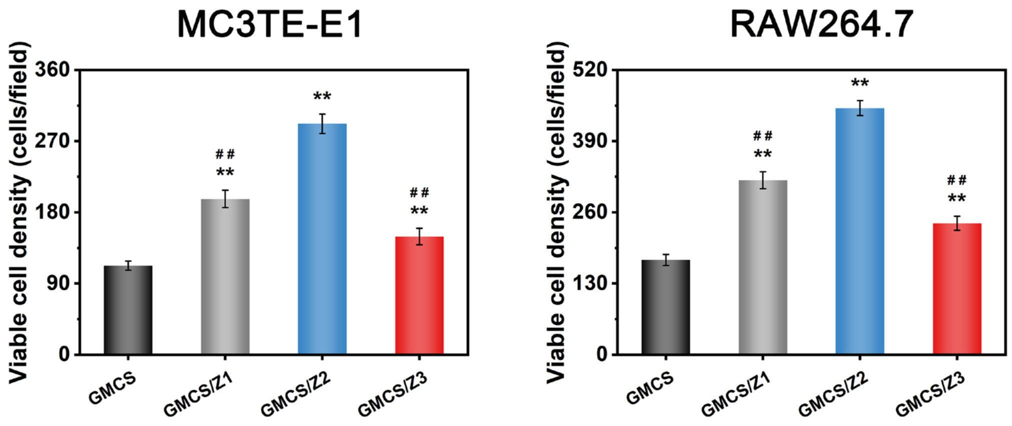

Figure S12. Quantitative analysis of cell density based on live/dead staining assay. Data are presented as the mean ± SD (n = 3). *P < 0.05 and **P < 0.01 indicate significant differences compared with the GMCS group. #P < 0.05 and # #P < 0.01 indicate significant differences compared with the GMCS/Z2 group.

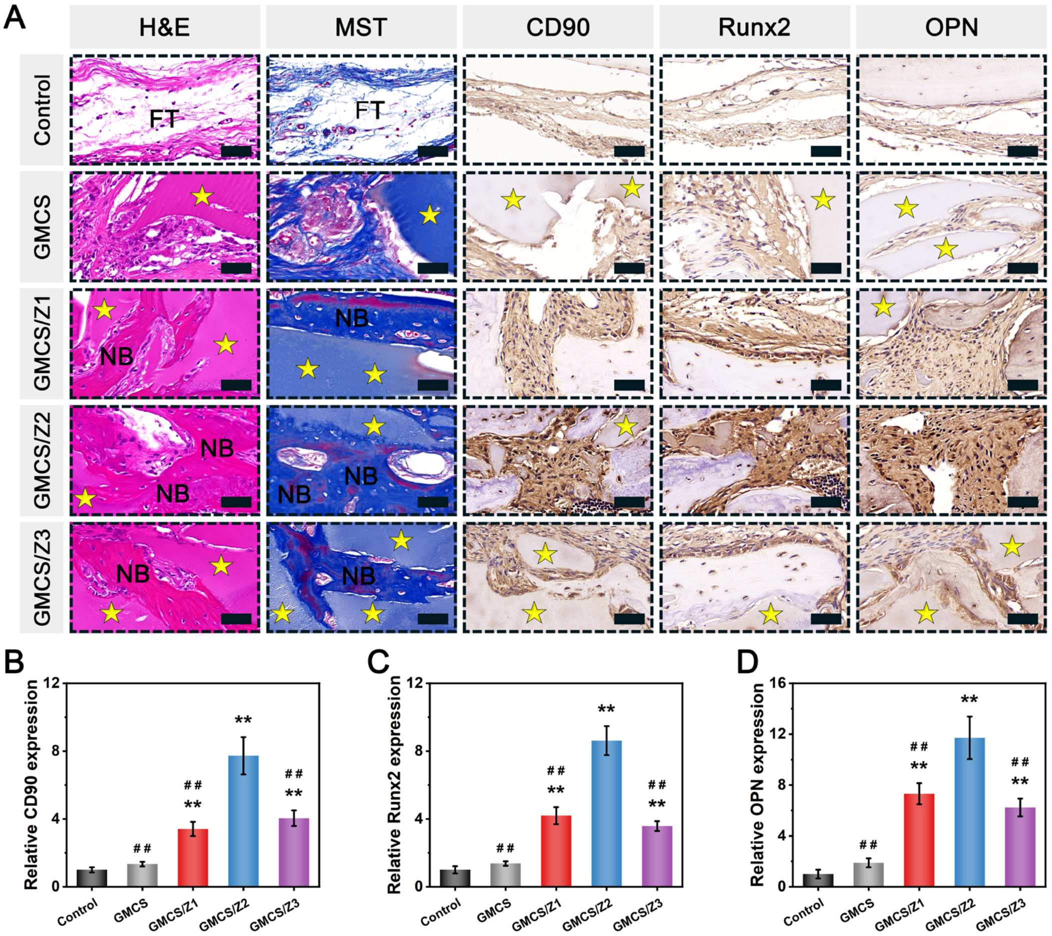

Figure S13. (A) H&E staining, MST staining, and immunohistochemical staining images of decalcified bone tissue. FT: fibrous tissue. NB: newly formed bone tissue. The yellow asterisks represent the residual materials. Scale bar: 50 μm. Quantitative expression of **(B)** CD90, **(C)** Runx2, and **(D)** OPN. Data are presented as the mean ± SD (n = 3). Data are presented as the mean ± SD (n = 3). *P < 0.05 and **P < 0.01 indicate significant differences compared with the control group. $^{\#}$P < 0.05 and $^{\#\#}$P < 0.01 indicate significant differences compared with the GMCS/Z2 group.

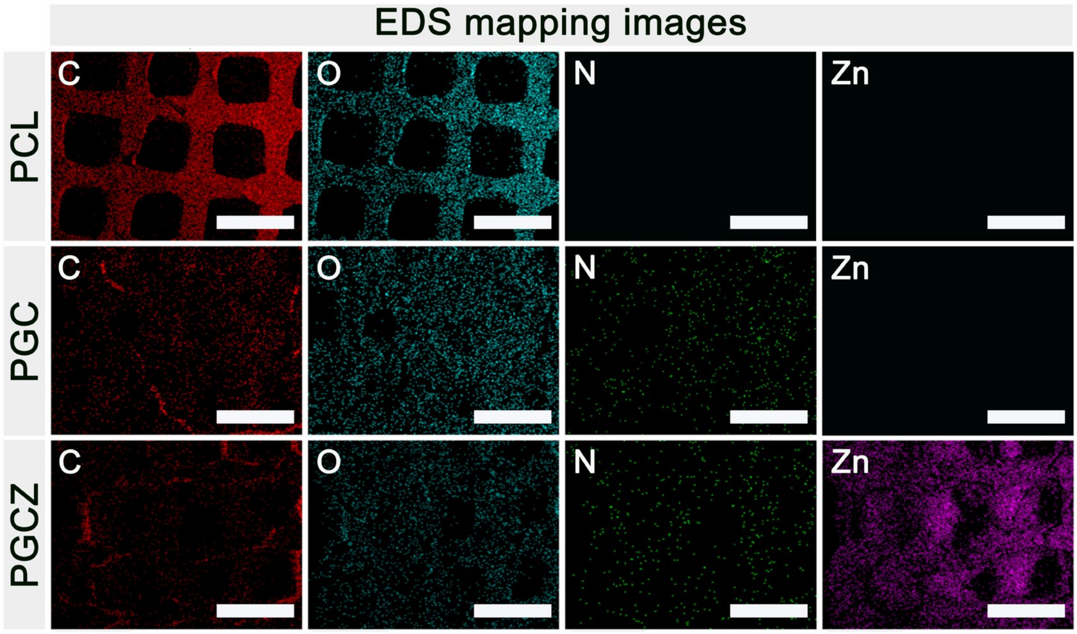

Figure S14. SEM-EDS elemental mapping images of different scaffolds. Scale bar: 1 μm.

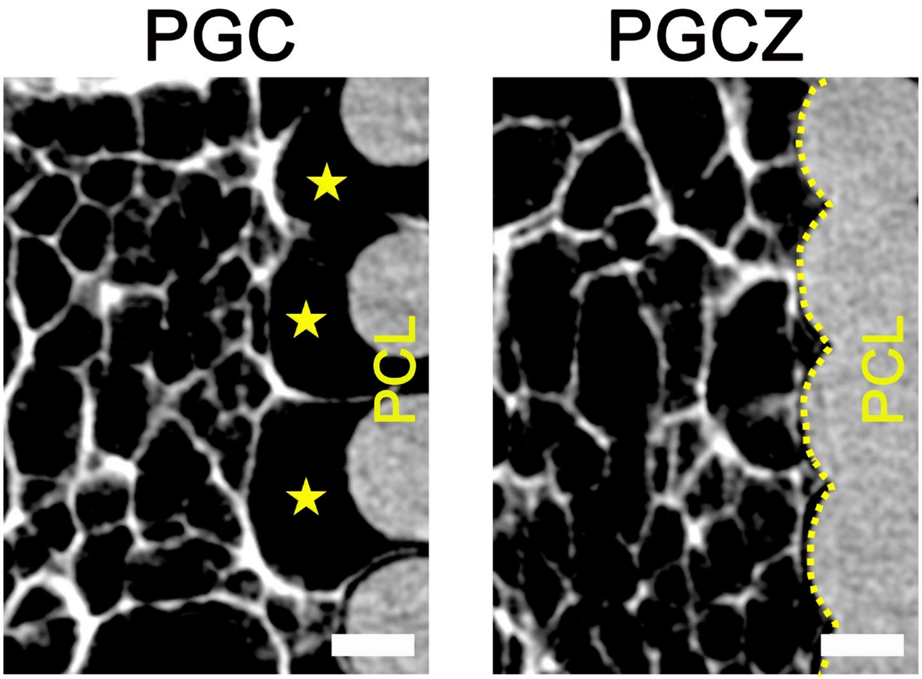

Figure S15. Micro-CT images of cross-sectional scaffolds. The yellow asterisks represent the separation gap between the hydrogel and the PCL. The yellow dotted lines indicate the interfacial contact of the hydrogel with the PCL. Scale bar: 200 μm.

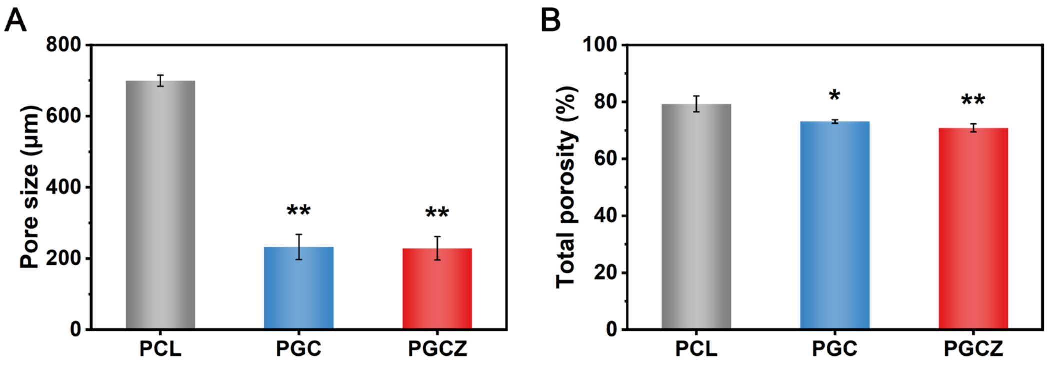

Figure S16. The average (**A**) pore size and (**B**) porosity of the different scaffolds. Data are presented as the mean ± SD (n = 3). *P < 0.05 and **P < 0.01 indicate significant differences compared with the PCL group.

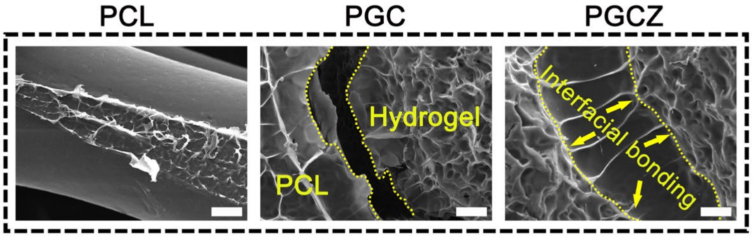

Figure S17. SEM images of the various scaffolds after degradation. Scale bar: 50 μm.

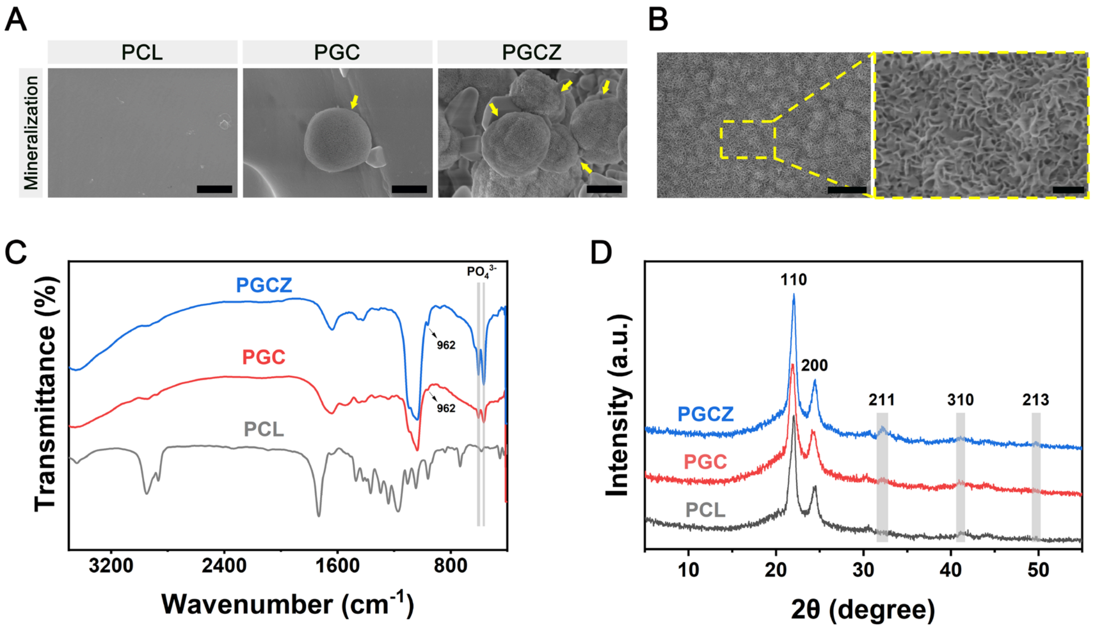

Figure S18. (A) SEM images of different scaffolds after mineralization. The yellow arrows indicate in situ mineralized hydroxyapatite nanocrystals. Scale bar: 2 μm. **(B)** SEM images of PGCZ scaffolds at different magnifications after mineralization. Scale bar: 2 μm and 400 nm for the left and right images, respectively. **(C)** FTIR spectra and **(D)** XRD patterns of different scaffolds after mineralization.

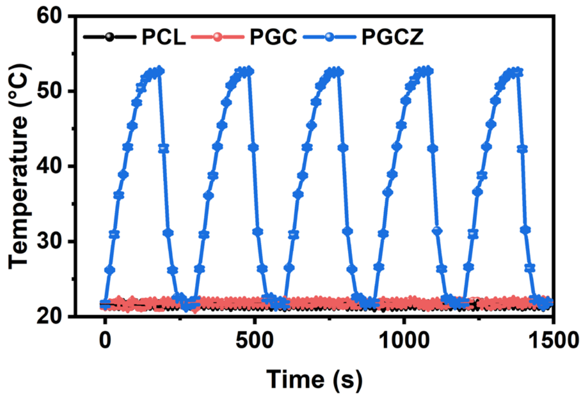

Figure S19. Thermal cycle profiles of the various scaffolds under NIR laser radiation (808 nm, 1 W/cm^2). Data are presented as the mean ± SD (n = 3).

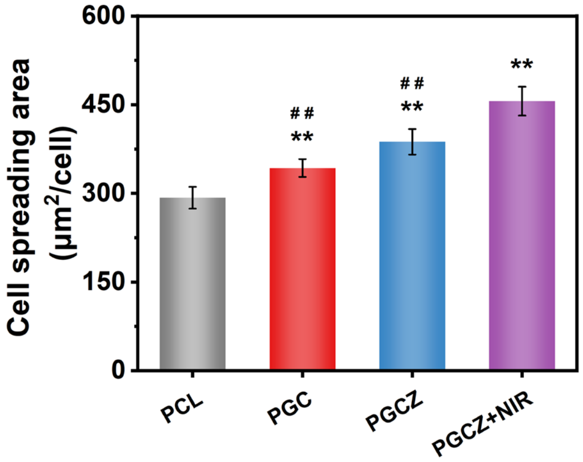

Figure S20. Quantitative analysis of cell spreading of MC3T3-E1 cells. Data are presented as the mean ± SD (n = 3). *P < 0.05 and **P < 0.01 indicate significant differences compared with the PCL group. #P < 0.05 and # #P < 0.01 indicate significant differences compared with the PGCZ+NIR group.

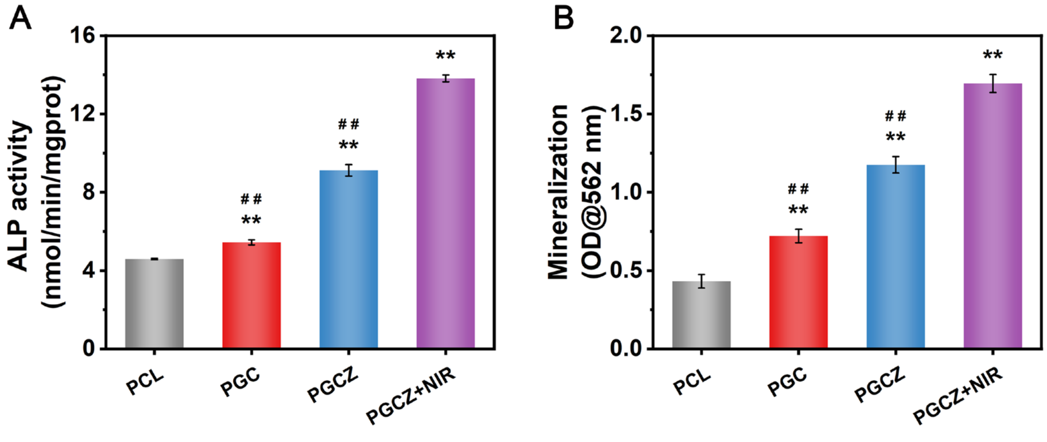

Figure S21. Quantitative analysis of **(A)** ALP activity and **(B)** ECM mineralization in MC3T3-E1 cells. Data are presented as the mean ± SD (n = 3). *P < 0.05 and **P < 0.01 indicate significant differences compared with the PCL group. $^{\#}$P < 0.05 and $^{\#\#}$P < 0.01 indicate significant differences compared with the PGCZ+NIR group.

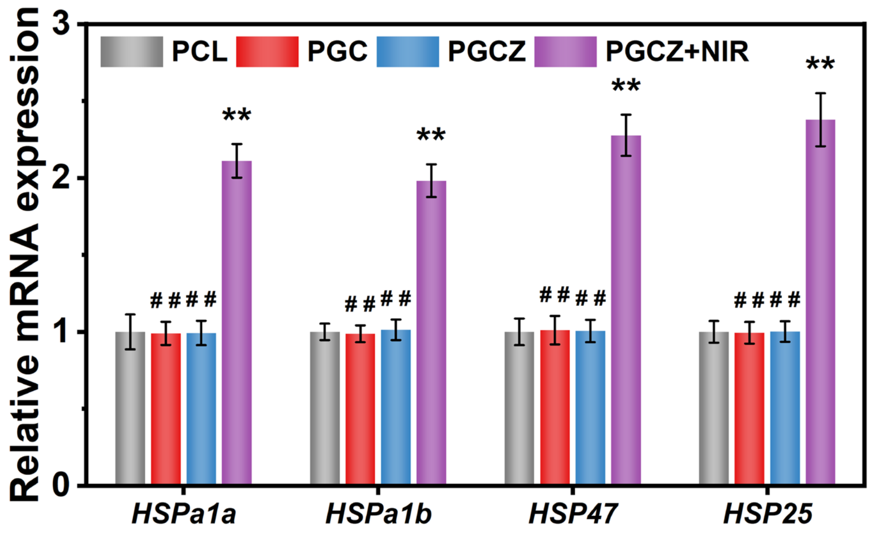

Figure S22. Relative mRNA expression of *HSPa1a*, *HSPa1b*, *HSP47*, and *HSP25* in MC3T3-E1 cells cultured on different scaffolds with or without NIR treatment. Data are presented as the mean ± SD (n = 3). *P < 0.05 and **P < 0.01 indicate significant differences compared with the PCL group. $^{\#}$P < 0.05 and $^{\#\#}$P < 0.01 indicate significant differences compared with the PGCZ+NIR group.

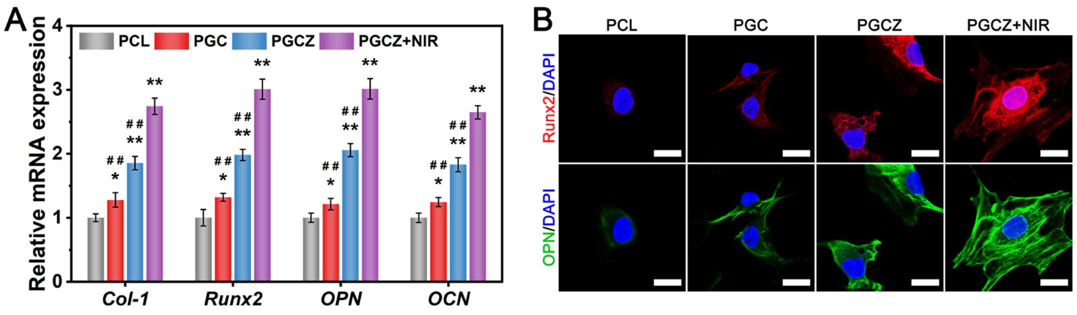

Figure S23. (A) Relative mRNA expression of osteogenesis-related genes, including *Col-1*, *Runx2*, *OPN*, and *OCN*, in MC3T3-E1 cells after 7 days of co-culture. **(B)** Immunofluorescence staining images of Runx2 and OPN (red: Runx2; green: OPN; blue: DAPI). Scale bar: 20 μm. Data are presented as the mean ± SD (n = 3). *P < 0.05 and **P < 0.01 indicate significant differences compared with the PCL group. $^{\#}$P < 0.05 and $^{\#\#}$P < 0.01 indicate significant differences compared with the PGCZ+NIR group.

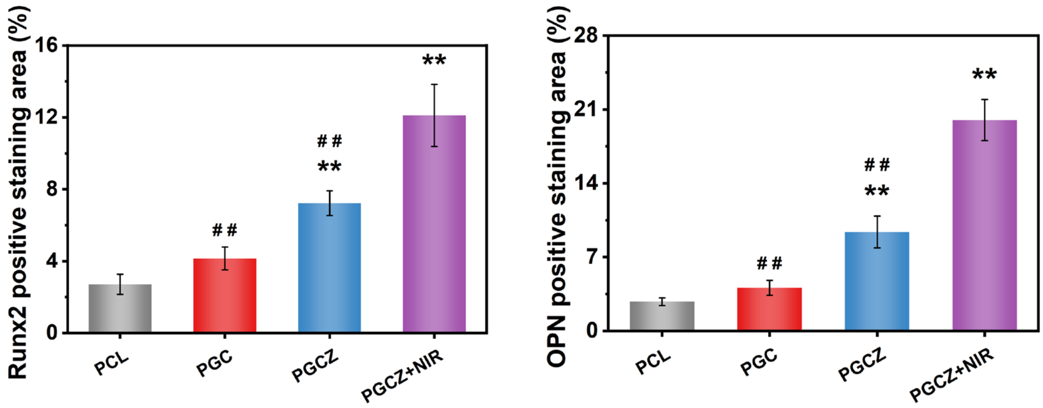

Figure S24. Quantitative analysis of positive staining areas. Data are presented as the mean ± SD (n = 3). *P < 0.05 and **P < 0.01 indicate significant differences compared with the PCL group. #P < 0.05 and ##P < 0.01 indicate significant differences compared with the PGCZ+NIR group.

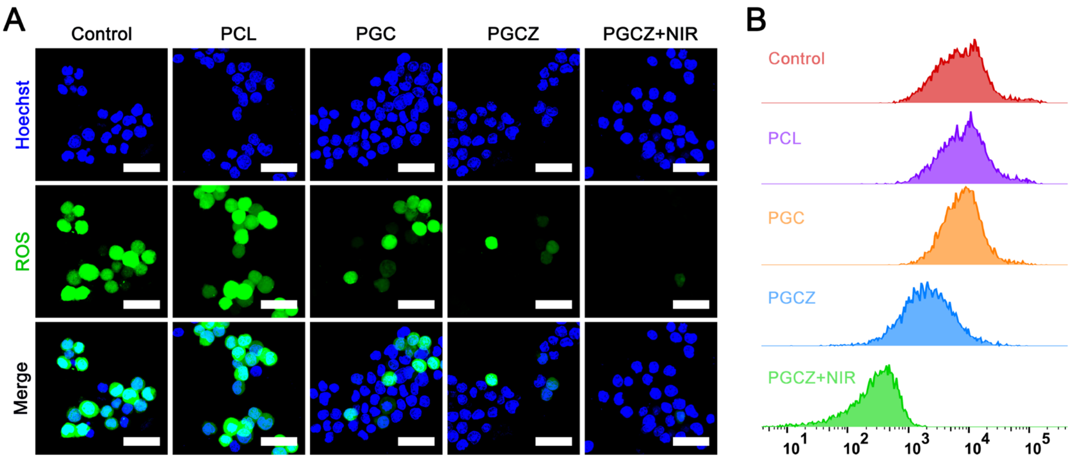

Figure S25. **(A)** Confocal fluorescence images and **(B)** flow cytometry analysis of intracellular ROS in RAW264.7 cells after different treatments (green: ROS; blue: Hoechst). Scale bar: 25 μm.

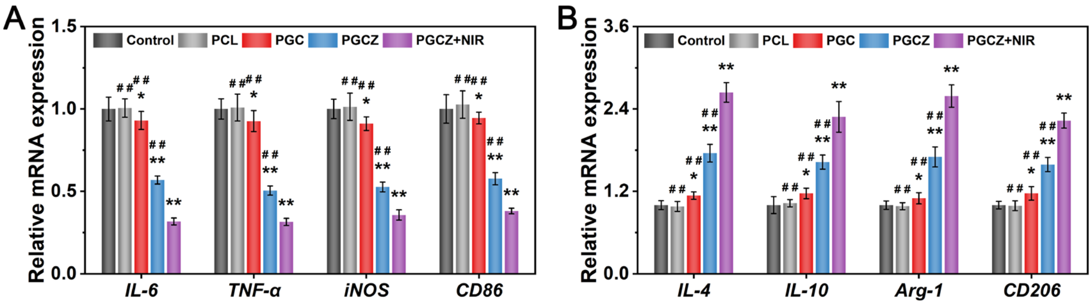

Figure S26. Relative mRNA expression of **(A)** proinflammatory and **(B)** anti-inflammatory markers in macrophages after 3 days of co-culture. Data are presented as the mean ± SD (n = 3). *P < 0.05 and **P < 0.01 indicate significant differences compared with the control

group. $^{\#}P$ < 0.05 and $^{\#\#}P$ < 0.01 indicate significant differences compared with the PGCZ+NIR group.

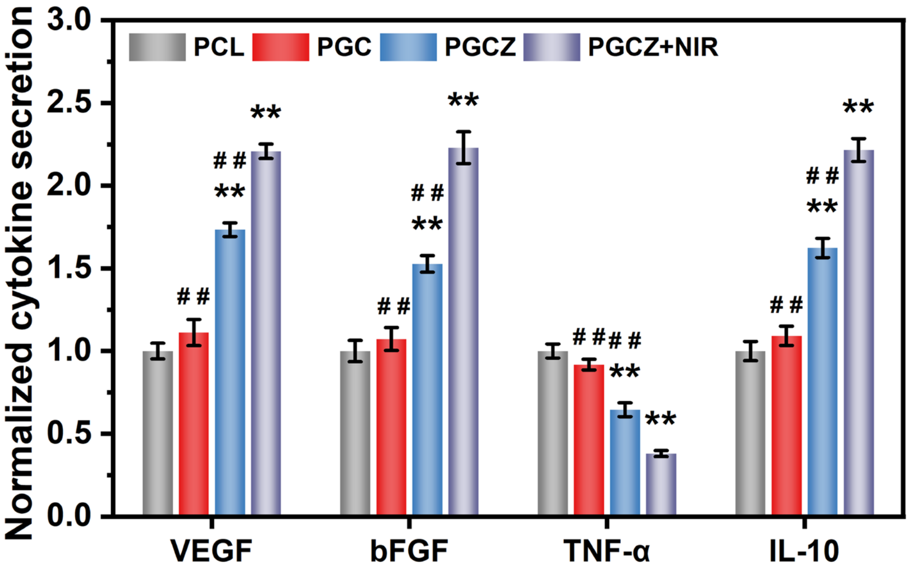

Figure S27. Secretion of angiogenic (VEGF and bFGF) and inflammatory (TNF-α and IL-10) cytokines by macrophages in the different groups. Data are presented as the mean ± SD (n = 3). *P < 0.05 and **P < 0.01 indicate significant differences compared with the PCL group. $^{\#}P$ < 0.05 and $^{\#\#}P$ < 0.01 indicate significant differences compared with the PGCZ+NIR group.

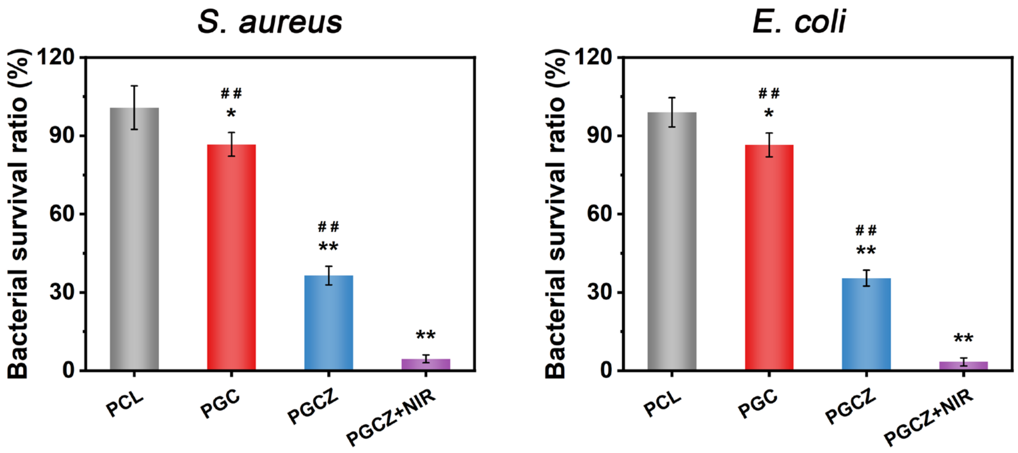

Figure S28. Statistical analysis of the survival ratio of *S. aureus* and *E. coli* based on the spread plate method. Data are presented as the mean ± SD (n = 3). *P < 0.05 and **P < 0.01 indicate significant differences compared with the PCL group. $^{\#}P$ < 0.05 and $^{\#\#}P$ < 0.01 indicate significant differences compared with the PGCZ+NIR group.

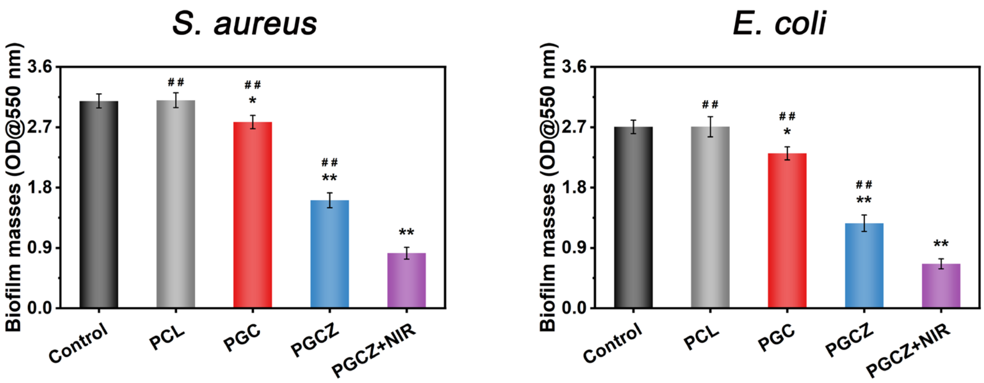

Figure S29. Statistical analysis of *S. aureus* and *E. coli* bacterial biofilms. Data are presented as the mean ± SD (n = 3). *P < 0.05 and **P < 0.01 indicate significant differences compared with the control group. #P < 0.05 and # #P < 0.01 indicate significant differences compared with the PGCZ+NIR group.

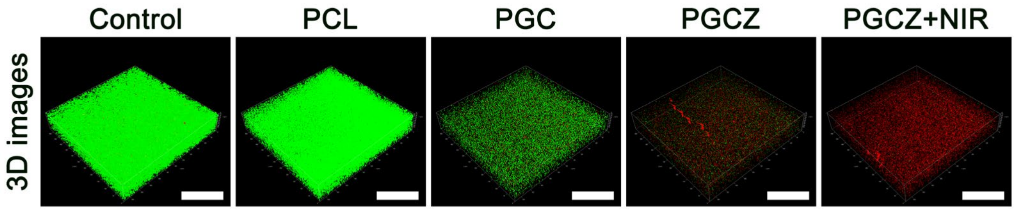

Figure S30. 3D-reconstructed CLSM images of *S. aureus* bacterial biofilms in various groups after treatment. Scale bar: 200 μm.

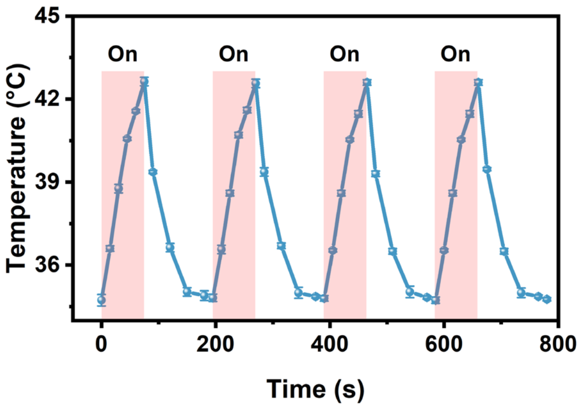

Figure S31. Photothermal heating curves of the implantation site under NIR irradiation (1 W/cm^2, 808 nm) with four on/off cycles. Data are presented as the mean ± SD (n = 3).

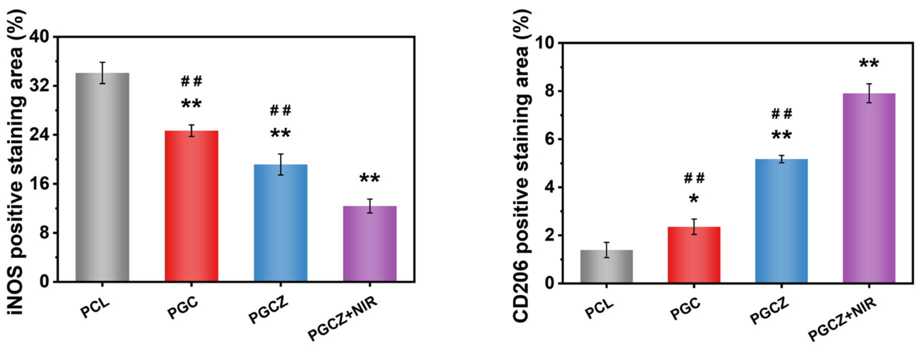

Figure S32. Quantitative analysis of iNOS- and CD206-positive staining areas after implantation. Data are presented as the mean ± SD (n = 3). *P < 0.05 and **P < 0.01 indicate significant differences compared with the PCL group. #P < 0.05 and # #P < 0.01 indicate significant differences compared with the PGCZ+NIR group.

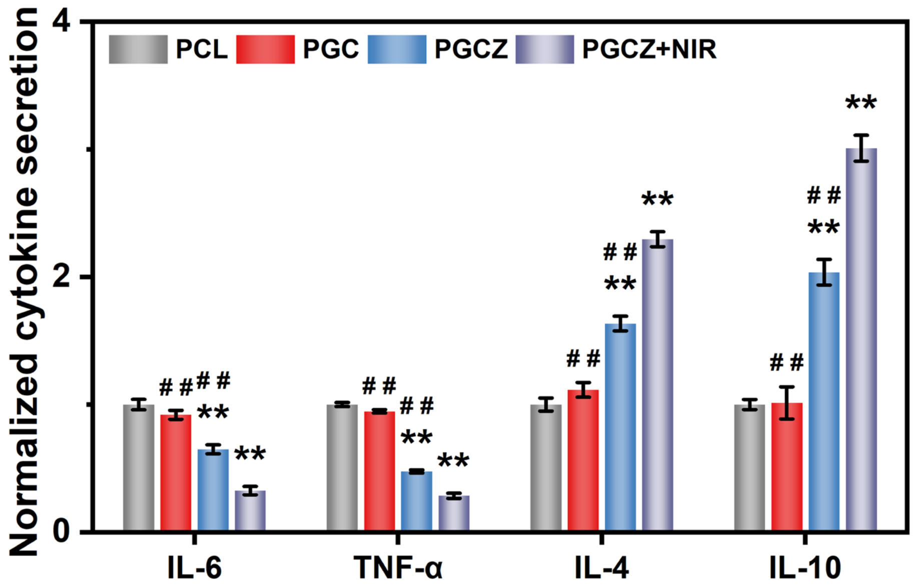

Figure S33. Secretion of proinflammatory (IL-6 and TNF-α) and anti-inflammatory (IL-4 and IL-10) cytokines induced by the scaffolds in vivo. Data are presented as the mean ± SD (n = 3). *P < 0.05 and **P < 0.01 indicate significant differences compared with the PCL group. #P < 0.05 and # #P < 0.01 indicate significant differences compared with the PGCZ+NIR group.

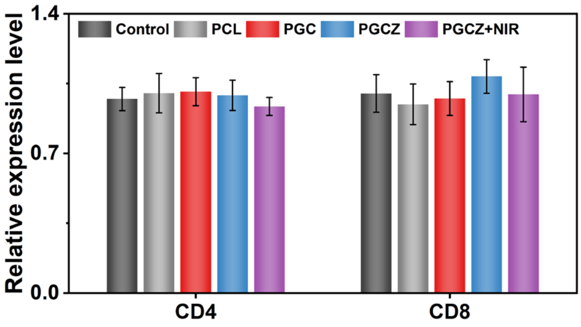

Figure S34. ELISA results of CD4 and CD8 in serum of rats. Data are presented as the mean ± SD (n = 3).

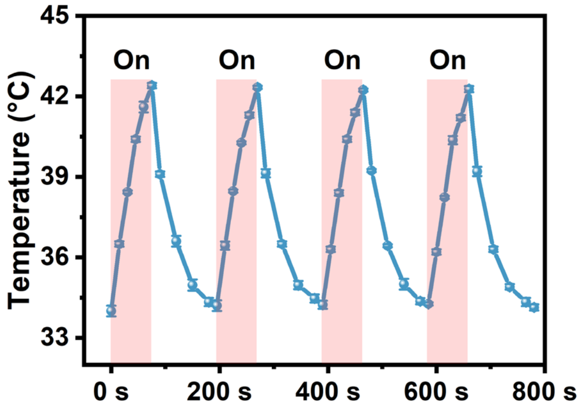

Figure S35. Photothermal heating curves of the implantation site under NIR irradiation (1 W/cm^2, 808 nm) with four on/off cycles. Data are presented as the mean ± SD (n = 3).

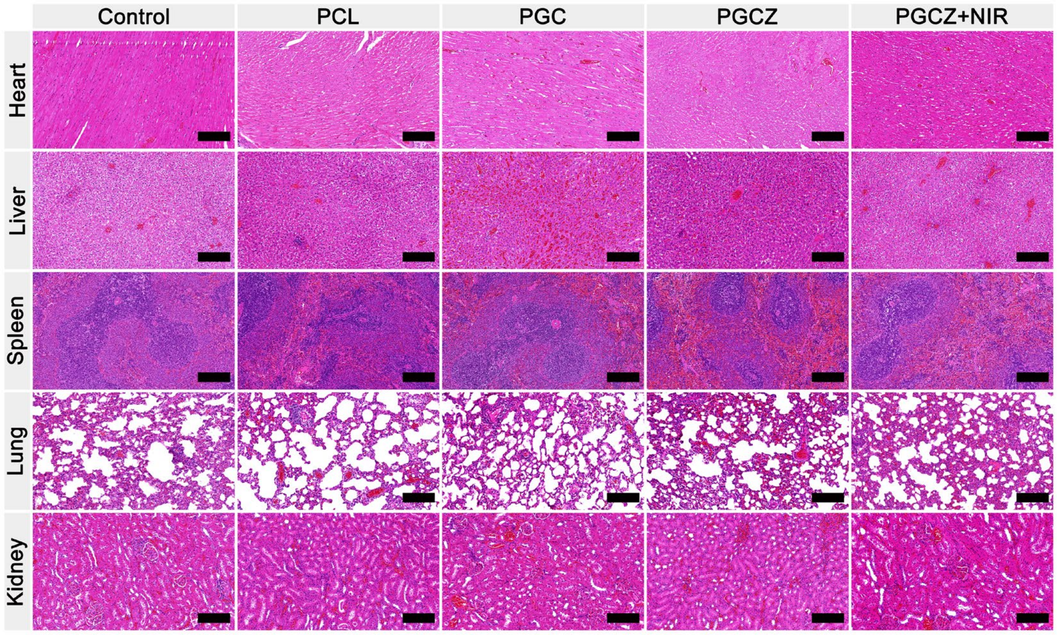

Figure S36. H&E staining images of the major organs, including the heart, liver, spleen,

lung, and kidney, in the different groups at 8 weeks. Scale bar: 200 μm.

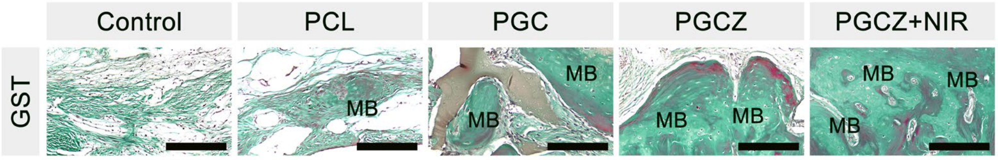

Figure S37. GST staining images of decalcified bone tissue at 8 weeks. MB: mature/mineralized bone. Scale bar: 200 μm.

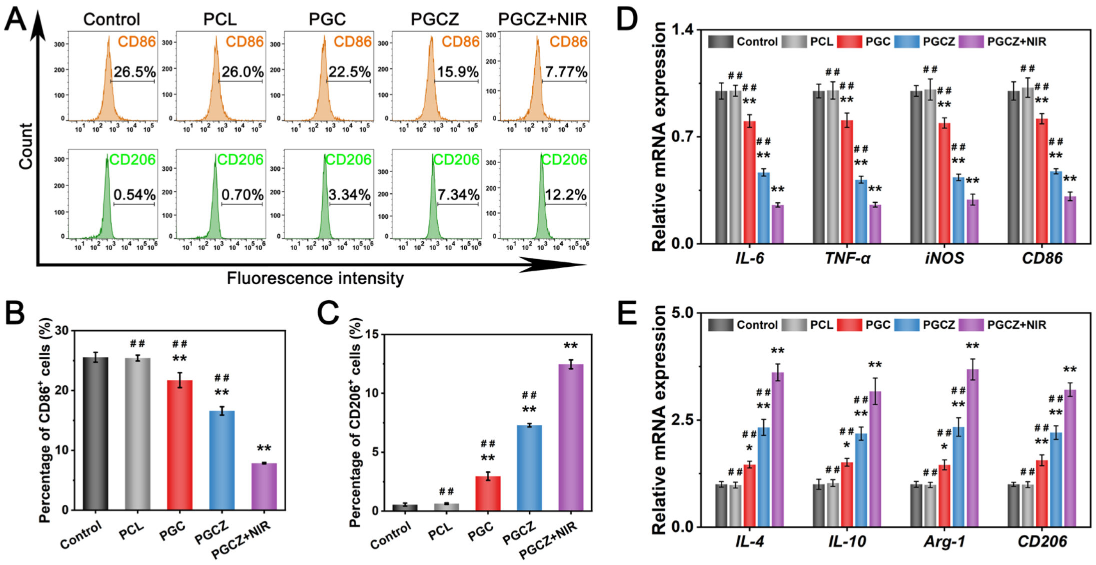

Figure S38. **(A)** Flow cytometry analysis and **(B-C)** corresponding quantification of macrophage phenotypes at 2 weeks after implantation. Relative mRNA expression of **(D)** proinflammatory and **(E)** anti-inflammatory markers at 2 weeks after implantation. Data are presented as the mean ± SD (n = 3). *P < 0.05 and **P < 0.01 indicate significant differences compared with the control group. #P < 0.05 and ##P < 0.01 indicate significant differences compared with the PGCZ+NIR group.

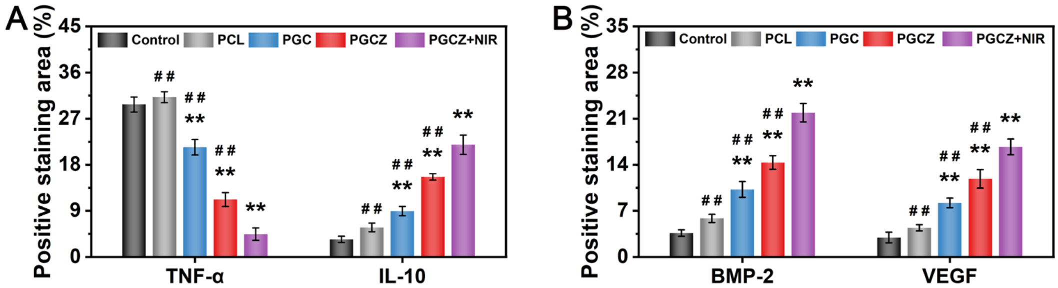

Figure S39. **(A)** Quantitative analysis of TNF-α and IL-10. **(B)** Quantitative analysis of BMP-2 and VEGF. Data are presented as the mean ± SD (n = 3). *P < 0.05 and **P < 0.01

indicate significant differences compared with the control group. $^{\#}P < 0.05$ and $^{\#\#}P < 0.01$ indicate significant differences compared with the PGCZ+NIR group.

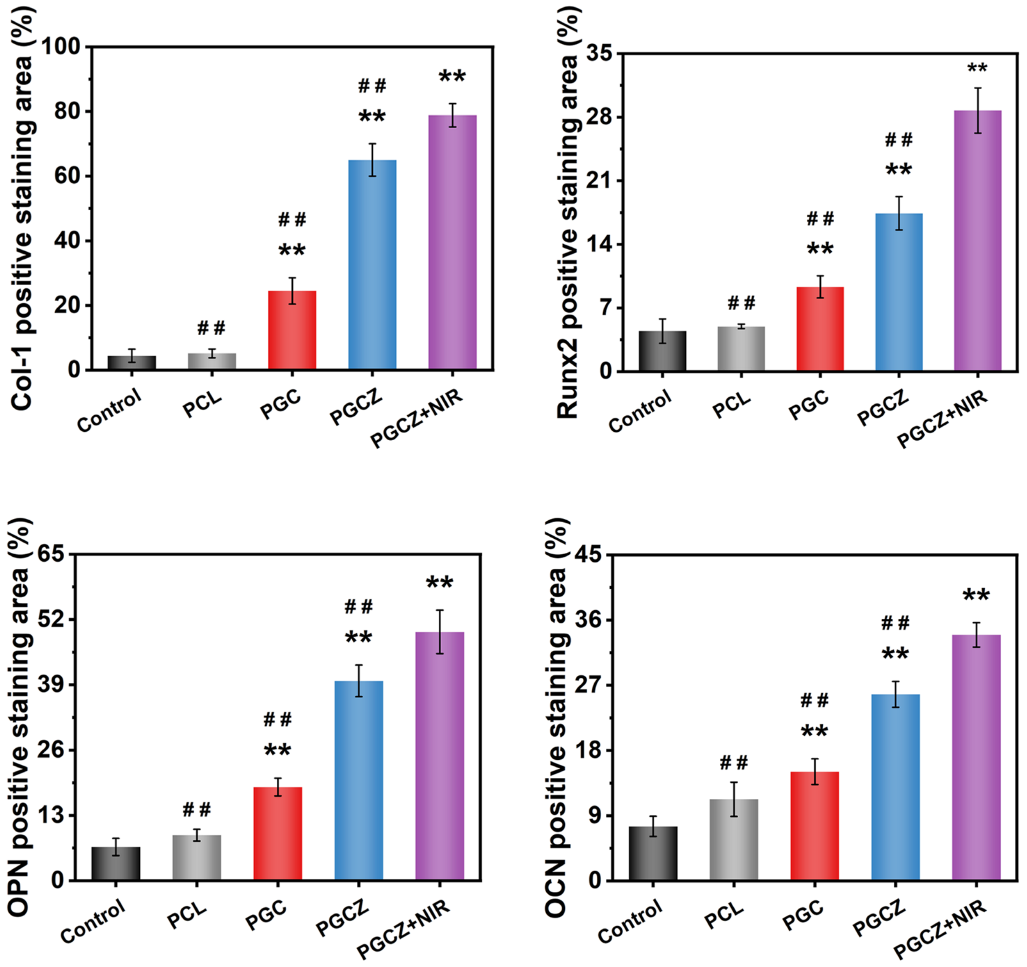

Figure S40. Quantitative analysis of immunohistochemical staining at 8 weeks after implantation. Data are presented as the mean ± SD (n = 3). $^{*}P < 0.05$ and $^{**}P < 0.01$ indicate significant differences compared with the control group. $^{\#}P < 0.05$ and $^{\#\#}P < 0.01$ indicate significant differences compared with the PGCZ+NIR group.

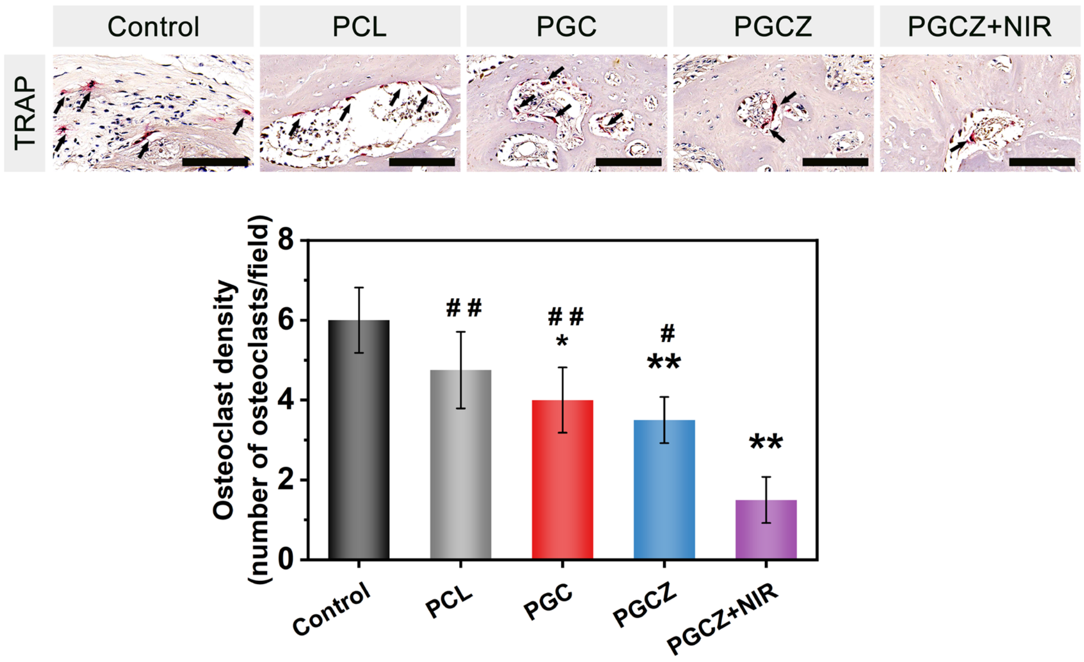

Figure S41. TRAP staining images of decalcified bone tissue and quantification of TRAP-positive cells. Scale bar: 100 μm. Data are presented as the mean ± SD (n = 3). *P < 0.05 and **P < 0.01 indicate significant differences compared with the control group. $^{\#}$P < 0.05 and $^{\#\#}$P < 0.01 indicate significant differences compared with the PGCZ+NIR group.

Table S1. Primer sequences used in qRT-PCR analysis.

Genes	Primers (F, forward; R, reverse; 5'-3')
Mouse-GADPH	F: TCAACGGCACAGTCAAGG<br>R: TTAGTGGGGTCTCGCTCC
Mouse-Runx2	F: CATCCCAGTATGAGAGTAGGTGT<br>R: GCTCAGATAGGAGGGGTAAGAC
Mouse-Col-1	F: CTGACTGGAAGAGCGGAGAG<br>R: CGGCTGAGTAGGGAACACAC
Mouse-OPN	F: TCTGAGGGACTAACTACGACCAT<br>R: TGGAAGAGTTTCTTGCTTAAAGTC
Mouse-OCN	F: TTCTGCTCACTCTGCTGACCC<br>R: CTGATAGCTCGTCACAAGCAGG
Mouse-TNF-α	F: CAGGCGGTGCCTATGTCTC<br>R: CGATCACCCCGAAGTTCAGTAG
Mouse-IL-6	F: GAGACCACTGGGGAGAATGC<br>R: TTGCCAGGTGGGTAAAGTGG
Mouse-iNOS	F: GAATCTTGGAGCGAGTTG<br>R: CCAGGAAGTAGGTGAGGG
Mouse-CD86	F: ATGGGCTCGTATGATTGT<br>R: TCTTAGGTTTCGGGTGAC
Mouse-IL-4	F: CATCCTGCTCTTCTTTCTC

	R: TTCTCCTGTGACCTCGTT
Mouse-IL-10	F: TTTCAAACAAAGGACCAG<br>R: GGATCATTTCCGATAAGG
Mouse-Arg-1	F: AAGACAGCAGAGGAGGTG<br>R: AGTCAGTCCCTGGCTTA
Mouse-CD206	F: GCAAGTGATTTGGAGGCT<br>R: ATAGGAAACGGGAGAACC
Rat-GADPH	F: CTCCCATTCTTCCACCTTTG<br>R: TGGTCCAGGGTTTCTTACT
Rat-CD86	F: CGAACACTATTTGGGCGCAG<br>R: CAAACTGGGGCTGCGAAAAA
Rat-IL-6	F: CCAGTTGCCTTCTTGGGACT<br>R: TCTGACAGTGCATCATCGCT
Rat-TNF-α	F: GCCTCTTCTCATTCCTGCTT<br>R: TGGGAACTTCTCATCCCTTTG
Rat-iNOS	F: GAGACGCACAGGCAGAGGTTG<br>R: AGCAGGCACACGCAATGATGG
Rat-IL-4	F: ACCTTGCTGTCACCCTGTTC<br>R: TTGTGAGCGTGGACTCATTC
Rat-Arg-1	F: GGACATCGTGTACATCGGCT<br>R: TTTGCTGTGATGCCCCAGAT
Rat-IL-10	F: TGCACCCACTTCCCAGTCAGC<br>R: CACCTGCTCCACTGCCTTGC
Rat-CD206	F: GACGGACGAGGAGTTCATTATACR<br>R: GTTGGAGAGATAGGCACAGAAG
Human-GAPDH	F: CATCATCCCTGCCTCTACTGG<br>R: GTGGGTGTCGCTGTTGAAGTC
Human-VEGF	F: TATGCGGATCAAACCTCACCA<br>R: CACAGGGATTTTTCTTGTCTTGCT
Human-HIF-1α	F: ATCCATGTGACCATGAGGAAAT<br>R: CTCGGCTAGTTAGGGTACACTT
Human-bFGF	F: AAGAGCGACCCTCACATCAA<br>R: GCCAGGTAACGGTTAGCACA
Human-Ang-1	F: CAGGAGGATGGTGGTTTG<br>R: GCCCTTTGAAGTAGTGCC
